# Computational Insight into Biotransformation Profiles of Organophosphorus Flame Retardants to Their Diester Metabolites by Cytochrome P450

**DOI:** 10.3390/molecules27092799

**Published:** 2022-04-28

**Authors:** Yue Jia, Tingji Yao, Guangcai Ma, Qi Xu, Xianglong Zhao, Hui Ding, Xiaoxuan Wei, Haiying Yu, Zhiguo Wang

**Affiliations:** 1 College of Geography and Environmental Sciences, Zhejiang Normal University, Jinhua 321004, China; jiayue@zjnu.edu.cn (Y.J.); yaotingji21@mails.ucas.ac.cn (T.Y.); xuqi7977@zjnu.edu.cn (Q.X.); 202025201155@zjnu.edu.cn (X.Z.); bubbledh@icloud.com (H.D.); xxwei@zjnu.edu.cn (X.W.); 2 School of Environment, Hangzhou Institute for Advanced Study, University of Chinese Academy of Sciences, Hangzhou 310024, China; 3 Institute of Ageing Research, School of Medicine, Hangzhou Normal University, Hangzhou 311121, China; zhgwang@aliyun.com

**Keywords:** organophosphorus flame retardant, P450 enzyme, biotransformation, density functional theory calculations, molecular dynamics simulations

## Abstract

Biotransformation of organophosphorus flame retardants (OPFRs) mediated by cytochrome P450 enzymes (CYPs) has a potential correlation with their toxicological effects on humans. In this work, we employed five typical OPFRs including tris(1,3-dichloro-2-propyl) phosphate (TDCIPP), tris(1-chloro-2-propyl) phosphate (TCIPP), tri(2-chloroethyl) phosphate (TCEP), triethyl phosphate (TEP), and 2-ethylhexyl diphenyl phosphate (EHDPHP), and performed density functional theory (DFT) calculations to clarify the CYP-catalyzed biotransformation of five OPFRs to their diester metabolites. The DFT results show that the reaction mechanism consists of Cα-hydroxylation and O-dealkylation steps, and the biotransformation activities of five OPFRs may follow the order of TCEP ≈ TEP ≈ EHDPHP > TCIPP > TDCIPP. We further performed molecular dynamics (MD) simulations to unravel the binding interactions of five OPFRs in the CYP3A4 isoform. Binding mode analyses demonstrate that CYP3A4-mediated metabolism of TDCIPP, TCIPP, TCEP, and TEP can produce the diester metabolites, while EHDPHP metabolism may generate *para*-hydroxyEHDPHP as the primary metabolite. Moreover, the EHDPHP and TDCIPP have higher binding potential to CYP3A4 than TCIPP, TCEP, and TEP. This work reports the biotransformation profiles and binding features of five OPFRs in CYP, which can provide meaningful clues for the further studies of the metabolic fates of OPFRs and toxicological effects associated with the relevant metabolites.

## 1. Introduction

To improve the fire resistance of household products and fulfill the increasingly rigorous flammability standards, flame retardants (FRs) are often used as additives in various consumer supplies, such as furniture foam, infant products, textiles, and electronics [1,2,3]. The polybrominated diphenyl ethers (PBDEs) have been widely used in polyurethane foam for many years as the mainstream FRs [4]. Since the mid-2000s, PBDEs have been phased out in many countries due to their bioaccumulation, persistence, and toxicity to humans, and thereafter the interest in the alternatives of PBDEs such as organophosphate flame retardants (OPFRs) has been growing [5,6,7]. With the rapid increase in OPFRs production in recent years, global consumption reached 500,000 tons in 2011 and was estimated to be 680,000 tons in 2015 [8]. Because OPFRs are usually water-soluble and are not covalently bound to materials, they thus can easily discharge into the environment through wear, volatilization, and dissolution during use [9,10].

OPFRs have been widely detected in various environmental media, such as indoor and outdoor air, water, soil, dust, and sediments [3,11,12,13,14,15,16,17], and the levels of OPFRs in the indoor environment are significantly higher than those of brominated flame retardants (BFRs) [18]. Humans may be exposed to OPFRs through breathing, skin contact, and dietary intake, which has potential adverse effects on health [19]. Actually, OPFRs have been detected in human hair, breast milk, and urine samples [20,21,22,23]. Toxicological studies have shown that the long-term exposure of OPFRs to animals may lead to adverse effects on their reproductive and endocrine system [24,25,26,27]. In the exposure experiment using a rat brain sphere in vitro model, the enhancive expressions of cytokine gene and receptor demonstrate that OPFRs may induce an inflammatory response [28]. Moreover, OPFRs exposure may reduce the proliferation and growth of human neural stem cells, rat neuronal growth, and network activity [29].

Cytochrome P450 enzymes (CYPs) are phase-I metabolic enzymes involving primarily in the biotransformation of xenobiotic compounds in diverse organisms, which may substantially change the toxicological and physicochemical properties of OPFRs through metabolism [30]. The OPFRs can be metabolized by CYPs to produce hydroxylated metabolite and diester metabolite via the competitive C-hydroxylation and O-dealkylation reactions [31,32]. Although the structural differences among OPFRs can result in various mono-/di-hydroxylated metabolites, diester may be produced as the common metabolite. For instance, both human and *Brevibacillus brevis* CYPs can metabolize triphenyl phosphate (TPHP) into diphenyl phosphate (DPHP) as the important metabolite, and CYP1A2 and CYP2E1 isoforms are mainly involved in the metabolism in human liver microsomes (HLMs) [6,33]. Incubation of tris(1,3-dichloro-2-propyl) phosphate (TDCIPP) with HLMs results in the formation of bis(1,3-dichloro-2-propyl) phosphate (BDCIPP) and other metabolites of oxidative dehalogenation [32], and CYP3A4 shows the highest activity toward TDCIPP metabolism [34]. Indeed, 2-ethylhexyl diphenyl phosphate (EHDPHP) can be transformed by human CYPs into mono- and di-hydroxylated metabolites, keto metabolites, and diphenyl phosphate (DPHP) as major phase-I outputs [35]. Chen et al. further reported CYP3A4 to be the major activating enzyme involved in EHDPHP metabolism [36]. Hou et al. reported the in vitro metabolism kinetics of tris(2-butoxyethyl) phosphate (TBOEP) and tris(n-butyl) phosphate (TNBP) and identified CYP3A4 and CYP1A as the major CYP isoforms catalyzing the metabolism in fish liver microsomes [37]. However, to date there are few studies have explored the CYP-mediated biotransformation mechanism and reactivity of OPFRs in-depth, and binding interactions between OPFRs and specific CYP isoforms remain unclear, and deserve further research.

In the past two decades, molecular modeling techniques, such as molecular dynamics (MD) simulations and quantum chemical calculations, have been extensively used to shed light on the binding and metabolism of xenobiotics in various biomacromolecules including CYPs [38,39,40,41,42,43]. In this work, five OPFRs detected widely in the environment, including TDCIPP, tris(1-chloro-2-propyl) phosphate (TCIPP), tris(2-chloroethyl) phosphate (TCEP), triethyl phosphate (TEP), and EHDPHP (Figure S1 in the Supplementary Materials), were employed to investigate the reaction mechanism leading to their diester metabolites through the density functional theory (DFT) calculations. Further MD simulations and binding free energy calculations were performed to predict the binding features and affinities of five OPFRs in CYP3A4, and to confirm the DFT results.

## 2. Computational Procedures

### 2.1. Model System

The reactive iron(IV)−oxo porphyrin species Compound I (Cpd I) of CYP was mimicked by a simplified model, Fe^4+^O^2−^(C_20_N_4_H_12_)^−1^(HS)^−1^, to unravel the mechanistic details of biotransformation of 5 OPFRs. Numerous previous studies have proven the high reliability and effectiveness of this simple model to accurately reproduce the electronic structures and reactivity of Cpd I [44,45,46,47].

### 2.2. DFT Methodology

All DFT calculations were performed by Gaussian 09 program [48]. The geometry optimizations and frequency analyses were carried out using the spin-unrestricted UB3LYP functional [49,50,51], integrating the LANL2DZ pseudopotential basis set [52] for the Fe atom and 6-31G(d,p) basis set [53] for other atoms (denoted BS1). Frequency analyses were used to obtain the thermal corrections to the Gibbs free energy, and to confirm the nature of all optimized geometries, among which all reactant/intermediate structures showed only real frequencies and the transition states had a single imaginary frequency. Based on the optimized geometries, single-point energy (SPE) calculations were performed using a larger def2-TZVP basis set [54] for all atoms (BS2) to obtain more accurate energies. Dispersion corrections were introduced into SPE calculations using the DFT-D3(BJ) method [55] to further improve the accuracies of B3LYP energies. Furthermore, the SMD solvation model [56] with nonpolar chlorobenzene and polar water was used to implicitly simulate CYP active site and aqueous-phase surroundings at the UB3LYP-D3/BS2 level, respectively. The relative energies reported in this work are thus the single-point energies with the inclusion of solvation and dispersion effects and Gibbs free energy corrections. It should be noted that Cpd I shows two closely lying spin states, including high-spin (HS) quartet and low-spin (LS) doublet states [57,58], and thus the energy profiles of biotransformation routes of 5 OPFRs were evaluated in both HS and LS states.

### 2.3. Molecular Docking

We selected CYP3A4 as the potential receptor of OPFRs because this isoform has a higher abundance and larger active pocket than the other CYPs in human liver tissue [59,60,61]. Moreover, in vitro experiment has identified CYP3A4 as the major CYP isoform catalyzing the metabolism of TDCIPP and EHDPHP [34,36]. CYP3A4 may also be involved in the metabolism of TCIPP, TCEP, and TEP due to the structural similarity of these 3 OPFRs with TDCIPP.

On the basis of the crystal structure of human 3A4 (PDB code: 2v0m) obtained from the Protein Data Bank (https://www.rcsb.org/, accessed on 15 April 2021) [59,62], molecular docking simulations were performed using Autodock Vina program [63] to construct the initial 3A4-OPFR binding complexes. Before docking, the bound ligand and water molecules in 3A4 were manually removed, and then the missing residues were complemented using the Chimera program [64]. During the docking, CYP3A4 was kept as the rigid receptor, while OPFRs were set as the flexible ligand. The grid box with suitable three dimensions was set to accommodate the ligand and CYP active site. The obtained 5 CYP3A4-OPFR complexes with the lowest binding affinities were shown in Figure S2, which were selected as the initial conformations for the following MD simulations.

### 2.4. Molecular Dynamics Simulations

All MD simulations were carried out using Amber12 program [65] with the ff14SB force field [66]. In CYP3A4, all Asp and Glu residues were set to be deprotonated, while all Lys and Arg were set to be protonated. The protonation states of all His residues were determined based on the predicted p*K*a values by the PDB2PQR Server [67] and the visual inspections of surrounding hydrogen-bonding networks. Specially, His30, His54, His65, His287, and His402 were singly N*_ε_*-protonated, His324 was singly N*_δ_*-protonated, and His267 was doubly N*_δ_*- and N*_ε_*-protonated. The force field parameters of 5 OPFRs were generated using the Antechamber module of AmberTools. All CYP3A4-OPFR complexes were solvated using the truncated octahedral TIP3P water box [68], and then the generated solvation models were neutralized by adding counterions. Before the production simulation, each solvated system was pre-equilibrated through 4000 ps energy minimization, 500 ps heating from 0 K to 300 K, 500 ps density equilibration at 300 K, and then 1000 ps constant pressure equilibration at 300 K. The final production simulations with different time scales were performed to equilibrate the solvated systems.

### 2.5. Binding Free Energy Calculations

After the production simulations, 200 snapshots were extracted from the last 20 ns MD trajectories to calculate the binding free energies (Δ*G*_bind_) between CYP3A4 and OPFRs. Based on the molecular mechanics/generalized Born surface area (MM/GBSA) method [69], Δ*G*_bind_ was calculated using the following Equations (1)–(4):

Δ*G*_bind_ = *G*_complex_ − (*G*_CYP3A4_ + *G*_OPFRs_) (1)

*G*_bind_ = *E*_MM_ + *G*_solv_ − *TS* (2)

*E*_MM_ = *E*_ele_ + *E*_vdW_ (3)

*G*_solv_ = *G*_GB_ + *G*_SA_ (4)

*G*_complex_, *G*_CYP3A4_, and *G*_OPFRs_ refer to the free energies of the binding complex, CYP3A4, and OPFRs, respectively. *E*_MM_ represents the molecular mechanics energy in the gas phase, consisting of electrostatic energy (*E*_ele_) and van der Waals interaction energy (*E*_vdW_). *G*_solv_ refers to the solvation free energy, which can be divided into a polar term (*G*_GB_) and a nonpolar term (*G*_SA_). *TS* is the entropy contribution. The calculated Δ*G*_bind_ can be used to compare the binding affinities of different OPFRs in CYP3A4. Furthermore, the active site residues contributing significantly to the ligand-binding were also identified by binding energy decomposition calculations (energy contribution < −1.0 kcal/mol), which is essential for understanding the molecular mechanism of ligand-receptor binding [70].

## 3. Results and Discussion

### 3.1. Biotransformation Profiles of OPFRs by CYP

Biotransformation of TDCIPP to BDCIPP. Previous studies have detected BDCIPP as the main product in the phase-I metabolism of TDCIPP [1,71,72], and CYPs may be involved in the metabolic process [32]. Our calculations confirm that the biotransformation of TDCIPP to BDCIPP proceeds through the Cpd I-mediated C-hydroxylation and intramolecular O-dealkylation processes. C-Hydroxylation begins with the hydrogen atom transfer (HAT) from C_α_ atom (denoted C_1_) of TDCIPP to Fe^IV^=O unit of Cpd I, generating an unstable C_1_-radical intermediate (IM_1-H_) with reduced Fe^IV^-OH species. C_1_-radical further captures OH from Fe^IV^-OH to form a hydroxylated intermediate (IM_1-OH_) with resting Fe^III^-porphyrin species. Subsequently, IM_1-OH_ undergoes O-dealkylation to produce the final diester metabolite BDCIPP (P_BDCIPP_) with 1,3-dichloroacetone.

**Figure 1:**
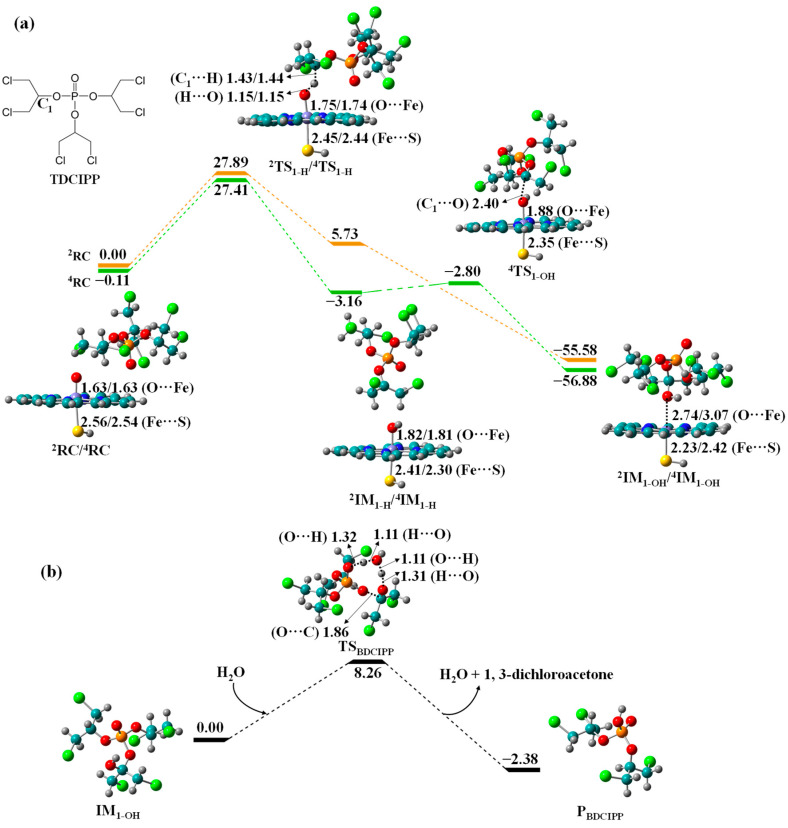
Free energy profiles for (**a**) C_1_-hydroxylation of TDCIPP to IM_1-OH_ by Cpd I and (**b**) O-dealkylation of IM_1-OH_ to BDCIPP, along with the optimized geometries of relevant reaction species in HS and LS states. Bond lengths are given in angstroms, while relative energies are given in kcal/mol.

**Figure 2:**
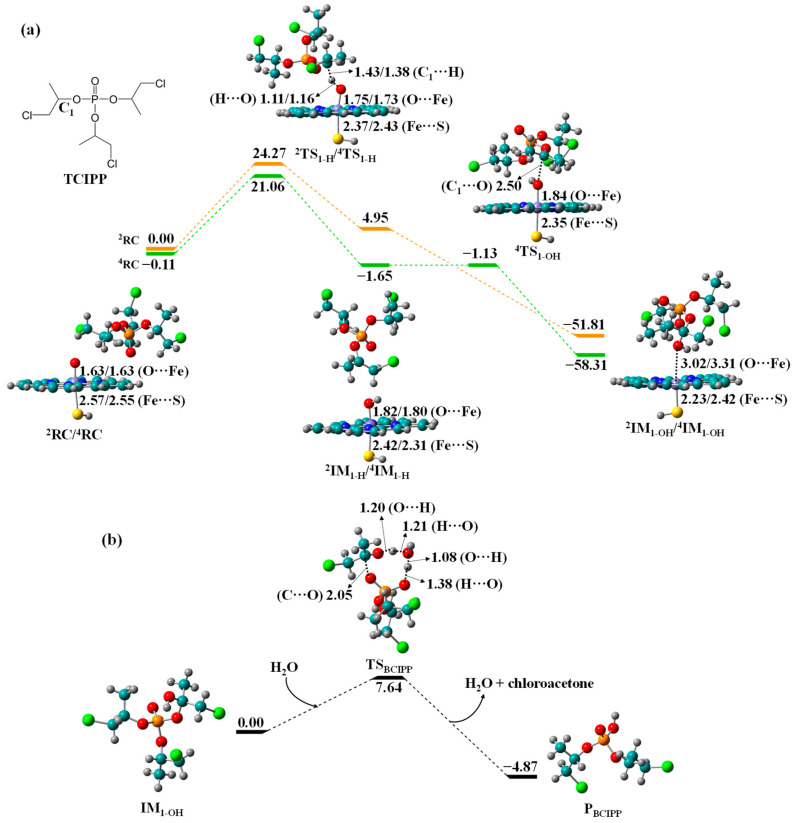
Free energy profiles for (**a**) C_1_-hydroxylation of TCIPP to IM_1-OH_ by Cpd I and (**b**) O-dealkylation of IM_1-OH_ to BCIPP, along with the optimized geometries of relevant reaction species in HS and LS states. Bond lengths are given in angstroms, while relative energies are given in kcal/mol.

The free energy profiles for biotransformation of TDCIPP and the optimized geometries of relevant reaction species in both HS and LS states are shown in Figure 1, and the structural characteristics of transition states of HAT and OH rebound are also summarized in Table S1. HAT proceeds through a transition state TS_1-H_ with an LS/HS energy barrier of 27.89/27.52 kcal/mol relative to the reactant complex (RC). The formation of intermediate IM_1-H_ is endothermic by 5.73 kcal/mol in the LS state, while it is exothermic by 3.05 kcal/mol in the HS state. The subsequent OH rebound to C_1_-radical is essentially a barrier-free process, although it undergoes a transition state ^4^TS_1-OH_ with a tiny barrier of 0.36 kcal/mol in the HS state. The collapse of IM_1-H_ to IM_1-OH_ is strongly exothermic, which facilitates the follow-up O-dealkylation of IM_1-OH_. As shown in Figure 2b, the intramolecular O-dealkylation proceeds through H_2_O-assisted proton transfer from OH to P=O group, undergoing a transition state TS_BDCIPP_ with a barrier of 8.26 kcal/mol to generate the exothermic P_BDCIPP_.

Biotransformation of TCIPP to BCIPP. TCIPP shows highly structural similarity with TDCIPP, and therefore they may have similar metabolic routes. Figure 2 shows the free energy profiles for biotransformation of TDCIPP to bis(1-chloro-2-propyl) phosphate (BCIPP). The LS and HS barriers of HAT are calculated to be 24.27 and 21.17 kcal/mol, respectively, which are lower than those in TDCIPP metabolism (27.89 and 27.52 kcal/mol). The barrier difference may be attributed to the chlorine substituent. Compared with TDCIPP, TCIPP has only one chlorine substituent in each alkyl side chain, which may reduce the steric hindrance effect and further enhance the reactivity of C_1_-hydroxylation. The barrier-free collapse of C_1_-radical intermediate IM_1-H_ results in the hydroxylated intermediate IM_1-OH_ with a dramatically exothermic effect (51.81 to 58.20 kcal/mol relative to the respective RCs). The H_2_O-assisted O-dealkylation of IM_1-OH_ undergoes transition state TS_BCIPP_ with an energy barrier of 7.64 kcal/mol to yield the exothermic product P_BCIPP_ with chloroacetone (Figure 2b).

**Figure 3:**
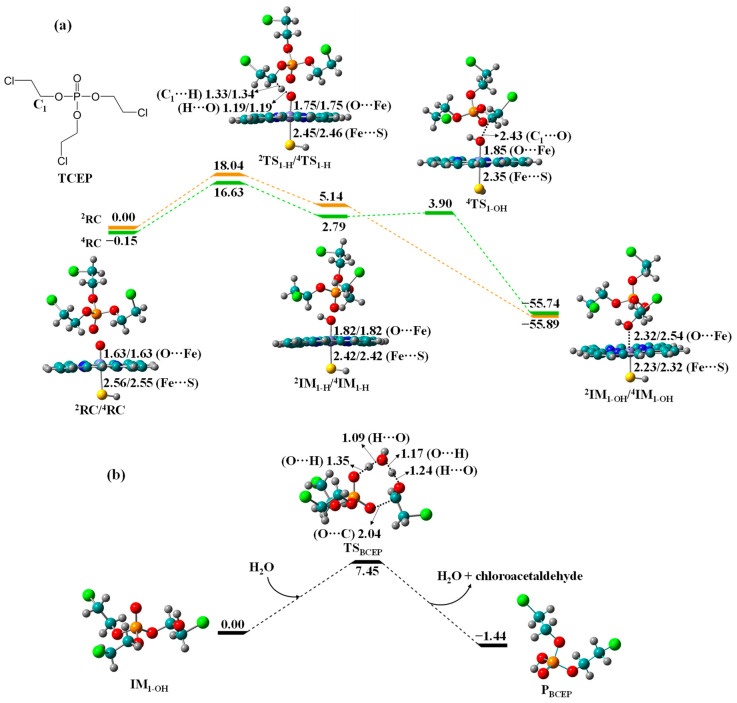
Free energy profiles for (**a**) C_1_-hydroxylation of TCEP to IM_1-OH_ by Cpd I and (**b**) O-dealkylation of IM_1-OH_ to BCEP, along with the optimized geometries of relevant reaction species in HS and LS states. Bond lengths are given in angstroms, while relative energies are given in kcal/mol.

Biotransformation of TCEP to BCEP. TCEP can undergo C_1_-hydroxylation and O-dealkylation to produce bis(2-chloroethyl) phosphate (BCEP) and chloroacetaldehyde as metabolites. The free energy profiles are shown in Figure 3. The LS/HS barrier of HAT is 18.04/16.78 kcal/mol, much smaller than those in TDCIPP (27.89/27.52 kcal/mol) and TCIPP (24.27/21.17 kcal/mol) biotransformation, suggesting that TCEP has higher metabolic activity to form the diester metabolite. After OH rebound, the resultant formation of IM_1-OH_ is strongly exothermic by 55.89/55.59 kcal/mol. Moreover, the O-dealkylation of IM_1-OH_ proceeds through transition state TS_BCEP_ with barrier of 7.45 kcal/mol to form the exothermic P_BCEP_.

**Figure 4:**
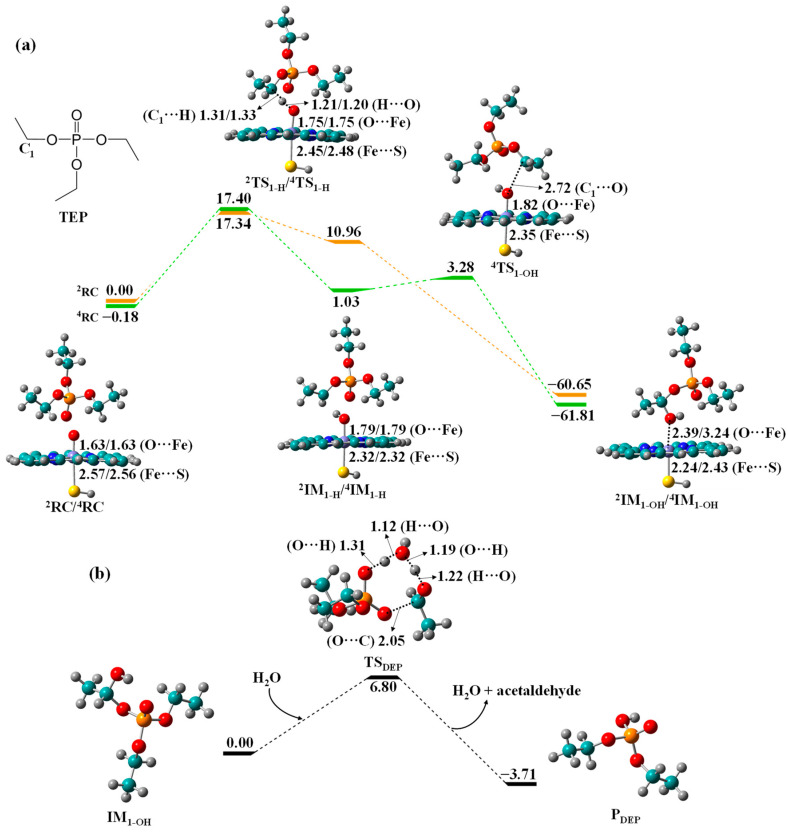
Free energy profiles for (**a**) C_1_-hydroxylation of TEP to IM_1-OH_ by Cpd I and (**b**) O-dealkylation of IM_1-OH_ to DEP, along with the optimized geometries of relevant reaction species in HS and LS states. Bond lengths are given in angstroms, while relative energies are given in kcal/mol.

Biotransformation of TEP to DEP. Compared with TCEP, TEP has no chlorine substituent in its ethyl groups. The HAT barrier of TEP is 17.34/17.58 kcal/mol in the LS/HS state (Figure 4), which is comparable with that of TCEP (18.04/16.78 kcal/mol), suggesting that chlorine substituent is incapable of differentiating the metabolic activities of TCEP and TEP. OH rebound in HS surface cross the transition state ^4^TS_1-OH_ with a minor barrier of 2.25 kcal/mol to yield exothermic C_1_-hydroxyTEP, followed by the H_2_O-triggered O-dealkylation of IM_1-OH_ to generate the exothermic diethyl phosphate (DEP) with acetaldehyde.

**Figure 5:**
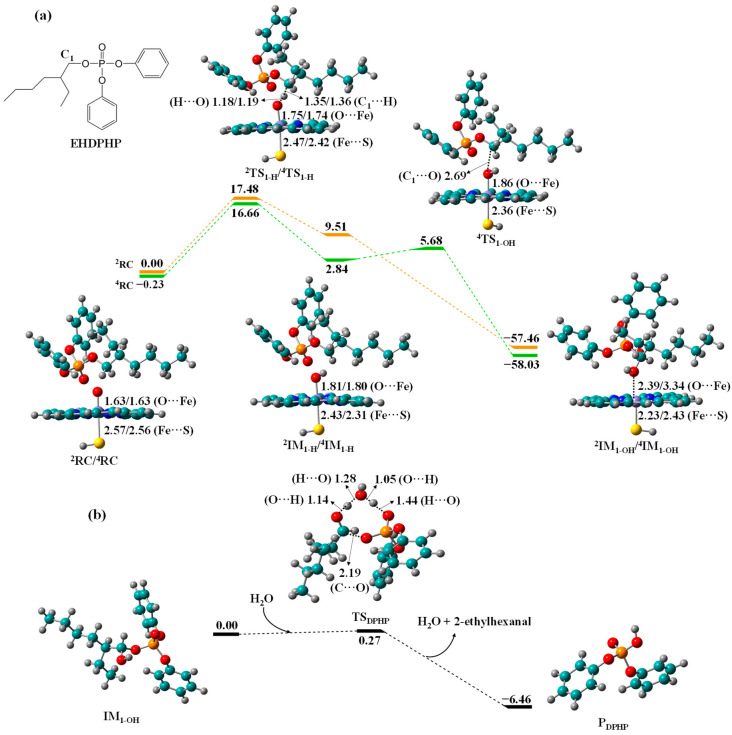
Free energy profiles for (**a**) C_1_-hydroxylation of EHDPHP to IM_1-OH_ by Cpd I and (**b**) O-dealkylation of IM_1-OH_ to DPHP, along with the optimized geometries of relevant reaction species in HS and LS states. Bond lengths are given in angstroms, while relative energies are given in kcal/mol.

Biotransformation of EHDPHP to DPHP. The free energy profiles of biotransformation of EHDPHP to DPHP are shown in Figure 5. The corresponding HAT barrier is 17.48/16.89 kcal/mol in the LS/HS surface, which has a slight difference with those of TCEP and TEP (18.04/16.78 and 17.34/17.58 kcal/mol). The results state clearly that these three OPFRs have comparable metabolic activities leading to their diester metabolites. The formation of remarkably exothermic IM_1-OH_ facilitates the ensuing O-dealkylation to produce exothermic DPHP with 2-ethylhexanal. Moreover, the C_1_-hydroxyEHDPHP has much smaller O-dealkylation barrier than the other 4 C_1_-hydroxyOPFRs (0.27 vs. 6.80 to 8.26 kcal/mol). It is notable that prior studies have confirmed that TPHP containing three phenyl groups can be transformed to diester metabolite DPHP by CYPs [6,32]. Given the structural similarity of EHDPHP with TPHP, it is also possible that EHDPHP metabolism produce 2-ethylhexyl phenyl phosphate (EHPHP) as another diester metabolite.

Overall, these results demonstrate that the HAT barriers are much higher than the O-dealkylation barriers for these 5 OPFRs, suggesting HAT to be the rate-determining step of biotransformation routes. The O-dealkylation of hydroxyOPFRs is a moderately exothermic process with a relatively low energy barrier, facilitating the formation of diester metabolites. In addition, a comparison of HAT barriers of 5 OPFRs reveals that the metabolic activity towards diester metabolites may follow the order of TCEP ≈ TEP ≈ EHDPHP > TCIPP > TDCIPP.

### 3.2. Binding Modes of 5 OPFRs in CYP3A4

**Figure 6:**
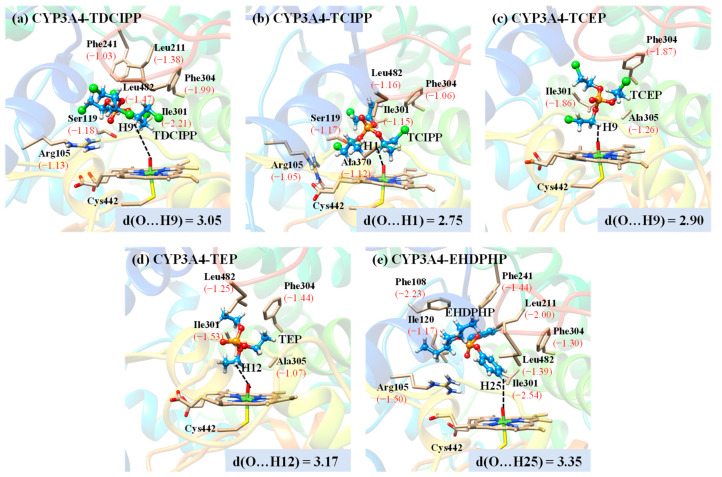
Averaged binding conformations of (**a**) TDCIPP, (**b**) TCIPP, (**c**) TCEP, (**d**) TEP, and (**e**) EHDPHP in CYP3A4 extracted from the last 20 ns MD trajectories. The key residues contributing to OPFRs binding are shown in the images, and corresponding binding energy contributions are given in kcal/mol and shown in the brackets. Distances are given in angstroms.

We further selected CYP3A4 isoform as a potential receptor of OPFRs and performed MD simulations to unravel the binding interactions between OPFRs and CYP3A4 and confirm the DFT results discussed above. We calculated the root-mean-square deviations (RMSDs) of CYP3A4 and the bound OPFRs to evaluate the conformational stability of the binding complexes. The smooth RMSD curves indicate the dynamic equilibrium of binding conformations along the simulation time (Figure S3). Figure 6 shows the averaged binding conformations extracted from the last 20 ns MD trajectories. Structural analyses of the binding conformations reveal that CYP3A4 can trap the flexible OPFRs into the active pocket, and thus it may have the potential to metabolize OPFRs.

Among the 5 binding complexes, OPFRs show different binding features. Except for EHDPHP, the remaining 4 OPFRs can orientate one of their C_α_ atoms (denoted C_1_ above) toward the Fe=O unit. As shown in Figure 6, the averaged distances between the H atom linked to C_α_ atoms and the O atom of the Fe=O unit are calculated to be 3.05, 2.75, 2.90, and 3.17 Å for TDCIPP, TCIPP, TCEP, and TEP, respectively, which state clearly that the C_α_ atoms of these 4 OPFRs are the preferred sites of metabolism by CYP3A4 to yield their C_α_-hydroxylated metabolites, followed by O-dealkylation to generate the diester metabolites. These results accord well with the DFT results discussed above. Interestingly, for the CYP3A4-EHDPHP complex, the C_α_ atom of the 2-ethylhexyl group of EHDPHP stays away from the Fe=O unit, rejecting undoubtedly the formation of DPHP metabolite. However, the averaged distance between the *para*-H atom of phenyl and the O atom of Fe=O is 3.35 Å, facilitating the preferential formation of *para*-hydroxyEHDPHP metabolite. Therefore, we conclude that DPHP is not the candidate metabolite involved in the CYP3A4-mediated metabolism of EHDPHP.

### 3.3. Binding Affinities of 5 OPFRs in CYP3A4

Based on the last 20 ns MD snapshots, we calculated the binding free energies (Δ*G*_bind_) to estimate the binding affinities of 5 OPFRs in CYP3A4. The calculated Δ*G*_bind_ and its energy components for 5 CYP3A4-OPFR complexes are shown in Table 1. Among the 5 OPFRs, EHDPHP has the most negative value of Δ*G*_bind_ (−26.71 kcal/mol), followed by TDCIPP (−25.02 kcal/mol), TCIPP (−20.70 kcal/mol), TCEP (−13.32 kcal/mol), and TEP (−12.59 kcal/mol). Consequently, the binding affinities of OPFRs in CYP3A4 follow the order of EHDPHP > TDCIPP > TCIPP > TCEP > TEP. The further energy decomposition of Δ*G*_bind_ indicates that the van der Waals interaction energies (Δ*E*_vdW_: −25.28 to −43.85 kcal/mol) possess the most significant contribution to OPFRs binding, followed by the nonpolar solvation free energies (Δ*G*_SA_: −4.02 to −6.88 kcal/mol) and electrostatic interaction energies (Δ*E*_ele_: −0.56 to −4.55 kcal/mol) that have only minor contributions. Instead, entropy component (*T*Δ*S*: −15.64 to −21.72 kcal/mol) and polar solvation free energies (Δ*G*_GB_: 2.92 to 6.30 kcal/mol) show negative contributions to OPFRs binding.

### 3.4. Effects of CYP3A4 Active Site Residues on OPFRs Binding

Based on the binding energy decomposition calculations, we further identified the key CYP3A4 residues contributing significantly to OPFRs binding (Figure 6). Residues with an energy contribution value of less than −1 kcal/mol are regarded as the key residues [73]. The binding conformations of 5 OPFRs have certain differences in the CYP3A4 active site, and only hydrophobic Ile301 and Phe304 are the shared residues contributing to the binding of these ligands. In particular, Ile301 residue gives the major driving force for the binding of TDCIPP, TEP, and EHDPHP with energy contributions of −2.21, −1.53, and −2.54 kcal/mol (Figure 6a,d,e). Besides, Arg105 residue plays important role in promoting the binding of TDCIPP, TCIPP, and EHDPHP, while it has no contribution to binding TCEP and TEP. Compared with TECP and TEP, TDCIPP and TCIPP have more complex Cl-substituted alkyl groups, and thus their binding involves more key residues. Seven residues provide important contributions to TDCIPP binding, including Arg105, Ser119, Leu211, Phe241, Ile301, Phe304, and Leu482 (Figure 6a). TCIPP has only one Cl substituent in each propyl group compared with TDCIPP, and six residues are considered to be important to its binding, including Arg105, Ser119, Ile301, Phe304, Ala370, and Leu482 (Figure 6b). Further analysis shows that 5 shared residues (Arg105, Ser119, Ile301, Phe304, and Leu482) contribute significantly to the binding of both TDCIPP and TCIPP, which suggests that the different Cl substitution effects can moderately differentiate their binding features. For CYP3A4-EHDPHP complex, a total of 8 key residues are identified to contribute EHDPHP binding, including Arg105 (−1.50 kcal/mol), Phe108 (−2.23 kcal/mol), Ile120 (−1.17 kcal/mol), Leu211 (−2.00 kcal/mol), Phe241 (−1.44 kcal/mol), Ile301 (−2.54 kcal/mol), Phe304 (−1.30 kcal/mol), and Leu482 (−1.39 kcal/mol) (Figure 6e). The number of key residues with significant contributions in the CYP3A4-EHDPHP complex is higher than that in other complexes, and thus EHDPHP shows a stronger binding affinity to CYP3A4. Moreover, EHDPHP orients one of its phenyl groups toward Cpd I and moves the other two side chains into a cavity formed by the hydrophobic residues. Actually, except for Arg105 and Ser119, the other key residues are hydrophobic in CYP3A4-OPFR complexes, which can provide a hydrophobic cavity to stabilize the ligands binding.

## 4. Conclusions

CYP-catalyzed biotransformation of OPFRs may alert their environmental behavior and toxicological effects, and thus the identification of biotransformation profiles and relevant metabolites can provide meaningful guidance for environmental risk assessment of OPFRs exposure. In this work, we first performed quantum chemical calculations to unravel the biotransformation mechanism of OPFRs to their diester metabolites. The results show that Cpd I-mediated biotransformation of OPFRs undergoes C_α_-hydroxylation comprising of rate-determining HAT and barrier-free OH rebound to generate C_α_-hydroxyOPFRs, followed by H_2_O-triggered O-dealkylation to yield the respective diester metabolites. Metabolic activities of OPFRs follow the order of TCEP ≈ TEP ≈ EHDPHP > TCIPP > TDCIPP. Compared with the energy profile of TCIPP biotransformation, the extra Cl substituent in TDCIPP enhances the HAT barrier and thus reduces the metabolic activity toward BDCIPP. However, the Cl substitution cannot differentiate the reactivity of TCEP and TEP.

We further performed MD simulations to explore the binding interactions of OPFRs in CYP3A4 at the molecular level. The MD results demonstrate that CYP3A4 can accommodate these 5 OPFRs with varying binding features. Binding mode analyses indicate that the C_α_ atoms of TDCIPP, TCIPP, TCEP, and TEP are the preferred sites of metabolism leading to their diester metabolites, while EHDPHP may have more preference for *para*-hydroxyEHDPHP metabolite rather than DPHP. Based on the binding free energy calculations, we estimate the binding affinities in the order of EHDPHP > TDCIPP > TCIPP > TCEP > TEP and van der Waals interactions contribute to the major driving forces for OPFRs binding. Furthermore, the hydrophobic residues in the CYP3A4 active site play a crucial role in stabilizing the OPFRs binding, among which Ile301 and Phe304 are the common key residues contributing to the binding. Overall, the present work reports the CYP-mediated biotransformation profiles of OPFRs to their diester metabolites, which may provide an essential understanding of the metabolic fates of OPFRs.

## Figures and Tables

**Table 1:** Binding free energies between CYP3A4 and OPFRs.

CYP	OPFRs	Energy Components (kcal/mol)					
		Δ*E*_ele_	Δ*E*_VDW_	Δ*G*_GB_	Δ*G*_SA_	*T*Δ*S*	Δ*G*_bind_
3A4	TDCIPP	−4.55	−40.92	6.30	−5.67	−19.82	−25.02
	TCIPP	−0.56	−35.35	2.92	−4.93	−17.22	−20.70
	TCEP	−3.15	−27.70	4.59	−4.23	−17.17	−13.32
	TEP	−2.44	−25.28	3.51	−4.02	−15.64	−12.59
	EHDPHP	−3.31	−43.85	5.61	−6.88	−21.72	−26.71

## Data Availability

Not applicable.

## Supplementary Materials

The following supporting information can be downloaded at: https://www.mdpi.com/article/10.3390/molecules27092799/s1, Figure S1: Structural diagram of 5 OPFRs used in this work; Figure S2: Optimal docking conformations of OPFRs in 3A4 active site; Figure S3: Root mean square deviations (RMSDs) of 5 CYP3A4-OPFR complexes; Table S1: Structural characteristics and activation energy barriers of the transition states of hydrogen atom transfer and OH rebound; Tables S2–S6: Absolute energies and relative energies for the reaction species involved in Cpd I-catalyzed biotransformation of OPFRs to diesters; Tables S7–S11: Calculated spin densities for the molecular species involved in OPFRs C_1_-hydroxylation; Cartesian coordinates of all DFT-optimized structures.

## Sample Availability

Not available.
