# Supplementary material for: Computational Insight into Biotransformation Profiles of Organophosphorus Flame Retardants to Their Diester Metabolites by Cytochrome P450
Source: Molecules. 2022 Apr 28;27(9):2799. doi: 10.3390/molecules27092799 (PMC9102461; doi:10.3390/molecules27092799)
Supplement: Supplementary file 1 [file molecules-27-02799-s001.zip › molecules-1702696-supplementary.pdf]

Supporting Information

Computational Insight into Biotransformation Profiles of Organophosphorus Flame Retardants to Their Diester Metabolites by Cytochrome P450

Yue Jia[1,†], Tingji Yao[1,2,†], Guangcai Ma[1,*], Qi Xu[1], Xianglong Zhao[1], Hui Ding[1], Xiaoxuan Wei[1], Haiying Yu[1,*], and Zhiguo Wang[3]

[1] College of Geography and Environmental Sciences, Zhejiang Normal University, Jinhua, 321004, China

[2] School of Environment, Hangzhou Institute for Advanced Study, University of Chinese Academy of Sciences, Hangzhou, 310024, China

[3] Institute of Ageing Research, School of Medicine, Hangzhou Normal University, Hangzhou 311121, China

[*] Corresponding author: Guangcai Ma; Haiying Yu
E-mail: magc@zjnu.edu.cn; yhy@zjnu.cn

[†] These authors contributed equally to this work.

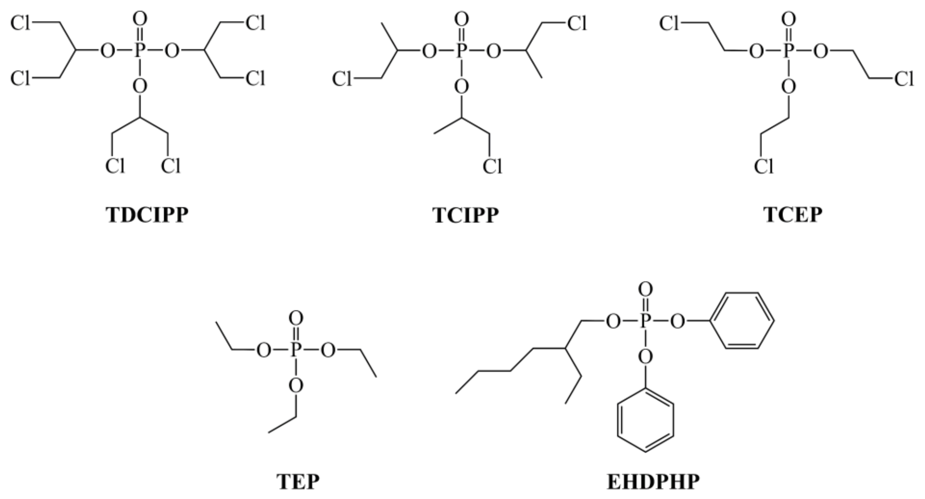

Figure S1. Structural diagram of 5 OPFRs used in this work.

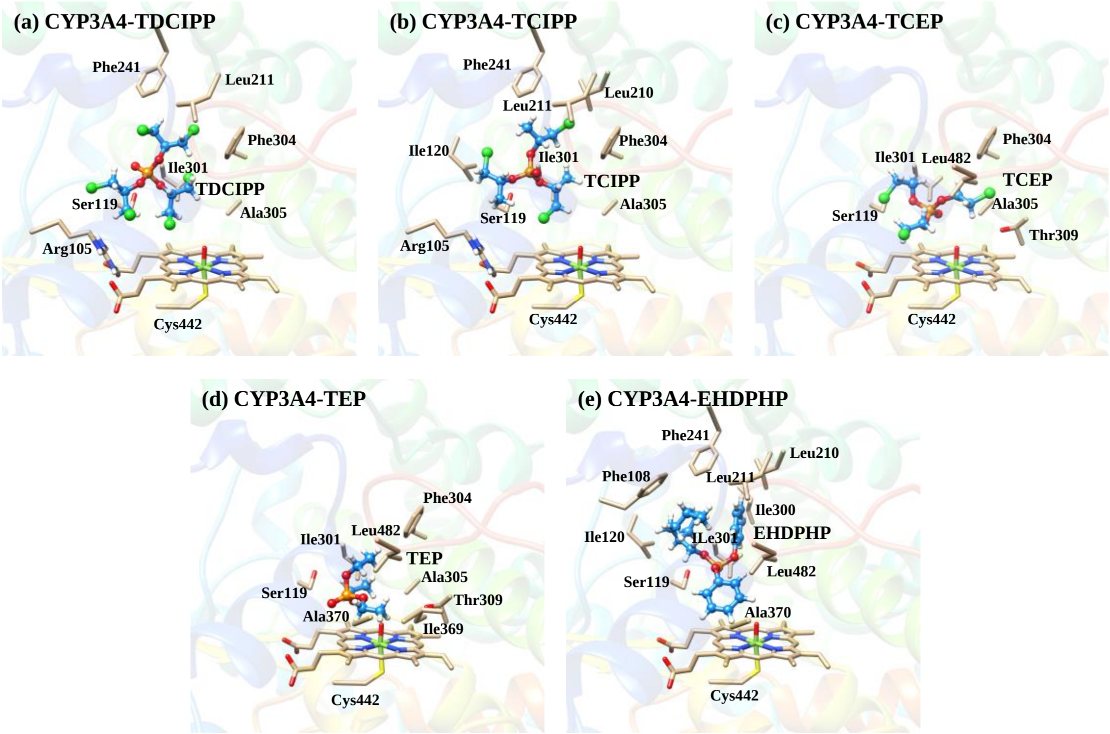

Figure S2. Optimal docking conformations of OPFRs in 3A4 active site.

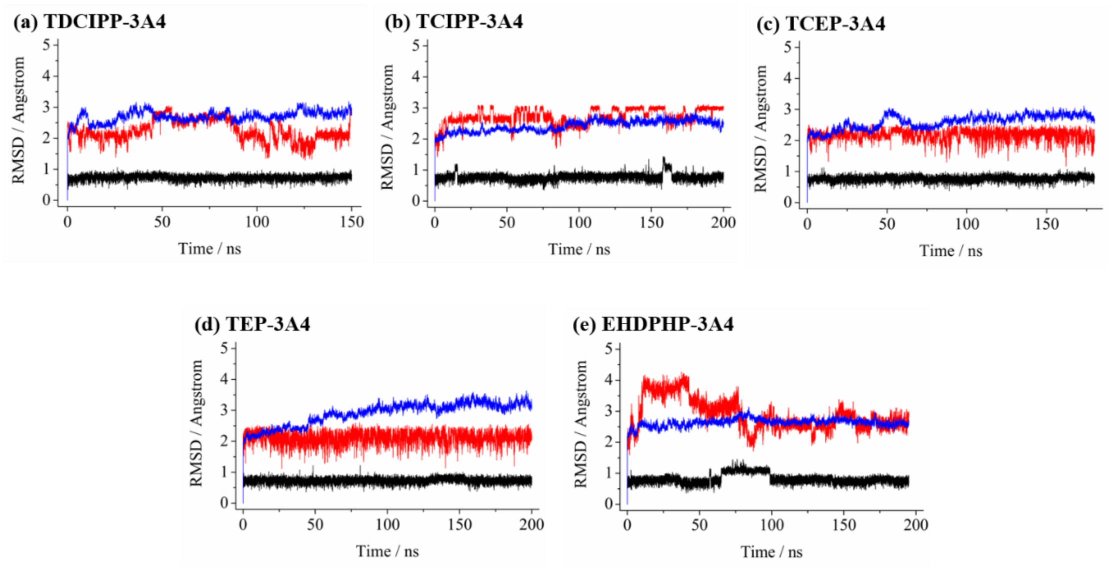

Figure S3. Root mean square deviations (RMSDs) of 5 CYP3A4-OPFR complexes. The RMSD curves of CYP, HEM and OPFR in each binding complex are colored in blue, black and red, respectively.

Table S1. Structural characteristics and activation energy barriers of the transition states of hydrogen atom transfer ($^{2/4}TS_{1\text{-}H}$) and OH rebound ($^{4}TS_{1\text{-}OH}$) involved in CYP-mediated Cα-hydroxylation of OPFRs in LS/HS state.

Chemicals	Transition states	$r_{O–Fe}$ (Å)	$r_{Fe–S}$ (Å)	$r_{C1–H}$ (Å)	$r_{H–O}$ (Å)	$r_{C1–O}$ (Å)	Energy barriers (kcal/mol)
TDCIPP	$^{2/4}TS_{1\text{-}H}$	1.75/1.74	2.45/2.44	1.43/1.44	1.15/1.15		27.89/27.52
	$^{4}TS_{1\text{-}OH}$	1.88	2.35			2.40	0.36
TCIPP	$^{2/4}TS_{1\text{-}H}$	1.75/1.73	2.37/2.43	1.43/1.38	1.11/1.16		24.27/21.17
	$^{4}TS_{1\text{-}OH}$	1.84	2.35			2.50	0.52
TCEP	$^{2/4}TS_{1\text{-}H}$	1.75/1.75	2.45/2.46	1.33/1.34	1.19/1.19		18.04/16.78
	$^{4}TS_{1\text{-}OH}$	1.85	2.35			2.43	1.11
TEP	$^{2/4}TS_{1\text{-}H}$	1.75/1.75	2.45/2.48	1.31/1.33	1.21/1.20		17.34/17.58
	$^{4}TS_{1\text{-}OH}$	1.82	2.35			2.72	2.25
EHDPHP	$^{2/4}TS_{1\text{-}H}$	1.75/1.74	2.47/2.42	1.35/1.36	1.18/1.19		17.4816.89
	$^{4}TS_{1\text{-}OH}$	1.86	2.36			2.69	2.74

Table S2. The absolute energies (AE: a.u.) and relative energies (RE: kcal/mol) for the reaction species involved in Cpd I-catalyzed biotransformation of TDCIPP to BDCIPP in both LS and HS states. The Gibbs free energy corrections (G_{corr}) were obtained at the UB3LYP/BS1 level, while the single-point energies (SPE) corrected for solvation (SMD) and dispersion (D3) effects were obtained at the UB3LYP-D3/BS2 level. The free energy barriers for C_1-hydroxylation are given relative to the reactant complex ^{2}RC, while the free energy barriers for O-dealkylation are given relative to $\mathbf{IM_{1\text{-}OH}}$.

**Reaction Routes**	**Reaction species**	**AE (G_{corr})**	**AE (SPE + SMD + D3 + G_{corr})**	**RE (SPE + SMD + D3 + G_{corr})**
C_1-hydroxylation	^{2}RC	0.448067	-6482.41776081	0.00
	^{4}RC	0.447619	-6482.41793843	-0.11
	$^2TS_{1\text{-}H}$	0.441412	-6482.37330867	27.89
	$^4TS_{1\text{-}H}$	0.443391	-6482.37407956	27.41
	$^2IM_{1\text{-}H}$	0.444907	-6482.40863706	5.73
	$^4IM_{1\text{-}H}$	0.440841	-6482.42280556	-3.16
	$^4TS_{1\text{-}OH}$	0.446565	-6482.42221846	-2.80
	$^2IM_{1\text{-}OH}$	0.453337	-6482.50633125	-55.58
	$^4IM_{1\text{-}OH}$	0.446874	-6482.50840708	-56.88
O-dealkylation	$IM_{1\text{-}OH}$	0.216586	-3907.66560805	0.00
	TS_{BDCIPP}	0.208833	-3907.65244086	8.26
	P_{BDCIPP}	0.206153	-3907.66940822	-2.38

Table S3. The absolute energies (AE: a.u.) and relative energies (RE: kcal/mol) for the reaction species involved in Cpd I-catalyzed biotransformation of TCIPP to BCIPP in both LS and HS states. The Gibbs free energy corrections (G_{corr}) were obtained at the UB3LYP/BS1 level, while the single-point energies (SPE) corrected for solvation (SMD) and dispersion (D3) effects were obtained at the UB3LYP-D3/BS2 level. The free energy barriers for C_1-hydroxylation are given relative to the reactant complex ^{2}RC, while the free energy barriers for O-dealkylation are given relative to $\mathbf{IM_{1\text{-}OH}}$.

**Reaction Routes**	**Reaction species**	**AE (G_{corr})**	**AE (SPE + SMD + D3 + G_{corr})**	**RE (SPE + SMD + D3 + G_{corr})**
C_1-hydroxylation	^{2}RC	0.477908	-5103.50835176	0.00
	^{4}RC	0.477486	-5103.50852205	-0.11
	$^2TS_{1\text{-}H}$	0.475240	-5103.46968160	24.27
	$^4TS_{1\text{-}H}$	0.473845	-5103.47479239	21.06
	$^2IM_{1\text{-}H}$	0.473590	-5103.50046726	4.95
	$^4IM_{1\text{-}H}$	0.474398	-5103.51096987	-1.65
	$^4TS_{1\text{-}OH}$	0.480594	-5103.51014352	-1.13
	$^2IM_{1\text{-}OH}$	0.482893	-5103.59092417	-51.81
	$^4IM_{1\text{-}OH}$	0.479202	-5103.60127068	-58.31
O-dealkylation	$IM_{1\text{-}OH}$	0.249993	-2528.75928731	0.00
	TS_{BCIPP}	0.241325	-2528.74711840	7.64
	P_{BCIPP}	0.238830	-2528.76705515	-4.87

Table S4. The absolute energies (AE: a.u.) and relative energies (RE: kcal/mol) for the reaction species involved in Cpd I-catalyzed biotransformation of TCEP to BCEP in both LS and HS states. The Gibbs free energy corrections (G_{corr}) were obtained at the UB3LYP/BS1 level, while the single-point energies (SPE) corrected for solvation (SMD) and dispersion (D3) effects were obtained at the UB3LYP-D3/BS2 level. The free energy barriers for C_1-hydroxylation are given relative to the reactant complex ^{2}RC, while the free energy barriers for O-dealkylation are given relative to $\mathbf{IM_{1\text{-}OH}}$.

**Reaction Routes**	**Reaction species**	**AE (G_{corr})**	**AE (SPE + SMD + D3 + G_{corr})**	**RE (SPE + SMD + D3 + G_{corr})**
C_1-hydroxylation	^{2}RC	0.398261	-4985.57012391	0.00
	^{4}RC	0.397658	-4985.57036922	-0.15
	$^2TS_{1\text{-}H}$	0.395249	-4985.54137652	18.04
	$^4TS_{1\text{-}H}$	0.393660	-4985.54362895	16.63
	$^2IM_{1\text{-}H}$	0.395837	-4985.56193190	5.14
	$^4IM_{1\text{-}H}$	0.393764	-4985.56567226	2.79
	$^4TS_{1\text{-}OH}$	0.400719	-4985.56391577	3.90
	$^2IM_{1\text{-}OH}$	0.410343	-4985.65919114	-55.89
	$^4IM_{1\text{-}OH}$	0.400737	-4985.65894690	-55.74
O-dealkylation	$IM_{1\text{-}OH}$	0.168938	-2410.81839592	0.00
	TS_{BCEP}	0.162500	-2410.80652229	7.45
	P_{BCEP}	0.161565	-2410.82069503	-1.44

Table S5. The absolute energies (AE: a.u.) and relative energies (RE: kcal/mol) for the reaction species involved in Cpd I-catalyzed biotransformation of TEP to DEP in both LS and HS states. The Gibbs free energy corrections (G_{corr}) were obtained at the UB3LYP/BS1 level, while the single-point energies (SPE) corrected for solvation (SMD) and dispersion (D3) effects were obtained at the UB3LYP-D3/BS2 level. The free energy barriers for C_1-hydroxylation are given relative to the reactant complex ^{2}RC, while the free energy barriers for O-dealkylation are given relative to $\mathbf{IM_{1\text{-}OH}}$.

**Reaction Routes**	**Reaction species**	**AE (G_{corr})**	**AE (SPE + SMD + D3 + G_{corr})**	**RE (SPE + SMD + D3 + G_{corr})**
C_1-hydroxylation	^{2}RC	0.431921	-3606.64425200	0.00
	^{4}RC	0.431294	-3606.64453833	-0.18
	$^2TS_{1\text{-}H}$	0.429202	-3606.61661843	17.34
	$^4TS_{1\text{-}H}$	0.427548	-3606.61652357	17.40
	$^2IM_{1\text{-}H}$	0.431156	-3606.62679318	10.96
	$^4IM_{1\text{-}H}$	0.430493	-3606.64260692	1.03
	$^4TS_{1\text{-}OH}$	0.429274	-3606.63902940	3.28
	$^2IM_{1\text{-}OH}$	0.440356	-3606.74089853	-60.65
	$^4IM_{1\text{-}OH}$	0.432274	-3606.74274642	-61.81
O-dealkylation	$IM_{1\text{-}OH}$	0.203158	-1031.90288473	0.00
	TS_{DEP}	0.195211	-1031.89204360	6.80
	P_{DEP}	0.194413	-1031.90880095	-3.71

Table S6. The absolute energies (AE: a.u.) and relative energies (RE: kcal/mol) for the reaction species involved in Cpd I-catalyzed biotransformation of EHDPHP to DPHP in both LS and HS states. The Gibbs free energy corrections (G_{corr}) were obtained at the UB3LYP/BS1 level, while the single-point energies (SPE) corrected for solvation (SMD) and dispersion (D3) effects were obtained at the UB3LYP-D3/BS2 level. The free energy barriers for C_1-hydroxylation are given relative to the reactant complex ^{2}RC, while the free energy barriers for O-dealkylation are given relative to **IM_{1-OH}**.

**Reaction Routes**	**Reaction species**	**AE (G_{corr})**	**AE (SPE + SMD + D3 + G_{corr})**	**RE (SPE + SMD + D3 + G_{corr})**
C_1-hydroxylation	^{2}RC	0.634001	-4147.44064210	0.00
	^{4}RC	0.633266	-4147.44100546	-0.23
	$^2TS_{1-H}$	0.630615	-4147.41279059	17.48
	$^4TS_{1-H}$	0.631127	-4147.41409525	16.66
	$^2IM_{1-H}$	0.630427	-4147.42548660	9.51
	$^4IM_{1-H}$	0.631692	-4147.43611805	2.84
	$^4TS_{1-OH}$	0.631857	-4147.43158954	5.68
	$^2IM_{1-OH}$	0.640326	-4147.53221087	-57.46
	$^4IM_{1-OH}$	0.633566	-4147.53310906	-58.03
O-dealkylation	IM_{1-OH}	0.402296	-1572.67651188	0.00
	TS_{DPHP}	0.397670	-1572.67608513	0.27
	P_{DPHP}	0.394285	-1572.68680377	-6.46

Table S7. Calculated spin densities for the molecular species involved in TDCIPP C_1-hydroxylation at the SMD-B3LYP-D3/BS2 level.

	**Cpd I ($Fe^{4+}O^{2-}Por^{-}SH^{-}$)**				**Substrate**		
	**Fe**	**O**	**SH**	**Por**	**Sub•**	**C_1**	**H**
**^{2}RC**	1.39	0.74	-0.44	-0.69	0.00	0.00	0.00
**^{4}RC**	1.26	0.79	0.38	0.56	0.00	0.00	0.00
**$^2TS_{1\text{-}H}$**	1.65	0.30	-0.28	-0.68	0.02	0.02	-0.01
**$^4TS_{1\text{-}H}$**	1.33	0.50	0.28	0.43	0.45	0.40	-0.04
**$^2IM_{1\text{-}H}$**	0.96	0.12	0.24	0.68	-1.00	-0.70	0.00
**$^4IM_{1\text{-}H}$**	1.97	0.16	0.02	-0.15	1.01	0.70	0.00
**$^4TS_{1\text{-}OH}$**	2.20	0.03	0.07	-0.15	0.85	0.63	0.01
**$^2IM_{1\text{-}OH}$**	1.18	0.00	-0.06	-0.12	0.00	0.00	0.00
**$^4IM_{1\text{-}OH}$**	2.68	0.00	0.37	-0.05	0.00	0.00	0.00

Table S8. Calculated spin densities for the molecular species involved in TCIPP C_1-hydroxylation at the SMD-B3LYP-D3/BS2 level.

	**Cpd I ($Fe^{4+}O^{2-}Por^{-}SH^{-}$)**				**Substrate**		
	**Fe**	**O**	**SH**	**Por**	**Sub•**	**C_1**	**H**
**^{2}RC**	1.37	0.76	-0.46	-0.67	0.00	0.00	0.00
**^{4}RC**	1.24	0.81	0.40	0.54	0.00	0.00	0.00
**$^2TS_{1\text{-}H}$**	1.24	0.22	-0.15	-0.44	0.13	0.11	-0.01
**$^4TS_{1\text{-}H}$**	1.52	0.54	0.28	0.11	0.56	0.45	-0.03
**$^2IM_{1\text{-}H}$**	1.04	0.04	-0.27	-0.81	1.00	0.65	0.00
**$^4IM_{1\text{-}H}$**	1.95	0.18	0.02	-0.15	1.01	0.53	0.00
**$^4TS_{1\text{-}OH}$**	2.16	0.06	0.07	-0.15	0.87	0.66	0.01
**$^2IM_{1\text{-}OH}$**	1.19	0.00	-0.07	-0.13	0.00	0.00	0.00
**$^4IM_{1\text{-}OH}$**	2.66	0.00	0.37	-0.03	0.00	0.00	0.00

Table S9. Calculated spin densities for the molecular species involved in TCEP C_1-hydroxylation at the SMD-B3LYP-D3/BS2 level.

	Cpd I ($Fe^{4+}O^{2-}Por^{-}SH^{-}$)				Substrate		
	**Fe**	**O**	**SH**	**Por**	**Sub•**	**C_1**	**H**
**^{2}RC**	1.38	0.75	-0.47	-0.66	0.00	0.00	0.00
**^{4}RC**	1.25	0.80	0.42	0.53	0.01	0.00	0.00
**$^2TS_{1-H}$**	1.56	0.28	-0.26	-0.56	-0.02	0.00	-0.01
**$^4TS_{1-H}$**	1.06	0.58	0.28	0.64	0.44	0.38	-0.05
**$^2IM_{1-H}$**	0.94	0.12	0.25	0.69	-1.00	-0.76	0.00
**$^4IM_{1-H}$**	2.14	0.17	-0.16	-0.16	1.00	0.77	0.01
**$^4TS_{1-OH}$**	2.18	0.04	0.07	-0.15	0.86	0.65	0.01
**$^2IM_{1-OH}$**	1.13	0.00	-0.03	-0.10	0.00	0.00	0.00
**$^4IM_{1-OH}$**	2.71	0.00	0.36	-0.08	0.01	0.00	0.00

Table S10. Calculated spin densities for the molecular species involved in TEP C_1-hydroxylation at the SMD-B3LYP-D3/BS2 level.

	Cpd I ($Fe^{4+}O^{2-}Por^{-}SH^{-}$)				Substrate		
	**Fe**	**O**	**SH**	**Por**	**Sub•**	**C_1**	**H**
**^{2}RC**	1.36	0.76	-0.46	-0.66	0.00	0.00	0.00
**^{4}RC**	1.24	0.81	0.41	0.53	0.01	0.00	0.00
**$^2TS_{1-H}$**	1.39	0.29	-0.25	-0.46	0.02	0.04	-0.01
**$^4TS_{1-H}$**	0.99	0.59	0.30	0.63	0.48	0.43	-0.04
**$^2IM_{1-H}$**	0.20	0.19	-0.29	0.01	0.89	0.77	0.01
**$^4IM_{1-H}$**	1.93	0.20	0.03	-0.15	0.99	0.85	0.02
**$^4TS_{1-OH}$**	2.12	0.10	0.04	-0.16	0.89	0.81	0.01
**$^2IM_{1-OH}$**	1.15	0.00	-0.04	-0.10	0.00	0.00	0.00
**$^4IM_{1–OH}$**	2.68	0.00	0.36	-0.04	0.00	0.00	0.00

Table S11. Calculated spin densities for the molecular species involved in EHDPHP C_1-hydroxylation at the SMD-B3LYP-D3/BS2 level.

	**Cpd I ($Fe^{4+}O^{2-}Por^{-}SH^{-}$)**				**Substrate**		
	**Fe**	**O**	**SH**	**Por**	**Sub•**	**C_1**	**H**
**^{2}RC**	1.38	0.75	-0.47	-0.66	0.00	0.00	0.00
**^{4}RC**	1.25	0.80	0.41	0.53	0.01	0.00	0.00
**$^2TS_{1-H}$**	1.22	0.29	-0.17	-0.49	0.15	0.15	-0.02
**$^4TS_{1-H}$**	1.54	0.55	0.28	0.08	0.56	0.46	-0.02
**$^2IM_{1-H}$**	0.97	0.10	0.28	0.61	-0.96	-0.88	0.00
**$^4IM_{1-H}$**	1.95	0.18	0.03	-0.15	0.99	0.90	0.01
**$^4TS_{1-OH}$**	2.21	0.05	0.05	-0.16	0.85	0.77	0.01
**$^2IM_{1-OH}$**	1.14	0.00	-0.04	-0.10	0.00	0.00	0.00
**$^4IM_{1-OH}$**	2.68	0.00	0.36	-0.05	0.00	0.00	0.00

Cartesian coordinates of all DFT-optimized structures

Cartesian coordinates of reaction species for TDCIPP C_1-hydroxylation and O-dealkylation

^{2}RC

S	-5.03139500	0.24766900	2.33892100
C	-4.20567800	3.39901500	0.14987600
C	-0.86143200	0.54687000	2.16182100
C	-3.27585200	-3.35108200	0.63142100
C	-6.46662200	-0.49497100	-1.61135600
C	-3.12285800	2.95917600	0.89233700
C	-2.18459400	3.82417200	1.56354000
C	-1.23659900	3.02293300	2.11838500
C	-1.59607700	1.66411700	1.79643300
C	-1.22636100	-0.77177900	1.90728300
C	-0.48466200	-1.92093500	2.36694600
C	-1.18597800	-3.01711300	1.97172400
C	-2.33771000	-2.53585500	1.24796700
C	-4.35064400	-2.90974700	-0.13251900
C	-5.29398100	-3.78183200	-0.78803400
C	-6.18454000	-2.97809800	-1.43144100
C	-5.78414500	-1.61940700	-1.15925100
C	-6.13261500	0.81941200	-1.32076300
C	-6.86969600	1.97074800	-1.77981200
C	-6.23423100	3.06554700	-1.28104500
C	-5.10968300	2.58134900	-0.51838800
N	-2.76344000	1.64043500	1.06609900
N	-2.34959800	-1.16386700	1.22706800
N	-4.66525300	-1.59927100	-0.37196800
N	-5.06563300	1.21185700	-0.54988300
Fe	-3.62598900	0.01983400	0.21470500
H	-4.36307000	4.47088100	0.08574500
H	0.07753500	0.70582000	2.67951500
H	-3.14574700	-4.42370300	0.73344700
H	-7.34220200	-0.66056300	-2.23110600
H	-0.36612600	3.30589400	2.69462700
H	-2.25408600	4.90346900	1.58713300
H	0.45514500	-1.86782700	2.89834300
H	-0.94950200	-4.06124900	2.12612300
H	-5.26408300	-4.86252700	-0.74940800
H	-7.04056300	-3.26091900	-2.02947100
H	-7.75682800	1.92784100	-2.39753600

H	-6.49025000	4.10938800	-1.40430400
O	-2.69279000	0.07901100	-1.12409600
H	-4.05457100	-0.02339900	3.22938100
P	2.99259000	-0.25746800	-0.13999500
O	2.23675000	-0.07161500	1.12255100
O	3.74608400	-1.65037400	-0.39285700
O	2.11117900	-0.16297900	-1.48116300
C	0.67030700	-0.10066000	-1.44643900
H	0.32627200	-0.12715100	-0.41107200
C	5.10423500	-1.96564200	-0.04724200
H	5.64766000	-1.05860000	0.22960500
C	0.09241700	-1.27931400	-2.22684400
H	-0.99644300	-1.21512100	-2.21277400
H	0.46909800	-1.29092200	-3.25099000
C	0.20280000	1.18429500	-2.12327100
H	0.58830500	1.25638800	-3.14130100
H	-0.88713600	1.21998100	-2.11112900
C	5.11700700	-2.97468500	1.10130700
H	4.44888000	-3.80902500	0.88206100
H	6.12960100	-3.34688100	1.26569800
C	5.75092600	-2.61467400	-1.27205500
H	6.75075700	-2.97490300	-1.02391400
Cl	4.57060500	-2.26085100	2.66672800
Cl	0.77665700	2.67577000	-1.26093200
Cl	0.53965400	-2.87388900	-1.49887700
H	5.13822900	-3.44537900	-1.62682700
Cl	5.93549600	-1.46764200	-2.64861700
O	4.19105500	0.80093700	-0.36332600
C	4.17016300	2.07011700	0.31292900
C	4.56973200	3.16337700	-0.67132300
C	4.89989100	1.98729600	1.65256600
H	3.13387300	2.31283500	0.58820000
H	4.55905800	4.12887100	-0.16071600
H	4.92205900	2.97341800	2.12123000
H	4.35882300	1.29252400	2.29522800
H	3.84014300	3.17955200	-1.48103100
Cl	6.60713000	1.39249000	1.59283900
Cl	6.18942600	2.99207600	-1.44917100

^{4}RC

S	-5.03570000	0.23167300	2.32327200
C	-4.21090200	3.40180600	0.15707800
C	-0.85838700	0.55283500	2.16236300
C	-3.27657300	-3.34611500	0.64218500

C	-6.45519700	-0.49439000	-1.62107400
C	-3.12829600	2.96187800	0.89953200
C	-2.19512000	3.82680800	1.57742200
C	-1.24410900	3.02665200	2.12861600
C	-1.59674000	1.66809700	1.79880700
C	-1.22219700	-0.76659300	1.90966600
C	-0.48180900	-1.91571600	2.37169700
C	-1.18637400	-3.01151100	1.98131100
C	-2.33807800	-2.53017800	1.25712500
C	-4.34739400	-2.90694400	-0.12913100
C	-5.28442400	-3.78091600	-0.79199400
C	-6.17040400	-2.97850100	-1.44328400
C	-5.77373800	-1.61909900	-1.16742800
C	-6.12481400	0.82004900	-1.32660000
C	-6.86557600	1.97000400	-1.78319600
C	-6.23621800	3.06590600	-1.27903100
C	-5.11113700	2.58381500	-0.51608900
N	-2.76194300	1.64334900	1.06418300
N	-2.34656100	-1.15794000	1.23188400
N	-4.66169600	-1.59777400	-0.37176200
N	-5.06016400	1.21494300	-0.55291300
Fe	-3.62342300	0.02779000	0.21949900
H	-4.37209300	4.47340800	0.09831500
H	0.07995700	0.71398700	2.68045800
H	-3.14752000	-4.41850200	0.74819600
H	-7.32809000	-0.65980900	-2.24468100
H	-0.37595600	3.31016200	2.70808800
H	-2.26985600	4.90558400	1.60767900
H	0.45881100	-1.86285600	2.90166800
H	-0.95197500	-4.05566400	2.13874500
H	-5.25326300	-4.86156800	-0.75305100
H	-7.02093600	-3.26251100	-2.04854800
H	-7.75139900	1.92542900	-2.40268500
H	-6.49678600	4.10900400	-1.39891700
O	-2.69750800	0.05224600	-1.12739100
H	-4.06202100	-0.02866300	3.22030500
P	2.99185900	-0.25958300	-0.13840700
O	2.23414700	-0.06910300	1.12229000
O	3.75103600	-1.65109100	-0.38188800
O	2.11106200	-0.17665700	-1.48086500
C	0.66992300	-0.12083400	-1.44763600
H	0.32510600	-0.14453100	-0.41247300
C	5.11061200	-1.95813900	-0.03432400
H	5.65030400	-1.04669400	0.23538200

C	0.09856400	-1.30554200	-2.22376300
H	-0.99056300	-1.24603900	-2.21302700
H	0.47794800	-1.32039000	-3.24686600
C	0.19698800	1.15916600	-2.13008900
H	0.58290000	1.22898800	-3.14812300
H	-0.89312400	1.18971300	-2.11923700
C	5.12790300	-2.95821600	1.12196400
H	4.46284500	-3.79677200	0.90959500
H	6.14201200	-3.32522900	1.28863000
C	5.75948700	-2.61372700	-1.25446500
H	6.76071300	-2.96841900	-1.00399700
Cl	4.57967800	-2.23431600	2.68212100
Cl	0.76261000	2.65686400	-1.27316100
Cl	0.55046000	-2.89478800	-1.48710400
H	5.14979300	-3.44924600	-1.60305800
Cl	5.93942200	-1.47601800	-2.63937600
O	4.18646000	0.80174800	-0.36762500
C	4.16045200	2.07586800	0.29906000
C	4.55415500	3.16352900	-0.69377500
C	4.89192200	2.00621400	1.63847500
H	3.12341700	2.31604100	0.57372400
H	4.54012100	4.13273900	-0.19034700
H	4.90959800	2.99575700	2.10007600
H	4.35490300	1.31341000	2.28665300
H	3.82341600	3.17062600	-1.50255800
Cl	6.60200700	1.41948300	1.58131400
Cl	6.17346500	2.99313100	-1.47259600

$^{2}TS_{1\text{-}H}$

S	-4.66613700	0.01053700	2.20776100
C	-3.17363200	3.40611000	0.75287100
C	-0.59343500	-0.08442500	2.88186100
C	-2.88974000	-3.37732700	0.19335800
C	-5.61108200	0.09700400	-1.77755700
C	-2.27595400	2.73817500	1.57351200
C	-1.41214500	3.38648200	2.53033100
C	-0.69902300	2.40552200	3.14622500
C	-1.11614300	1.15780600	2.55772000
C	-0.96677600	-1.29111000	2.29886700
C	-0.42692200	-2.57596300	2.66992800
C	-1.07311600	-3.50143300	1.90962500
C	-2.00530500	-2.77780800	1.07943600
C	-3.83968700	-2.71936400	-0.58260200
C	-4.75993900	-3.37637900	-1.47870900

C	-5.52941300	-2.39693700	-2.02951800
C	-5.07184800	-1.14936500	-1.46672300
C	-5.18837200	1.31492300	-1.26224100
C	-5.73861900	2.60090300	-1.62479600
C	-5.03947500	3.53364800	-0.92380800
C	-4.07168500	2.81319400	-0.12800800
N	-2.08613400	1.37583000	1.61040800
N	-1.91511700	-1.42909300	1.32137200
N	-4.04446900	-1.36870700	-0.59115600
N	-4.18066800	1.46836800	-0.34628800
Fe	-2.97644000	0.01672500	0.42974800
H	-3.19698100	4.48886900	0.82832900
H	0.18558400	-0.11760000	3.63520600
H	-2.84374200	-4.45839500	0.10573000
H	-6.42851800	0.11705700	-2.49196100
H	0.05009800	2.49942400	3.92087900
H	-1.37507000	4.45465700	2.69835100
H	0.34495200	-2.72583500	3.41256800
H	-0.95084500	-4.57647100	1.90394100
H	-4.80045800	-4.44381400	-1.65002100
H	-6.33413000	-2.49330200	-2.74631600
H	-6.55059100	2.75064000	-2.32403100
H	-5.15886500	4.60896900	-0.92606200
O	-1.95137000	0.30096000	-0.95714900
H	-3.83268400	-0.21629600	3.24438100
P	2.30364600	-0.08314400	-0.22878000
O	1.49476800	0.38779200	0.91410100
O	2.84367300	-1.59416000	-0.20731700
O	1.59451200	-0.06866500	-1.67031600
C	0.24005800	0.10803200	-2.02173700
H	-0.81687000	0.14230000	-1.05999400
C	4.12663900	-2.04530500	0.25010800
H	4.79712900	-1.19640200	0.40569700
C	-0.20746700	-1.02714300	-2.92393900
H	-1.26068300	-0.90069400	-3.17049400
H	0.39888000	-1.06773600	-3.83434600
C	0.02986200	1.44258700	-2.71154500
H	0.65693900	1.52957600	-3.60422100
H	-1.02209300	1.55933800	-2.96576700
C	3.95149200	-2.84045500	1.54412700
H	3.13352900	-3.55496500	1.44182000
H	4.87380900	-3.36429900	1.79976300
C	4.69704000	-2.97416800	-0.82342700
H	5.62238700	-3.43403100	-0.47239400

Cl	3.55439100	-1.79053900	2.96217300
Cl	0.46653400	2.86577800	-1.67265900
Cl	-0.03551800	-2.65334300	-2.15053500
H	3.97220400	-3.74961700	-1.07725300
Cl	5.09406300	-2.10062400	-2.34827400
O	3.66271300	0.75198300	-0.48048800
C	3.79983700	2.07589900	0.06687500
C	4.31258300	3.01758000	-1.01649400
C	4.53393400	2.03295400	1.40538200
H	2.80241200	2.46130300	0.32258400
H	4.43377900	4.01830500	-0.59598900
H	4.67803900	3.04873000	1.77935900
H	3.92547300	1.47054000	2.11405400
H	3.57387300	3.05555400	-1.81718500
Cl	6.15859700	1.23724000	1.39785900
Cl	5.88156500	2.57254800	-1.79022700

^{4}TS$_{1\text{-}H}$

S	-4.54167300	0.28500800	2.28810700
C	-2.51222700	3.39732700	0.80314200
C	-0.60704800	-0.55996700	2.81486000
C	-3.48688500	-3.30399000	0.06505600
C	-5.67297000	0.65645600	-1.62437200
C	-1.71423100	2.55708800	1.56920000
C	-0.71997400	3.00945700	2.51302600
C	-0.20740200	1.89592800	3.10491700
C	-0.87542800	0.76495500	2.50979600
C	-1.21803700	-1.66108200	2.22371800
C	-0.88874800	-3.03199000	2.52251900
C	-1.68933800	-3.80363000	1.73669300
C	-2.50871500	-2.89927700	0.96793500
C	-4.33006800	-2.46425700	-0.65096900
C	-5.38749600	-2.91928900	-1.52360400
C	-6.01707000	-1.80501700	-1.98564900
C	-5.33468300	-0.67474700	-1.40025200
C	-5.00892400	1.75484800	-1.09637000
C	-5.33722200	3.12866300	-1.38872600
C	-4.42964400	3.89731000	-0.72785100
C	-3.55563700	2.99459200	-0.01923000
N	-1.79186000	1.18813700	1.57898900
N	-2.20603100	-1.60050400	1.27800900
N	-4.31170500	-1.09595900	-0.59898400
N	-3.92393200	1.69291800	-0.25529500
Fe	-2.99599700	0.04605000	0.41539700

H	-2.33573300	4.46477400	0.89054700
H	0.16946000	-0.75662600	3.54548800
H	-3.61731700	-4.37261100	-0.07425700
H	-6.51538900	0.85301300	-2.28022400
H	0.54971400	1.82676600	3.87468800
H	-0.47715100	4.04716800	2.70058200
H	-0.13163500	-3.34287800	3.22951800
H	-1.73405400	-4.88258700	1.67158700
H	-5.61078900	-3.95703000	-1.73301600
H	-6.86387500	-1.73617300	-2.65554700
H	-6.15474100	3.44484300	-2.02296700
H	-4.34962200	4.97597800	-0.70443700
O	-1.93982500	0.00951600	-0.96671000
H	-3.84669000	-0.43767300	3.19034100
P	2.37507100	-0.35935000	-0.33359500
O	1.51155400	-0.33196800	0.86474400
O	3.24465700	-1.67149800	-0.64230800
O	1.62972500	-0.18750800	-1.74738100
C	0.25294300	-0.17879200	-2.05542900
H	-0.79694400	-0.07899700	-1.06831800
C	4.58719200	-1.92247300	-0.19585400
H	5.02799100	-1.01116600	0.21625200
C	-0.15082400	-1.45150200	-2.77450800
H	-1.20958800	-1.40948500	-3.02608500
H	0.45319500	-1.60275300	-3.67467600
C	-0.06205200	1.04026900	-2.89850100
H	0.54187200	1.06458000	-3.81064400
H	-1.12393900	1.05994700	-3.13663500
C	4.57457000	-3.03977800	0.84811900
H	3.97492800	-3.87971500	0.49417000
H	5.59200700	-3.37478800	1.05689000
C	5.39349000	-2.39959200	-1.40485100
H	6.39897000	-2.68950100	-1.09519000
Cl	3.87214700	-2.51858900	2.42706000
Cl	0.29956300	2.60158700	-2.04069500
Cl	0.09172400	-2.93595800	-1.76606100
H	4.89734400	-3.24770100	-1.88038800
Cl	5.58214700	-1.12438600	-2.66361400
O	3.51248600	0.78635200	-0.37003500
C	3.28574700	2.03645900	0.30660300
C	3.80710500	3.16980200	-0.56892500
C	3.74402700	1.96768200	1.76258300
H	2.20348700	2.22092400	0.37684300
H	3.63192400	4.12297500	-0.06528700

H	3.62905800	2.94745400	2.23088700
H	3.12002600	1.24026800	2.28099300
H	3.25872600	3.15664100	-1.51061600
Cl	5.45569300	1.44862000	2.03331300
Cl	5.55982100	3.11244200	-0.99886000

^{2}IM$_{1\text{-}H}$

S	-6.37037900	-0.90323600	0.65890000
C	-4.65596200	-1.32976700	-2.94423400
C	-5.40142400	2.87867700	-0.68472300
C	-3.52934400	0.97504700	3.34887800
C	-3.07928300	-3.31288200	1.17218600
C	-5.01729500	-0.01256900	-2.68467000
C	-5.59203900	0.88504900	-3.65910700
C	-5.82340400	2.06338100	-3.02058700
C	-5.38039900	1.89084000	-1.65810000
C	-4.94214400	2.73514200	0.62534000
C	-4.94227900	3.78332000	1.61582800
C	-4.40464500	3.24660100	2.74679300
C	-4.08391500	1.87372300	2.44019400
C	-3.22963600	-0.36137200	3.10807800
C	-2.69117100	-1.26671000	4.09769900
C	-2.57627800	-2.47962900	3.49152000
C	-3.03910300	-2.30373000	2.13434000
C	-3.48382600	-3.15286800	-0.14525100
C	-3.46668200	-4.19573500	-1.14390700
C	-3.88611400	-3.62925600	-2.30663900
C	-4.17310200	-2.24353200	-2.01496200
N	-4.89966800	0.61278400	-1.46939200
N	-4.41851400	1.58266200	1.14483000
N	-3.42693000	-1.01434600	1.92137600
N	-3.92336000	-1.97190000	-0.69402400
Fe	-4.11381900	-0.19147800	0.21146500
H	-4.79912400	-1.68816400	-3.95890100
H	-5.79460700	3.84969600	-0.96942400
H	-3.32134600	1.35274200	4.34539800
H	-2.74834200	-4.30172200	1.47434000
H	-6.24608200	2.97526800	-3.42076700
H	-5.78871500	0.62896100	-4.69158500
H	-5.30365000	4.78979700	1.45241500
H	-4.23561400	3.72202100	3.70373000
H	-2.44406700	-0.99508100	5.11529500
H	-2.21422200	-3.41050300	3.90704400
H	-3.16527500	-5.21876600	-0.96236000

H	-4.00387300	-4.09143800	-3.27767500
O	-2.41761400	0.29980200	-0.23907700
H	-6.96888000	0.30580800	0.63776800
P	3.70368000	-0.48910700	0.39191800
O	3.31048500	-0.43157700	1.81547700
O	4.48077000	-1.77892300	-0.14805100
O	2.46821200	-0.54681300	-0.66918800
C	1.14121800	-0.22996100	-0.49350800
H	-2.43875400	1.21610700	-0.55372600
C	5.90813000	-1.90764100	-0.30390100
H	6.38877000	-0.93409400	-0.18089100
C	0.62901000	0.42594000	0.70362300
H	-0.46031600	0.38132500	0.72127200
H	1.10128200	0.08888500	1.62209800
C	0.34400000	-0.41209500	-1.71283400
H	0.64093000	-1.30699300	-2.25874800
H	-0.72140000	-0.39774200	-1.48012500
C	6.43816100	-2.90418300	0.72625200
H	5.86278100	-3.83087100	0.69198300
H	7.49056500	-3.11864700	0.53274100
C	6.17495100	-2.45761900	-1.70495200
H	7.24048800	-2.65869900	-1.82945400
Cl	6.33417700	-2.28025300	2.41453800
Cl	0.59549800	0.97308900	-2.95036800
Cl	0.99700900	2.30992800	0.71909100
H	5.60925900	-3.37733700	-1.86610000
Cl	5.69987000	-1.30648900	-3.00412900
O	4.63390500	0.71577400	-0.11860200
C	4.66428100	1.98137400	0.57995600
C	4.62613800	3.10397400	-0.44897800
C	5.75914800	1.98819700	1.64299200
H	3.73038800	2.09858000	1.14657000
H	4.63873100	4.06450700	0.07037900
H	5.80940100	2.97477600	2.10808100
H	5.51144100	1.24468600	2.40087400
H	3.70038700	3.01920200	-1.01843000
Cl	7.42867100	1.58683300	1.07180400
Cl	5.96896900	3.13240300	-1.65499600

^{4}IM$_{1\text{-}H}$

S	-6.30826300	0.78969100	-0.68728500
C	-5.42641800	-1.51093700	2.47902700
C	-4.43111500	-2.63630700	-2.12238600
C	-2.90026100	1.89668800	-2.90098800

C	-3.89456400	3.02063900	1.70007200
C	-5.23577000	-2.22483100	1.30585600
C	-5.58673000	-3.61124700	1.12818300
C	-5.30599900	-3.92808400	-0.16573800
C	-4.78227400	-2.73651500	-0.78469300
C	-3.97825400	-1.48000000	-2.74040900
C	-3.57296100	-1.40609000	-4.12077300
C	-3.14315200	-0.13278800	-4.34032200
C	-3.28401800	0.57768200	-3.09513800
C	-2.99066400	2.57793800	-1.69733400
C	-2.62257200	3.96078500	-1.51526800
C	-2.90342200	4.27660100	-0.22190800
C	-3.44335100	3.08803600	0.39152400
C	-4.44479000	1.89316500	2.29193800
C	-4.87633500	1.82608800	3.66480900
C	-5.30636300	0.55290000	3.88313200
C	-5.13965900	-0.16415400	2.64471900
N	-4.72236500	-1.71857200	0.13601700
N	-3.81605200	-0.25361100	-2.13929900
N	-3.46915000	2.06212000	-0.51955600
N	-4.62865200	0.67590700	1.68480800
Fe	-4.13499700	0.19565200	-0.20351800
H	-5.83356600	-2.04466500	3.33089600
H	-4.51718600	-3.52911000	-2.73220000
H	-2.49845400	2.43286500	-3.75395400
H	-3.81337300	3.91613000	2.30664600
H	-5.44288500	-4.87334900	-0.67380000
H	-6.00183700	-4.24246200	1.90251100
H	-3.60558300	-2.23600300	-4.81402100
H	-2.75016000	0.29898200	-5.25113100
H	-2.21272100	4.59339800	-2.29135400
H	-2.77268100	5.22307700	0.28564900
H	-4.83713100	2.65339600	4.36079100
H	-5.69275100	0.11822900	4.79531300
O	-2.44956100	-0.27015400	0.26056400
H	-6.28142500	0.53144200	-2.01085700
P	3.72188300	0.48907100	-0.46860600
O	3.33975000	0.34526200	-1.88885400
O	4.53933000	1.78621800	-0.01626700
O	2.47794900	0.66894200	0.57082600
C	1.14958500	0.35037800	0.42067100
H	-2.44407500	-1.20386400	0.52708100
C	5.96209700	1.86618300	0.20184300
H	6.40020800	0.86531500	0.18783600

C	0.62063800	-0.33034300	-0.75494800
H	-0.46529100	-0.25523900	-0.78337800
H	1.10643900	-0.04112800	-1.68250400
C	0.37258700	0.56232100	1.64910200
H	0.68144700	1.46863900	2.16885200
H	-0.69668400	0.54848800	1.44050100
C	6.58595600	2.74398100	-0.88231600
H	6.05835800	3.69667700	-0.95254300
H	7.63898000	2.92329300	-0.65883400
C	6.18767400	2.52626200	1.56200200
H	7.25407800	2.69380700	1.72313700
Cl	6.52095100	1.98396800	-2.51615900
Cl	0.64345000	-0.79561400	2.90915500
Cl	0.92048100	-2.22587600	-0.70324000
H	5.65668400	3.47858600	1.61448500
Cl	5.60006000	1.51374300	2.92923700
O	4.59663000	-0.71092600	0.14038400
C	4.60853400	-2.01589000	-0.48331500
C	4.46941000	-3.07528400	0.60230800
C	5.75580800	-2.12404300	-1.48386400
H	3.70111700	-2.12672900	-1.09252200
H	4.45752700	-4.06300600	0.13666500
H	5.79038700	-3.13629900	-1.89160800
H	5.57802200	-1.41546200	-2.29326100
H	3.52429700	-2.91498100	1.12158500
Cl	7.40786900	-1.75291800	-0.84604100
Cl	5.75373000	-3.09933600	1.87030600

$^4TS_{1\text{-}OH}$

S	-4.57292500	-1.35401900	1.34821500
C	-1.43516100	-3.67139200	0.33158100
C	-3.85569000	-0.70326900	-2.64251200
C	-3.87044100	2.71265600	0.78426800
C	-1.20646500	-0.15100900	3.65043600
C	-2.15545700	-3.17727000	-0.74772400
C	-2.42062800	-3.91759800	-1.95529800
C	-3.08709200	-3.07759900	-2.79537100
C	-3.23888700	-1.82319100	-2.10294800
C	-4.02504100	0.50579200	-1.98362300
C	-4.74178500	1.63646400	-2.52453200
C	-4.75993200	2.59119200	-1.55493900
C	-4.04738400	2.04918800	-0.42198600
C	-3.18451200	2.20443600	1.87956500
C	-2.93830700	2.94065700	3.09390000

C	-2.17613400	2.14323400	3.89303400
C	-1.96041600	0.91310100	3.17374000
C	-0.99205600	-1.34065100	2.96981000
C	-0.29500400	-2.47748000	3.51617200
C	-0.37252800	-3.47316800	2.58976400
C	-1.11009100	-2.94622100	1.46844400
N	-2.67290900	-1.90912000	-0.85400000
N	-3.59157600	0.78945900	-0.71442300
N	-2.59749000	0.96422700	1.95750500
N	-1.45347500	-1.63672000	1.70890200
Fe	-2.56859400	-0.44916800	0.51685800
H	-1.08811100	-4.69663900	0.27347400
H	-4.25651300	-0.78859300	-3.64684200
H	-4.28533200	3.71099800	0.87364300
H	-0.77129100	-0.05366800	4.63921300
H	-3.44561600	-3.27231300	-3.79738000
H	-2.11353900	-4.94026800	-2.12686400
H	-5.17858700	1.67122200	-3.51370700
H	-5.21194800	3.57373300	-1.58392900
H	-3.29472200	3.94385800	3.28633100
H	-1.78120100	2.35624100	4.87745900
H	0.17271400	-2.49957400	4.49141400
H	0.01667100	-4.48089400	2.64944500
O	-0.99387900	0.25789000	-0.21949800
H	-4.75195600	-0.49129700	2.36898100
P	2.66060500	0.43804500	-1.18717400
O	2.75975200	0.03195400	-2.60134200
O	4.03038600	1.00789100	-0.60820900
O	2.12030900	-0.70302700	-0.17167300
O	1.67421700	1.67027200	-0.81388200
C	0.39020400	1.93422900	-1.22613000
H	-0.24852200	-0.15280400	0.25082100
C	4.45126500	1.25929300	0.75107500
H	4.35031200	0.33742700	1.32858000
C	-0.26265700	2.94820000	-0.38066400
H	-1.30769800	3.06895400	-0.65114900
H	-0.16447800	2.69958500	0.67312300
C	-0.04222700	1.59105200	-2.59099200
H	0.37201100	0.64648200	-2.93360700
H	-1.12763700	1.58293000	-2.64765300
C	5.92329300	1.65775400	0.65500300
H	6.04215800	2.53850300	0.02107800
H	6.31193100	1.86627000	1.65268800
C	3.66476600	2.40239200	1.41336900

H	4.30566300	2.96168500	2.09638900
Cl	6.95501100	0.35643200	-0.04562700
Cl	0.52432900	2.83421300	-3.85702300
Cl	0.51863900	4.63077900	-0.53807900
H	3.25276400	3.07912300	0.66484300
Cl	2.28196000	1.80964700	2.42272300
C	2.32120300	-2.13200100	-0.38338400
C	3.79417000	-2.52212700	-0.33545200
C	1.62859600	-2.57273600	-1.68268500
H	1.79884800	-2.58674000	0.46068200
H	4.41140200	-1.88340400	-0.96957600
H	3.90714900	-3.55880300	-0.65016300
H	0.60190200	-2.20639100	-1.68568900
H	2.15148300	-2.22491100	-2.57052700
Cl	4.46088300	-2.42602900	1.34613000
Cl	1.54632900	-4.38310200	-1.76658000

$^{2}IM_{1\text{-}OH}$

S	-3.86617900	-0.98931200	2.55333500
C	-4.43318400	-1.22765900	-1.51255600
C	-3.29214900	2.82208100	0.87606900
C	-0.12051300	0.31549600	3.52856600
C	-0.99899100	-3.64886000	0.89062600
C	-4.41181400	0.08696200	-1.07101800
C	-5.26010500	1.13024900	-1.59306200
C	-4.92424000	2.27464400	-0.93819400
C	-3.87734400	1.92977400	-0.00946400
C	-2.32774600	2.48404400	1.81349300
C	-1.75785900	3.40745700	2.76416300
C	-0.88318600	2.69527900	3.52398500
C	-0.91156800	1.33925700	3.03034300
C	-0.07429400	-0.97674200	3.02610000
C	0.79065700	-2.01214100	3.53100400
C	0.56081500	-3.12384700	2.77588100
C	-0.45158600	-2.77070100	1.81493500
C	-2.01924000	-3.32942600	0.00697200
C	-2.61185400	-4.25806800	-0.92183500
C	-3.59014200	-3.58095400	-1.58330700
C	-3.59115100	-2.23563200	-1.06442500
N	-3.56405500	0.59249000	-0.11074000
N	-1.79556600	1.22739700	1.98284500
N	-0.81868400	-1.44871900	1.96552600
N	-2.62004400	-2.09239200	-0.09805500
Fe	-2.31371500	-0.46837500	1.04250600

H	-5.15266800	-1.48424000	-2.28334500
H	-3.63078400	3.85258100	0.85007500
H	0.53296500	0.55041100	4.36206200
H	-0.61472000	-4.66316500	0.87232300
H	-5.33870200	3.26685800	-1.05755900
H	-6.00631200	0.98834800	-2.36361000
H	-2.00866000	4.45797300	2.82753600
H	-0.26061800	3.03894500	4.33922600
H	1.47839400	-1.89018600	4.35749800
H	1.01913300	-4.10057300	2.85585600
H	-2.30732400	-5.28951000	-1.03975400
H	-4.25378200	-3.94208400	-2.35777600
O	-0.17711500	0.15003800	-0.55386500
H	-3.61478300	-2.31406000	2.62797300
P	2.87728300	0.43947500	-1.49365900
O	3.95342400	-0.01304000	-2.39345400
O	3.27805100	1.82110400	-0.75760100
O	2.49733200	-0.61401100	-0.30931600
O	1.44350600	0.78201100	-2.12038400
C	0.13873300	0.18928000	-1.91897300
H	0.37663800	-0.51012300	-0.10110600
C	2.32683100	2.75794100	-0.20154200
H	1.40258500	2.24650000	0.08017600
C	-0.89241500	1.18247400	-2.48938600
H	-1.85225600	0.67705000	-2.58926200
H	-0.99475600	1.99863800	-1.77506900
C	0.07529400	-1.24049000	-2.47339500
H	0.75403400	-1.87964800	-1.90778900
H	-0.94365900	-1.60635200	-2.34771500
C	2.97187300	3.40462700	1.01467700
H	3.97864300	3.74894300	0.77289100
H	2.36311900	4.23996300	1.35814300
C	2.04346300	3.79337500	-1.29930100
H	2.93208200	4.38725000	-1.51965100
Cl	3.12627400	2.26866200	2.41525300
Cl	0.51812000	-1.42159700	-4.20658100
Cl	-0.48310900	1.91767900	-4.07586800
H	1.70327400	3.29143800	-2.20363300
Cl	0.72989100	4.93709800	-0.80290400
C	3.47125600	-1.53396400	0.27529300
C	4.71235800	-0.75908800	0.73900600
C	3.83198600	-2.66525600	-0.68011100
H	2.95169300	-1.92999200	1.15009600
H	4.40850900	0.13243800	1.28514000

H	5.35501000	-0.48408900	-0.09802600
H	4.14099800	-2.27413600	-1.64900400
H	4.62667600	-3.26871600	-0.24216800
Cl	5.70080500	-1.76749300	1.87252600
Cl	2.43596400	-3.78651200	-0.96621900

^{4}IM$_{1\text{-}OH}$

S	-5.66359300	1.10035200	-0.23539600
C	-2.19061200	3.22311000	-1.60138000
C	-2.74386900	1.73194700	2.96822400
C	-4.01946100	-2.66479300	1.37264200
C	-3.56382600	-1.13707100	-3.19500800
C	-2.18592000	3.17782700	-0.21599200
C	-1.82220700	4.28606800	0.62990000
C	-1.98413400	3.86900600	1.91724200
C	-2.44493600	2.50562800	1.85728100
C	-3.17325100	0.41373300	2.92090100
C	-3.42817500	-0.39433400	4.08244100
C	-3.78161200	-1.63241300	3.63435200
C	-3.74668700	-1.58235100	2.19784100
C	-3.96931300	-2.63442900	-0.01253000
C	-4.27071400	-3.76013300	-0.86049400
C	-4.14947100	-3.32770100	-2.14534300
C	-3.77027000	-1.93877500	-2.08384300
C	-3.18284800	0.19557000	-3.14813800
C	-2.91549100	0.99908600	-4.31223800
C	-2.52389500	2.22347400	-3.86386300
C	-2.55404200	2.16954000	-2.42569400
N	-2.54729000	2.09182600	0.54813300
N	-3.37920600	-0.32264000	1.77159400
N	-3.64854100	-1.53302900	-0.77383500
N	-2.96664800	0.92662000	-2.00229300
Fe	-3.36308200	0.35987300	-0.12041300
H	-1.88490300	4.14922900	-2.07639200
H	-2.62029200	2.18498000	3.94604400
H	-4.29330800	-3.60132600	1.84678700
H	-3.69619700	-1.58765400	-4.17281500
H	-1.82111500	4.42273400	2.83237200
H	-1.50563900	5.25624700	0.26976600
H	-3.33532500	-0.04933000	5.10368300
H	-4.03785100	-2.51011000	4.21277000
H	-4.54281000	-4.74426400	-0.50280600
H	-4.30047800	-3.88357000	-3.06109600
H	-3.00771400	0.65247400	-5.33281000

H	-2.22857100	3.08981000	-4.44049000
O	-0.33798100	-0.15033400	-0.11574500
H	-6.01397100	0.60751400	0.97078800
P	2.62192200	0.05857300	-0.88523300
O	2.26945400	-0.17088700	-2.29876700
O	2.38257800	1.57678500	-0.41428400
O	1.85148700	-0.89758400	0.18791300
C	0.47525500	-1.27906400	0.07181200
H	-0.22863900	0.45618500	0.63522600
C	3.12223800	2.31525600	0.57194300
H	3.86920700	1.67485600	1.04752800
C	0.18438900	-2.12202400	-1.18362200
H	-0.84781400	-2.46792100	-1.14457000
H	0.34285700	-1.49221700	-2.05637700
C	0.07993100	-1.90980000	1.42019000
H	0.10170500	-1.12883500	2.18058800
H	-0.93119200	-2.30731700	1.34000600
C	3.79929000	3.50357000	-0.11863200
H	3.06049000	4.08340800	-0.67503800
H	4.28253000	4.14212500	0.62327600
C	2.13988000	2.86956000	1.60255700
H	2.65451800	3.52915500	2.30252000
Cl	5.07030700	3.00365400	-1.28785300
Cl	1.13637300	-3.23357600	2.04352500
Cl	1.23835400	-3.57006500	-1.39037900
H	1.32549900	3.40900600	1.11602900
Cl	1.36644800	1.57818600	2.60916000
O	4.13132600	-0.20643600	-0.41137900
C	4.67326300	-1.54411600	-0.44519200
C	4.90591100	-2.04184800	0.98073500
C	5.82301000	-1.60827900	-1.44341500
H	3.91997500	-2.23293800	-0.85619100
H	5.45350900	-2.98615500	0.95741900
H	6.17982100	-2.63833800	-1.51097300
H	5.44948200	-1.29407400	-2.41870200
H	3.93878100	-2.19672300	1.45821600
Cl	7.25877200	-0.57580200	-1.08531900
Cl	5.82201300	-0.91811200	2.06131700

IM$_{1\text{-}OH}$

O	0.83248500	-2.58750100	1.16841000
P	-0.15775600	0.24687500	0.82754300
O	-0.45770100	0.10266300	2.27578500
O	0.09479500	1.79092700	0.44550200

O	-1.36473300	-0.24674600	-0.12118400
O	1.13004000	-0.44907600	0.20594000
C	1.42786900	-1.88735300	0.14626200
H	1.17096000	-2.28080200	2.05416700
C	1.11545300	2.32769100	-0.41338500
H	1.66469800	1.51627500	-0.89487400
C	0.82162200	-2.50055600	-1.12635100
H	1.22933700	-3.50332400	-1.25660800
H	-0.25454100	-2.56647900	-0.97785700
C	2.95292200	-1.99749100	0.31420100
H	3.20242700	-1.64660300	1.31582800
H	3.22974800	-3.04761600	0.21964900
C	0.45115900	3.22553500	-1.45658900
H	-0.18916600	3.96478900	-0.97218600
H	1.20948600	3.73592100	-2.05255000
C	2.05777400	3.18964100	0.42884500
H	2.79831100	3.67355300	-0.20977600
Cl	-0.58349400	2.30544800	-2.61276100
Cl	3.98674500	-1.06233000	-0.82946300
Cl	1.09564600	-1.59545600	-2.65697300
H	1.49526900	3.94561800	0.97905700
Cl	2.98055900	2.23804700	1.65538900
O	1.37105900	-1.47407000	3.52980900
H	0.65559400	-0.83132500	3.32607600
H	2.14681900	-0.93202100	3.71995700
C	-2.73435600	-0.25775900	0.34891700
C	-3.49795300	-1.23195300	-0.53307700
C	-3.27229500	1.17382300	0.22320300
H	-2.75581700	-0.57546100	1.39449600
H	-4.56475700	-1.16752500	-0.32372800
H	-3.28932300	1.49830600	-0.81799400
Cl	-3.00753100	-2.94754400	-0.24344000
Cl	-4.95429400	1.31683400	0.87784000
H	-3.31074300	-1.01716200	-1.58642700
H	-2.65244200	1.85672600	0.80331800

TS_{BDCIPP}

O	1.42300800	-2.54958500	1.40440800
P	-0.18778400	0.23186900	0.65268300
O	-0.51343600	0.05803800	2.12839700
O	-0.16581600	1.82626200	0.33412000
O	-1.41721800	-0.32742800	-0.23244800
O	1.09870200	-0.36016100	0.08535400
C	1.80742300	-2.07253500	0.28848100

H	1.45314400	-1.82836300	2.50079400
C	0.93161700	2.49108700	-0.30475700
H	1.62607000	1.75371100	-0.71471700
C	1.14197600	-2.80508600	-0.87296900
H	1.54856300	-3.81993700	-0.86935900
H	0.07692100	-2.85408900	-0.65253800
C	3.27932800	-1.65229500	0.28894200
H	3.45934000	-1.11437300	1.21877400
H	3.85986100	-2.57862900	0.30144300
C	0.37604900	3.37494300	-1.42156400
H	-0.38986900	4.05029900	-1.03585000
H	1.17717100	3.95642900	-1.88063100
C	1.64015100	3.38449600	0.71424500
H	2.39563100	4.00251200	0.22694500
Cl	-0.38793100	2.40815300	-2.73921000
Cl	3.95101400	-0.63753000	-1.03532100
Cl	1.32686400	-2.17162500	-2.54423000
H	0.92166000	4.01799800	1.23613400
Cl	2.51496400	2.44432000	1.99453800
O	1.19932000	-1.14527600	3.34466400
H	0.38570300	-0.55484300	2.87832400
H	1.93166800	-0.54327500	3.53688400
C	-2.77823000	-0.33971600	0.24068000
C	-3.46257000	-1.54320300	-0.38832300
C	-3.41607300	0.98312000	-0.20286500
H	-2.78918300	-0.41744400	1.33115600
H	-4.53046300	-1.52544100	-0.17510600
H	-3.46115400	1.05492200	-1.29040200
Cl	-2.82033300	-3.10444900	0.26285700
Cl	-5.10391800	1.16217500	0.43257100
H	-3.29620000	-1.55455300	-1.46669600
H	-2.83781800	1.81962300	0.18694300

P_{BDCIPP}

O	2.53592600	0.53434800	-0.99828600
P	-1.76369900	0.75789400	-0.66393000
O	-1.11362300	1.89622200	0.04430400
O	-3.35791200	0.80916600	-0.85824700
O	-1.60799200	-0.66183700	0.10918900
O	-1.24721700	0.54075500	-2.14234300
C	3.71125000	0.54125700	-0.67503600
H	1.40207900	1.82436300	-1.82126100
C	-4.35791500	0.25682300	0.00852600
H	-3.89539300	-0.39562900	0.75271800

C	4.29074800	-0.63025300	0.09954800
H	4.59693600	-0.31244200	1.09762100
H	3.54459800	-1.41993500	0.16419400
C	4.59999000	1.72076100	-1.04571300
H	5.40904600	1.40040700	-1.70401300
H	3.99368100	2.48663800	-1.52721900
C	-5.12190800	1.38837300	0.69673300
H	-5.39998500	2.15369900	-0.02926000
H	-6.01838900	1.00184200	1.18444600
C	-5.32924200	-0.54692600	-0.85658200
H	-6.18748200	-0.87103900	-0.26601000
Cl	-4.14396600	2.19721100	1.97899300
Cl	5.36149500	2.44525400	0.42514700
Cl	5.75803800	-1.29117500	-0.72994700
H	-5.66970700	0.05271700	-1.70216400
Cl	-4.57683300	-2.03971300	-1.53757200
O	0.64114600	2.34779100	-2.13282000
H	0.21808500	2.65434500	-1.30748400
H	-0.51174500	1.20881200	-2.33716900
C	-0.28911400	-1.08799000	0.54634600
C	-0.33560800	-2.60889000	0.37083500
C	-0.14548600	-0.55443900	1.97400800
H	0.48538500	-0.66447300	-0.10043100
H	-0.90100500	-3.08634600	1.17277800
H	-0.85040900	-1.05007300	2.64360300
Cl	1.29609500	-3.39093400	0.32866200
Cl	1.50785200	-0.78209500	2.68036900
H	-0.80889600	-2.82831600	-0.58517500
H	-0.33582400	0.51786800	1.96133100

Cartesian coordinates of reaction species for TCIPP C_1-hydroxylation and O-dealkylation

^{2}RC

S	-4.43728800	0.81199000	2.27874800
C	-3.73514900	3.32907400	-0.66986200
C	-0.30244200	0.96184200	1.77998300
C	-2.77316000	-3.16071800	1.25108800
C	-6.18912800	-0.79846300	-1.22214500
C	-2.60499700	3.05096900	0.08244500
C	-1.61196500	4.02639100	0.45887900
C	-0.64575900	3.36062900	1.14778700
C	-1.04599400	1.97605500	1.19680700
C	-0.68000500	-0.37637900	1.84036000

C	0.10565800	-1.40893500	2.47150600
C	-0.59620800	-2.56639000	2.33824500
C	-1.80060600	-2.23984700	1.61386200
C	-3.92063200	-2.87507800	0.52079300
C	-4.91873800	-3.85258300	0.16052800
C	-5.87930500	-3.18798400	-0.53756800
C	-5.46281600	-1.80792700	-0.59965200
C	-5.82065200	0.53725900	-1.28385200
C	-6.58676200	1.56735300	-1.94213000
C	-5.89157600	2.72684900	-1.79217100
C	-4.70438400	2.40469200	-1.03794800
N	-2.25465700	1.80697800	0.55785300
N	-1.83689700	-0.89927300	1.32559200
N	-4.27013100	-1.63850200	0.04887900
N	-4.68003400	1.06788100	-0.73418800
Fe	-3.18699200	0.04978000	0.17156700
H	-3.88202100	4.35677200	-0.98634000
H	0.66825100	1.21700700	2.18967500
H	-2.61972500	-4.19168100	1.55348700
H	-7.12270500	-1.07987100	-1.69890400
H	0.25961400	3.75231400	1.59188700
H	-1.66426300	5.07984100	0.21782100
H	1.07070800	-1.24448900	2.92956300
H	-0.33342500	-3.55914700	2.67801000
H	-4.87490700	-4.90220900	0.41870200
H	-6.79015100	-3.57725500	-0.97257000
H	-7.53027400	1.40609600	-2.44629800
H	-6.14560300	3.71698200	-2.14642900
O	-2.36019700	-0.21253300	-1.20827200
H	-3.39325600	0.77116500	3.13293600
P	3.57002300	0.03303800	-0.27040700
O	2.85688100	0.43555100	0.97458200
O	4.33470600	-1.37065300	-0.26844800
O	2.66183700	-0.08143100	-1.58053800
C	1.20467300	-0.15159900	-1.51830800
H	0.90202500	-0.20939700	-0.47193300
C	5.44629500	-1.62829600	0.62876600
H	6.04243800	-0.71516100	0.72634500
C	0.73888800	-1.39312300	-2.25997800
H	-0.35171800	-1.40365400	-2.27896700
H	1.14168000	-1.41852000	-3.27372200
C	0.63689400	1.10723600	-2.16374800
H	0.98389800	1.20392700	-3.19751600
H	-0.45552700	1.06632400	-2.14800900

C	4.92148500	-2.08028200	1.99061500
H	4.33736500	-3.00043800	1.89235500
H	5.75205800	-2.25969000	2.68050900
C	6.28286100	-2.71678000	-0.02597300
H	7.05666900	-3.05872900	0.66303600
Cl	1.26326300	-2.93213300	-1.45506400
H	5.64923800	-3.55712700	-0.31424500
Cl	7.12910600	-2.15567900	-1.52299700
O	4.76384500	1.02419800	-0.71003300
C	4.62648200	2.45885400	-0.59384400
C	5.54049600	3.07413500	-1.64172700
C	4.88792400	2.91455600	0.84056400
H	3.58417100	2.73826500	-0.80579400
H	5.47254900	4.16620700	-1.61079400
H	4.74977100	3.99488600	0.91823600
H	4.21283600	2.39944800	1.52256700
H	5.24835300	2.73079400	-2.63666900
Cl	6.57440800	2.56418300	1.41435700
H	6.57703700	2.78167500	-1.46074800
H	4.27786100	-1.30474200	2.41117500
H	0.95708100	1.99074900	-1.60419600

^{4}RC

S	-4.44626900	0.79247700	2.26686200
C	-3.74013200	3.33278100	-0.66680100
C	-0.30236900	0.96990300	1.78238700
C	-2.77267300	-3.15325100	1.26182200
C	-6.17801700	-0.80231900	-1.23478000
C	-2.61156200	3.05558200	0.08805400
C	-1.62518500	4.03376800	0.47375200
C	-0.65722200	3.36892600	1.16102100
C	-1.04969000	1.98205200	1.20068500
C	-0.67716100	-0.36911800	1.84357000
C	0.10747600	-1.40060200	2.47788300
C	-0.59644800	-2.55724200	2.34884700
C	-1.80036400	-2.23151700	1.62290100
C	-3.91658600	-2.87194100	0.52387500
C	-4.90846700	-3.85350400	0.15602600
C	-5.86519200	-3.19268400	-0.55077000
C	-5.45269700	-1.81093000	-0.60933600
C	-5.81296500	0.53444400	-1.29308400
C	-6.58191100	1.56317000	-1.95015100
C	-5.89220600	2.72537100	-1.79575800
C	-4.70516700	2.40618800	-1.04034000

N	-2.25502400	1.80974900	0.55509700
N	-1.83403000	-0.89169500	1.32966500
N	-4.26651900	-1.63750000	0.04851100
N	-4.67473000	1.06870800	-0.74069900
Fe	-3.18409100	0.05811000	0.17330500
H	-3.89020500	4.36139900	-0.97872300
H	0.66662700	1.22850300	2.19389000
H	-2.61976900	-4.18299300	1.56870100
H	-7.10876500	-1.08515500	-1.71623300
H	0.24454300	3.76282300	1.61045900
H	-1.68284400	5.08845900	0.23950000
H	1.07273300	-1.23610500	2.93548400
H	-0.33522400	-3.54924100	2.69201600
H	-4.86287900	-4.90292100	0.41474300
H	-6.77077500	-3.58532000	-0.99366200
H	-7.52393200	1.39924000	-2.45624600
H	-6.15002600	3.71536900	-2.14761500
O	-2.36414700	-0.24335900	-1.20472200
H	-3.40398100	0.77410200	3.12382100
P	3.56913700	0.03334800	-0.27016800
O	2.85587200	0.44171400	0.97281700
O	4.33895200	-1.36743700	-0.25972000
O	2.65926400	-0.09350800	-1.57810400
C	1.20263300	-0.17014900	-1.51223800
H	0.90270900	-0.22444900	-0.46488600
C	5.45550300	-1.61424600	0.63444700
H	6.05181700	-0.69981100	0.71763900
C	0.74115800	-1.41738000	-2.24704800
H	-0.34939400	-1.43261000	-2.26487000
H	1.14306000	-1.44632100	-3.26104500
C	0.62761800	1.08315600	-2.16211600
H	0.97213000	1.17689300	-3.19698400
H	-0.46463200	1.03744700	-2.14443500
C	4.93891500	-2.05017900	2.00466600
H	4.35583800	-2.97245400	1.92118600
H	5.77371800	-2.21971700	2.69193800
C	6.28852800	-2.71025400	-0.01214900
H	7.06637400	-3.04387200	0.67639300
Cl	1.27276900	-2.94997900	-1.43469000
H	5.65338700	-3.55402400	-0.28675400
Cl	7.12605000	-2.16674600	-1.52054700
O	4.75848200	1.02602200	-0.71837000
C	4.61650300	2.46091300	-0.61066100
C	5.52636300	3.07292600	-1.66405700

C	4.87916800	2.92608100	0.82047700
H	3.57286400	2.73554700	-0.82229300
H	5.45503900	4.16493800	-1.63929500
H	4.73739100	4.00636300	0.89201300
H	4.20718600	2.41270500	1.50685000
H	5.23318200	2.72293100	-2.65637300
Cl	6.56798900	2.58500700	1.39305600
H	6.56421400	2.78480900	-1.48366000
H	4.29635600	-1.27062500	2.41938900
H	0.94477600	1.97058100	-1.60704100

$^{2}TS_{1\text{-}H}$

S	-4.17366400	0.03708400	2.19562700
C	-0.76133900	2.31266500	2.31216100
C	-0.95880100	-2.50901900	1.91507200
C	-4.52909400	-2.05337800	-1.31267800
C	-4.31479100	2.76075400	-0.93372400
C	-0.51116400	0.95936100	2.47779100
C	0.43985200	0.42710800	3.42081600
C	0.38882900	-0.92818000	3.31014100
C	-0.59266400	-1.22611200	2.29740700
C	-1.94787700	-2.80700100	0.98953800
C	-2.34782000	-4.14193600	0.61597300
C	-3.35266800	-4.00754500	-0.29045700
C	-3.56795000	-2.59094800	-0.46818000
C	-4.78852200	-0.70050700	-1.49080400
C	-5.78665400	-0.16900700	-2.39046200
C	-5.72556200	1.18598200	-2.28557200
C	-4.68996300	1.48152200	-1.32227400
C	-3.31733900	3.06053000	-0.01468100
C	-2.95056700	4.39939600	0.38405900
C	-1.95135300	4.27481300	1.29944600
C	-1.70934800	2.85993800	1.45705200
N	-1.12654600	-0.06402200	1.79400000
N	-2.69827400	-1.87845400	0.31778300
N	-4.14001600	0.31881900	-0.84815500
N	-2.54927000	2.14204800	0.65206600
Fe	-2.57650300	0.12256000	0.44087800
H	-0.17117000	3.00112000	2.90799000
H	-0.44583800	-3.33967900	2.38853500
H	-5.13464800	-2.75322900	-1.87995200
H	-4.84457300	3.59446100	-1.38387900
H	0.94355500	-1.67430000	3.86377200
H	1.05228600	1.02953300	4.07820500

H	-1.90646300	-5.04904000	1.00689300
H	-3.90984200	-4.78227200	-0.80033600
H	-6.43871800	-0.76943800	-3.01108000
H	-6.31686800	1.93059900	-2.80220500
H	-3.41094200	5.30255900	0.00538900
H	-1.41877200	5.05386200	1.82879600
O	-1.62054100	0.29250800	-1.01449700
H	-5.10721000	0.84677800	1.65680200
P	2.77182600	0.27082900	-0.29744300
O	2.26187600	1.47611400	0.39798500
O	3.31614600	-0.96341600	0.54673800
O	1.69289900	-0.58370500	-1.16143200
C	0.61653200	-0.11226700	-1.90246300
H	-0.55067100	0.00530900	-1.08540100
C	4.55609400	-0.90334400	1.30518500
H	5.29103200	-0.32730800	0.73544900
C	0.18986000	-1.14529000	-2.91713500
H	-0.75131800	-0.83108600	-3.36590400
H	0.96012600	-1.24447200	-3.69120200
C	0.70641400	1.29466000	-2.44362600
H	1.53005300	1.37689200	-3.16914600
H	-0.22934600	1.55222700	-2.94112000
C	4.30054100	-0.26106600	2.66640300
H	3.57300100	-0.84375400	3.23830800
H	5.23258500	-0.19215700	3.23551700
C	5.03602400	-2.33875400	1.45100400
H	5.89325900	-2.37579600	2.12493200
Cl	-0.05708600	-2.79904100	-2.24488800
H	4.23541000	-2.96961500	1.84067000
O	3.98827000	0.57393900	-1.31737100
C	4.30674700	1.91064100	-1.78620500
C	5.11173400	1.73604800	-3.06433700
C	4.99155800	2.74180300	-0.70486900
H	3.37164500	2.44268900	-2.00156500
H	5.37301400	2.71330600	-3.48184100
H	5.17572600	3.74895300	-1.08350900
H	4.36416300	2.79463800	0.18362700
H	4.52703000	1.18451300	-3.80452900
H	0.86967800	2.00721600	-1.63230000
H	6.03149700	1.18229800	-2.86477800
H	3.89665000	0.74514700	2.53442400
Cl	5.56962200	-3.06607700	-0.11552600
Cl	6.60224300	2.08513300	-0.18255800

$^{4}TS_{1-H}$

S	3.99447300	0.11820600	2.41452800
C	0.11303600	-0.26353200	2.46359100
C	2.44661000	3.38981800	0.32071000
C	5.61562500	0.28004100	-1.58684700
C	3.13237100	-3.39534800	0.34881900
C	0.49962900	1.01320400	2.07632000
C	-0.16877300	2.22602700	2.48124700
C	0.47853100	3.25330600	1.86781600
C	1.54391500	2.66593100	1.09118700
C	3.50354900	2.85575100	-0.40430300
C	4.45637300	3.64419000	-1.15121200
C	5.35949700	2.77283600	-1.67456100
C	4.95206300	1.45305800	-1.25245900
C	5.21535300	-0.99779400	-1.21946000
C	5.88682300	-2.21052800	-1.61858900
C	5.17595800	-3.24369800	-1.09015600
C	4.07518100	-2.66162900	-0.36064200
C	2.09405200	-2.86500000	1.10558800
C	1.14671400	-3.64470500	1.86624200
C	0.30075200	-2.76124000	2.46443500
C	0.73249500	-1.44182700	2.07296000
N	1.54324200	1.30494000	1.23592900
N	3.82093400	1.52552200	-0.48513300
N	4.11752300	-1.29157200	-0.45097600
N	1.82658400	-1.52676600	1.24444800
Fe	2.79139100	0.00037700	0.30659900
H	-0.75925100	-0.35222200	3.10102400
H	2.32354200	4.46788900	0.29612600
H	6.50596800	0.36700300	-2.20143900
H	3.22961100	-4.47643000	0.32862300
H	0.26857500	4.31320500	1.92202300
H	-1.02194700	2.26441800	3.14485400
H	4.42407200	4.72151100	-1.24544900
H	6.22368500	2.98373900	-2.29034800
H	6.78077400	-2.24771600	-2.22693000
H	5.36722000	-4.30559200	-1.17193900
H	1.14343800	-4.72534100	1.92306800
H	-0.55060500	-2.96195300	3.09983900
O	1.79350900	-0.10156400	-1.10637900
H	4.49282800	-1.13573700	2.39788700
P	-2.89520700	-0.39345400	0.15338400
O	-2.80456700	-1.44520200	1.20003900
O	-3.42824100	1.04750500	0.59884400

O	-1.53125800	0.12871000	-0.50839100
C	-0.66169400	-0.52157100	-1.41423100
H	0.63594700	-0.21427200	-1.05427000
C	-4.71300000	1.22041300	1.24952200
H	-5.43660400	0.52551600	0.81173500
C	-0.77915000	0.07427800	-2.79959900
H	-0.01996700	-0.36462900	-3.44778900
H	-1.77584800	-0.11618900	-3.21164400
C	-0.67273400	-2.03600400	-1.39699700
H	-1.54882400	-2.44541200	-1.91997500
H	0.22214800	-2.40666200	-1.90311300
C	-4.57515800	0.98364000	2.75274800
H	-3.88503800	1.70869900	3.19611900
H	-5.54745000	1.07982600	3.24594500
C	-5.15269700	2.64746500	0.95740900
H	-6.04525900	2.88712700	1.53749200
Cl	-0.55295600	1.86697500	-2.85621300
H	-4.35321800	3.34692700	1.20766000
O	-3.86339300	-0.81719000	-1.07652400
C	-4.36825100	-2.16919700	-1.23010800
C	-4.77904900	-2.31311600	-2.68737400
C	-5.46912100	-2.49184200	-0.22391700
H	-3.56149500	-2.87389900	-0.99171400
H	-5.16575400	-3.31984200	-2.87367600
H	-5.77083000	-3.53456800	-0.34098500
H	-5.10770800	-2.33161700	0.79078700
H	-3.91946900	-2.14509700	-3.34115700
H	-0.67071800	-2.41251900	-0.37218500
H	-5.55490900	-1.58712500	-2.93925200
H	-4.18745000	-0.02099900	2.93495000
Cl	-5.56882900	2.91949700	-0.77981000
Cl	-6.97320500	-1.49516800	-0.42459200

^{2}IM$_{1\text{-}H}$

S	5.77475700	0.46968800	1.14445200
C	4.48480300	2.52132400	-1.98521600
C	4.97078300	-2.28764000	-1.83224100
C	2.64394200	-2.33195500	2.40806700
C	2.37535900	2.49417200	2.35938800
C	4.82936500	1.21532100	-2.31142900
C	5.51753400	0.83016900	-3.52093100
C	5.67153900	-0.52092800	-3.47491700
C	5.06938200	-0.96467200	-2.24113300
C	4.35704400	-2.72902500	-0.66108500

C	4.25421900	-4.11083800	-0.26009200
C	3.59315200	-4.12125800	0.93020600
C	3.29775500	-2.74580300	1.25120800
C	2.36575200	-1.01738400	2.76789700
C	1.70261900	-0.63092700	3.99162400
C	1.63504800	0.72820800	3.98363500
C	2.25300900	1.16298600	2.75305800
C	2.93203100	2.93427400	1.16575100
C	3.00752600	4.31568600	0.74860700
C	3.58419600	4.31783400	-0.48276300
C	3.86966600	2.93862100	-0.80999700
N	4.57297100	0.10937700	-1.53809000
N	3.76811200	-1.91310600	0.26970500
N	2.69011400	0.08898300	2.03035300
N	3.46636600	2.11435200	0.20622900
Fe	3.57143500	0.09416200	0.22478700
H	4.74601200	3.29164300	-2.70436100
H	5.39701400	-3.04352900	-2.48452700
H	2.32706100	-3.10832000	3.09768100
H	1.98591200	3.24898500	3.03557800
H	6.13879600	-1.17122400	-4.20239900
H	5.83577100	1.51767000	-4.29333500
H	4.63915300	-4.94900000	-0.82545300
H	3.32404500	-4.97031300	1.54417000
H	1.34408600	-1.32004800	4.74456600
H	1.20911400	1.38714100	4.72839800
H	2.65765100	5.15732300	1.33141500
H	3.80884100	5.16239900	-1.12055100
O	1.96731900	0.01027700	-0.62636400
H	6.38834800	-0.61946400	0.63628700
P	-4.23697100	0.22860600	0.11110400
O	-3.82414800	-0.37008000	1.40407200
O	-5.08988000	1.57890800	0.13764600
O	-3.03923300	0.76751700	-0.84564500
C	-1.67913300	0.54408000	-0.76913100
H	1.97799400	-0.75550700	-1.22040800
C	-6.44530200	1.59642700	0.66670000
H	-6.95109600	0.67382600	0.36531800
C	-1.11431100	-0.52814800	-0.01701400
H	-0.03513800	-0.43809800	0.09089900
H	-1.65815700	-0.83839200	0.86769100
C	-0.92863200	1.32740500	-1.78708900
H	-1.20607900	2.38642100	-1.74000100
H	0.14550500	1.21316700	-1.62206500

C	-6.40829400	1.72399300	2.18866200
H	-5.92359700	2.65891100	2.48686500
H	-7.42361600	1.70681500	2.59669000
C	-7.13877000	2.79223100	0.03355500
H	-8.12236100	2.93106300	0.48487800
Cl	-1.21365900	-2.26387000	-1.10332300
H	-6.54163700	3.69493500	0.17362700
Cl	-7.40152900	2.60246700	-1.74532400
O	-5.13401500	-0.70999100	-0.82796400
C	-4.98778900	-2.16218300	-0.85138800
C	-5.54244900	-2.62331700	-2.18840200
C	-5.64078800	-2.79082700	0.37420200
H	-3.92069100	-2.41030500	-0.78880500
H	-5.45378800	-3.71085000	-2.27328100
H	-5.51279400	-3.87424800	0.34193700
H	-5.19951500	-2.39121100	1.28603100
H	-4.97701500	-2.16408000	-3.00219100
Cl	-7.43097500	-2.48309900	0.48527800
H	-5.84494800	0.89283700	2.61789800
H	-6.59452600	-2.34674800	-2.28749400
H	-1.17325700	0.97516900	-2.79976800

^{4}IM$_{1\text{-}H}$

S	5.87045200	-0.24210900	0.39369300
C	4.30918800	2.72772700	-1.91151600
C	3.94795900	-2.05320000	-2.61729600
C	2.92135200	-2.66714000	2.07183900
C	3.33918700	2.10852300	2.78879000
C	4.34298600	1.47023100	-2.49640400
C	4.61284300	1.23051100	-3.89147000
C	4.51334600	-0.11301500	-4.08883300
C	4.18109000	-0.70012500	-2.81551700
C	3.58484200	-2.62784100	-1.40899000
C	3.38485100	-4.04119500	-1.20559200
C	3.09163200	-4.21318200	0.11202200
C	3.11324900	-2.90591400	0.71973400
C	2.99829600	-1.42146000	2.67756700
C	2.76015400	-1.18597500	4.07892300
C	2.88140300	0.15526600	4.28156800
C	3.19221900	0.74576400	3.00451700
C	3.58863000	2.69286400	1.55681400
C	3.76649000	4.10965400	1.34950600
C	4.03610700	4.28348600	0.02721600
C	4.02558300	2.97364300	-0.57738600

N	4.09852900	0.28072300	-1.85756500
N	3.39224100	-1.95373000	-0.22860400
N	3.28170500	-0.23370700	2.04599000
N	3.73339200	2.02432400	0.36836900
Fe	3.59754300	0.04080500	0.07514600
H	4.52668400	3.58102600	-2.54492200
H	4.05835400	-2.71095100	-3.47266800
H	2.69499300	-3.51840200	2.70467700
H	3.24748400	2.76489400	3.64757100
H	4.64001400	-0.67171600	-5.00662200
H	4.83871600	2.00369300	-4.61376800
H	3.46856300	-4.79002300	-1.98189900
H	2.88390000	-5.13247800	0.64310700
H	2.52039400	-1.95814200	4.79782800
H	2.76174200	0.71210600	5.20150300
H	3.69819400	4.85699500	2.12892300
H	4.23602100	5.20345800	-0.50601300
O	1.84504500	0.25526200	-0.25635200
H	5.84103600	-0.47716100	1.72136200
P	-4.37491000	0.43731200	0.37163500
O	-4.06145400	-0.08562900	1.72262100
O	-5.38232400	1.66592500	0.24131900
O	-3.12813000	1.13404300	-0.42753700
C	-1.78042000	1.04208400	-0.20302800
H	1.43169800	-0.59599800	-0.48930900
C	-6.78691100	1.52002500	0.60379400
H	-7.12306500	0.52145100	0.30745600
C	-1.20680300	0.16060200	0.72933300
H	-0.15606100	0.33381600	0.93233100
H	-1.82123600	-0.21376700	1.53761900
C	-0.99860900	1.84655400	-1.18169600
H	-1.25092800	2.91018900	-1.09072700
H	0.07144200	1.70041900	-1.02160500
C	-6.95538900	1.72826800	2.10717800
H	-6.63734400	2.73460600	2.39667300
H	-8.00255700	1.59272000	2.39459200
C	-7.54570600	2.57263200	-0.18832600
H	-8.58814800	2.59614500	0.13271400
Cl	-0.91254300	-1.75311500	-0.28696900
H	-7.09575900	3.55594400	-0.04109300
Cl	-7.55202600	2.25390300	-1.96763300
O	-4.98862000	-0.61599200	-0.66199000
C	-4.64171900	-2.03694600	-0.64460500
C	-4.95704000	-2.56516900	-2.03339500

C	-5.35043700	-2.75102200	0.49989300
H	-3.56675500	-2.13210100	-0.44672400
H	-4.70733400	-3.62909200	-2.09102200
H	-5.07573300	-3.80725100	0.49595300
H	-5.08073900	-2.30187400	1.45450100
H	-4.36401400	-2.02925100	-2.77774100
Cl	-7.16470300	-2.68438300	0.38797600
H	-6.34746500	1.00418000	2.65374400
H	-6.01674800	-2.43866800	-2.26633600
H	-1.25487200	1.54342600	-2.20455300

^{4}TS$_{1\text{-}OH}$

S	3.78545100	1.00764100	1.96858400
C	1.26560700	3.58809900	0.09454200
C	4.12635600	0.29781600	-2.02356200
C	2.73570500	-3.01082800	1.22217000
C	-0.12151900	0.27925100	3.35153400
C	2.19376900	2.98227100	-0.74153300
C	2.84408300	3.64178000	-1.84671500
C	3.64986100	2.71763500	-2.43860900
C	3.49401300	1.48974000	-1.69873300
C	3.96400200	-0.90080100	-1.34425000
C	4.65045200	-2.12688000	-1.67808600
C	4.26055900	-3.05737600	-0.76476800
C	3.33465200	-2.40186300	0.12899900
C	1.86537600	-2.38454300	2.10468400
C	1.22501800	-3.04296100	3.21556700
C	0.42234400	-2.11926700	3.81487600
C	0.56919800	-0.89278100	3.07180400
C	-0.01869400	1.45490700	2.62233600
C	-0.68892600	2.68506600	2.96329700
C	-0.29775000	3.61616500	2.04849600
C	0.61225600	2.95792400	1.14387800
N	2.61307300	1.67801000	-0.66350500
N	3.15825800	-1.09823000	-0.25390600
N	1.46533600	-1.07152900	2.04619000
N	0.74984300	1.63675600	1.49741100
Fe	1.98375700	0.28284600	0.64228100
H	1.03817500	4.63382900	-0.08075900
H	4.80225700	0.30584400	-2.87205100
H	2.96573000	-4.05544900	1.40317900
H	-0.78861600	0.27690800	4.20671500
H	4.29195300	2.83690200	-3.30119600
H	2.68731800	4.67626100	-2.12190900

H	5.34239800	-2.23900200	-2.50226600
H	4.56476000	-4.09251200	-0.68412400
H	1.37760000	-4.07984400	3.48476400
H	-0.21800900	-2.24056500	4.67865200
H	-1.35884300	2.80526500	3.80428600
H	-0.58260100	4.65744200	1.98051500
O	0.63213700	-0.26410800	-0.48164400
H	3.62645900	0.13138200	2.98146300
P	-3.05928100	-0.45192800	-1.50452400
O	-3.58347700	0.09551300	-2.77042400
O	-4.15556600	-1.20972700	-0.62373400
O	-2.32994100	0.62433900	-0.53706400
O	-1.90692800	-1.59569800	-1.57067600
C	-0.67719600	-1.52928900	-2.19859600
H	-0.18055800	0.21962200	-0.25283300
C	-3.91039800	-2.28456600	0.32473600
H	-3.22669400	-2.99700900	-0.15076800
C	0.16215400	-2.68543500	-1.90502600
H	1.20141300	-2.50451900	-2.16292400
H	0.06854300	-3.01804800	-0.87517700
C	-0.51542900	-0.68905500	-3.41503500
H	-0.84269700	0.34044800	-3.25206000
H	0.53603100	-0.67651300	-3.70772900
C	-3.18729400	-1.79594800	1.57658200
H	-2.94109500	-2.65337400	2.20561300
C	-5.25550400	-2.94609500	0.57624100
H	-5.13267400	-3.79266500	1.25879100
Cl	-0.33637700	-4.19834200	-2.93685500
H	-5.95863600	-2.23797700	1.01949100
C	-2.78988500	2.01873500	-0.41102300
C	-4.29592400	2.12339600	-0.22071500
C	-2.23978700	2.77695600	-1.62211700
H	-2.27524500	2.36125700	0.48847400
H	-4.83356300	1.79469000	-1.11422300
H	-4.55812600	3.16642400	-0.02667100
H	-1.17208500	2.58512100	-1.73215400
H	-2.77083800	2.50442200	-2.53373300
Cl	-2.41815300	4.56904700	-1.39738400
H	-1.10981000	-1.08802900	-4.24962900
H	-5.66939100	-3.31301200	-0.36548900
H	-2.27054400	-1.26344100	1.32631800
Cl	-4.16749800	-0.66780900	2.60865900
H	-4.61178200	1.51967300	0.63266600

^{2}**IM$_{1\text{-OH}}$**

S	-4.21994800	-0.88551200	1.76727700
C	-4.22591800	1.95662600	-1.17900500
C	-1.33951000	2.02807300	2.70144700
C	-0.56810600	-2.71801400	2.15719900
C	-3.12059300	-2.69020100	-1.95009400
C	-3.54306000	2.37322300	-0.04598100
C	-3.57930600	3.71792800	0.47395700
C	-2.75048100	3.74477600	1.55270500
C	-2.21500400	2.41440400	1.69723700
C	-0.85523400	0.73992700	2.87299600
C	-0.01485200	0.32539500	3.96630000
C	0.18166700	-1.01424700	3.82398200
C	-0.52858400	-1.41773000	2.63774700
C	-1.21445500	-3.12189900	0.99833600
C	-1.17050900	-4.46511600	0.47425200
C	-1.86295000	-4.45264800	-0.69688300
C	-2.34154100	-3.10548000	-0.88120000
C	-3.63058800	-1.40999800	-2.10193300
C	-4.48999200	-1.00066000	-3.18349300
C	-4.81942500	0.29918300	-2.95173200
C	-4.15349600	0.68753300	-1.73354000
N	-2.70146800	1.58732300	0.70879000
N	-1.14475900	-0.33163400	2.05171500
N	-1.93576400	-2.30156900	0.16089700
N	-3.42235400	-0.36478400	-1.22864100
Fe	-2.44262100	-0.39552700	0.51325000
H	-4.84736300	2.68810400	-1.68544000
H	-1.03534600	2.78024500	3.42177900
H	-0.03622700	-3.47614600	2.72310700
H	-3.36852700	-3.42490700	-2.70917300
H	-2.51268500	4.57792200	2.20059900
H	-4.16131700	4.52576600	0.05053100
H	0.35996300	0.98422500	4.73815600
H	0.75336000	-1.68172100	4.45517600
H	-0.65994900	-5.29348900	0.94741800
H	-2.04192200	-5.26903800	-1.38392000
H	-4.79392800	-1.63838400	-4.00297300
H	-5.44836200	0.95137900	-3.54320400
O	0.19902900	0.26457500	-0.80070700
H	-4.67315600	-1.92749900	1.03783100
P	3.30062200	0.27457800	-1.34008500
O	3.48158400	-0.41159300	-2.63646500
O	4.59300800	1.11870800	-0.89882700

O	2.98122100	-0.67203400	-0.07080400
O	2.17958600	1.42972300	-1.24471100
C	0.77260500	1.29611200	-1.56453800
H	0.39832300	0.40470800	0.13957200
C	4.92070100	1.58236800	0.43315900
H	4.74714900	0.76473800	1.13909400
C	0.14449800	2.62635400	-1.12275300
H	-0.92682300	2.59475800	-1.31321200
H	0.32998400	2.78549700	-0.06019500
C	0.55303000	0.96240400	-3.03099400
H	1.04178800	0.01897700	-3.27527300
H	-0.51988700	0.87613900	-3.22059000
C	6.40449000	1.93565000	0.38326200
H	6.58050300	2.76956000	-0.30308900
H	6.76070400	2.21695800	1.37869700
C	4.07856800	2.80365500	0.83670700
H	4.67612300	3.50843400	1.41509300
Cl	0.80484600	4.07352000	-1.98658900
H	3.67323200	3.29817600	-0.04578200
Cl	2.66745800	2.38798500	1.90865400
C	3.00254300	-2.13427200	-0.09732500
C	4.44494700	-2.58384000	-0.34816700
C	1.98231600	-2.71199900	-1.06819100
H	2.72697000	-2.39293800	0.92870700
H	5.13159900	-2.04567300	0.30635700
H	4.72847100	-2.43523100	-1.39048100
H	1.00381200	-2.26479000	-0.88486200
H	2.27460800	-2.51267600	-2.10099700
Cl	4.65006600	-4.34675800	0.02543100
H	0.96975600	1.74898700	-3.66119100
H	1.90972100	-3.79212500	-0.91897600
H	6.98151800	1.07715400	0.03219800

^{4}IM$_{1\text{-OH}}$

S	5.07954600	1.49885700	-0.38623700
C	3.01547000	-1.15959200	-3.04475100
C	4.42778700	-2.31047200	1.44278000
C	2.40490200	1.74748800	3.12099300
C	1.08989900	2.95619000	-1.38388600
C	3.60034500	-1.80579700	-1.96545900
C	4.29299600	-3.06371600	-2.05618900
C	4.69253100	-3.38608100	-0.79408800
C	4.24375000	-2.32627500	0.06865700
C	3.96241900	-1.31763900	2.28930500

C	4.13869300	-1.32643100	3.71978500
C	3.56210800	-0.18896900	4.19321200
C	3.03702400	0.51690500	3.05150000
C	1.90826200	2.44157300	2.02800900
C	1.24868600	3.71625200	2.11412300
C	0.89158700	4.06304800	0.84438000
C	1.32478400	2.99547000	-0.01687200
C	1.44821100	1.90733000	-2.21508200
C	1.20927700	1.87694900	-3.63323900
C	1.74851800	0.71612200	-4.09921300
C	2.31970200	0.03604300	-2.96711600
N	3.58226700	-1.36270700	-0.66081700
N	3.27808200	-0.19065100	1.89695800
N	1.95851200	2.01583800	0.71764400
N	2.10304800	0.75991800	-1.81172700
Fe	2.95195900	0.42339400	0.00402000
H	3.11364200	-1.62224800	-4.02104900
H	4.96401100	-3.14187200	1.88776400
H	2.28829200	2.20039000	4.09973600
H	0.58473400	3.80430600	-1.83382300
H	5.23593500	-4.26060500	-0.46187200
H	4.44176100	-3.61901800	-2.97274600
H	4.64054700	-2.10950200	4.27237900
H	3.49185600	0.15750100	5.21568800
H	1.08575200	4.26320400	3.03329600
H	0.37848400	4.95447800	0.50830200
H	0.69627600	2.65212500	-4.18653500
H	1.77400500	0.34608300	-5.11557200
O	-0.07602600	-0.90988700	-0.04139900
H	4.76126200	2.64736200	0.24633800
P	-3.07834100	-0.52043300	0.26284800
O	-2.97821300	-0.92463300	1.68891800
O	-2.64001400	0.97554300	-0.11536700
O	-2.24135100	-1.39963800	-0.79366200
C	-0.90506400	-1.91884100	-0.53807500
H	0.20501100	-0.32731800	-0.76541800
C	-3.34413200	2.11495100	0.44810400
H	-4.41867600	1.90551600	0.42611900
C	-0.92512700	-2.96290300	0.58974200
H	0.07834300	-3.36747100	0.71730800
H	-1.27498300	-2.49649900	1.50878800
C	-0.43975200	-2.45805500	-1.88365700
H	-0.43933500	-1.64927300	-2.61996600
H	0.57291700	-2.85727300	-1.78982300

C	-2.87284200	2.38679300	1.87668100
H	-1.80792400	2.63826600	1.89135800
H	-3.43946600	3.21800600	2.30876100
C	-3.03204600	3.29883200	-0.45311100
H	-3.44973800	4.21145100	-0.02541200
Cl	-2.02581300	-4.35660300	0.24532200
H	-1.95221100	3.40548200	-0.56708100
Cl	-3.72450500	3.14057000	-2.11822400
O	-4.56090300	-0.58184800	-0.35830100
C	-5.45091300	-1.67992400	-0.02664200
C	-6.41336700	-1.82947700	-1.19381200
C	-6.10885000	-1.44850700	1.33089200
H	-4.85706600	-2.59769200	0.08287700
H	-7.10677500	-2.65562500	-1.00730400
H	-6.75895500	-2.29035000	1.57656700
H	-5.34705000	-1.32678500	2.09966400
H	-5.85618500	-2.03919100	-2.10969200
Cl	-7.14726000	0.04261600	1.38425600
H	-3.02629500	1.49882900	2.49280200
H	-6.99021600	-0.91275000	-1.33509000
H	-1.10810500	-3.24557400	-2.23457600

$IM_{1\text{-}OH}$

O	0.16657600	2.91821800	-0.23727800
P	-0.13680800	0.09230500	-0.92631200
O	-0.48518800	0.54557400	-2.30068500
O	0.24540300	-1.45569500	-0.91160500
O	-1.32308500	0.26873100	0.15208100
O	1.13967400	0.75529100	-0.22039200
C	1.18885800	2.15070000	0.28149900
H	0.34399000	3.12592400	-1.19439800
C	0.59822200	-2.17568300	0.30666200
H	0.29940500	-1.57478300	1.17432200
C	0.91244600	2.10877800	1.78999500
H	0.97896500	3.11838600	2.19445300
H	-0.08374100	1.70356000	1.95748300
C	2.57472200	2.65454500	-0.09937400
H	2.66174100	2.66927100	-1.18829700
H	2.70933800	3.67063700	0.28079400
C	-0.17354000	-3.48446200	0.30166500
H	0.06352700	-4.06571900	-0.59183300
H	0.08876400	-4.07672000	1.18398800
C	2.11347200	-2.31019900	0.39311300
H	2.38493300	-2.78488400	1.33788700

Cl	2.07528100	1.08121300	2.71819300
O	0.35736600	3.05130600	-2.89438600
H	0.01159700	2.12916000	-2.90870400
H	1.18643400	3.02365300	-3.38720800
C	-2.65630300	0.68457400	-0.25507500
C	-3.16938900	1.66365700	0.78726500
C	-3.53101800	-0.54086600	-0.48983300
H	-2.57233000	1.18131000	-1.22828600
H	-4.17141700	2.01007900	0.51543100
H	-3.22090400	1.19400000	1.77237100
H	-3.07780100	-1.20090900	-1.23002000
H	3.35595500	2.01202500	0.30830700
H	-1.24695300	-3.28380600	0.32706400
H	-2.50073900	2.52592700	0.83110400
H	2.58318300	-1.33017300	0.32986600
Cl	2.83346400	-3.32492300	-0.92671900
H	-4.51701400	-0.23074000	-0.84075300
Cl	-3.80142800	-1.54302200	0.99992100

TS_{BCIPP}

O	-2.87388800	-0.49337200	-1.59041700
P	0.02194300	0.05517400	-0.17179800
O	0.42832600	0.26265200	-1.63140100
O	1.22217400	0.58096500	0.78896200
O	-0.00614900	-1.51587500	0.20566200
O	-1.29856200	0.65707400	0.28708400
C	-3.11156000	0.15937000	-0.52707700
H	-2.27413400	0.07579400	-2.46515200
C	1.53010600	2.00135800	0.82725500
H	0.63579500	2.56942700	0.54904100
C	-3.54309200	-0.75097800	0.61054900
H	-4.43319600	-1.28896500	0.27409900
H	-2.74172700	-1.46047600	0.80611600
C	-3.54620600	1.59596100	-0.58731900
H	-3.00868900	2.11540500	-1.38157400
H	-4.61832200	1.60707100	-0.82308000
C	1.93422000	2.33296600	2.25365500
H	2.79282700	1.72471300	2.55567600
H	2.20598500	3.38790000	2.33774900
C	2.62612800	2.23781700	-0.21431600
H	3.55988200	1.75824600	0.08389800
Cl	-3.97043600	0.06391800	2.15494000
H	2.30884700	1.86220700	-1.18589100
Cl	2.97518400	4.00965100	-0.41459400

O	-1.42893400	0.49566100	-3.21494700
H	-0.55485400	0.42111300	-2.58773900
H	-1.55527700	1.42068400	-3.46269100
C	0.98531700	-2.44224500	-0.31806700
C	0.24848700	-3.70257600	-0.74006400
C	1.99865900	-2.64837000	0.80950900
H	1.48071300	-1.98332000	-1.17914200
H	0.95211400	-4.44771600	-1.11864900
H	1.54189500	-3.16189300	1.65702900
Cl	3.40396500	-3.65983800	0.26318400
H	-0.29154800	-4.13090700	0.11051700
H	2.40168900	-1.69061000	1.13516900
H	-3.38784800	2.10524500	0.35982700
H	1.10512000	2.12536600	2.93404300
H	-0.47262200	-3.46696800	-1.52657100

P_{BCIPP}

O	-2.89840900	-0.41790200	0.46436200
P	1.40891300	-0.85488000	0.10090100
O	1.03829600	-1.47276800	1.41087000
O	2.87111000	-1.19067200	-0.46369000
O	1.46061400	0.75809800	0.12739000
O	0.46650200	-1.25946600	-1.10601300
C	-4.00945600	-0.20865800	-0.00015000
H	-1.90006500	-2.00348400	0.45117800
C	4.04399400	-0.98648700	0.36429300
H	3.88807000	-0.10714600	0.99837200
C	-4.58552700	1.17349800	-0.10931100
H	-5.41556300	1.28081200	0.59800800
H	-3.81045700	1.90762200	0.11191500
C	-4.78552300	-1.41427400	-0.52855000
H	-4.46560300	-1.59962500	-1.55867700
H	-4.54392300	-2.29017900	0.07368200
C	4.29300600	-2.22750000	1.21916800
H	4.46817100	-3.10220900	0.58529000
H	5.16392000	-2.08077900	1.86567600
C	5.20637800	-0.72488300	-0.58025900
H	6.14501800	-0.70892000	-0.02458700
Cl	-6.57892400	-1.22193900	-0.55483500
H	5.24895100	-1.49709200	-1.34990700
Cl	5.08125500	0.86616500	-1.43531400
O	-1.23598500	-2.69538100	0.26742100
H	-0.58368700	-2.59133300	0.98824800
H	-0.23999300	-1.89650000	-0.76401100

C	0.32782300	1.49873400	0.66469100
C	0.16228700	2.64861200	-0.32830300
C	0.63765400	1.91941100	2.09194200
H	-0.57320300	0.87640000	0.63694300
H	1.02715600	3.31314400	-0.30830400
H	1.55367700	2.51812000	2.12110900
Cl	-1.29561300	3.66381200	0.05378300
H	0.02320800	2.25857000	-1.33595300
H	0.77666500	1.02885500	2.70886500
H	-5.00648000	1.34124300	-1.10573100
H	-0.18244600	2.51176300	2.50545700
H	3.41784000	-2.41870700	1.84326700

Cartesian coordinates of reaction species for TCEP C_1-hydroxylation and O-dealkylation

^{2}RC

S	-4.53457500	-0.41232400	1.55714500
C	-2.94021800	3.20573600	0.50217300
C	-0.29961000	0.01782500	3.00819000
C	-2.07797100	-3.57051000	0.29977000
C	-4.45862400	-0.34652000	-2.40352000
C	-2.07998300	2.64216300	1.44210000
C	-1.37700400	3.39142100	2.45452500
C	-0.62459500	2.49465100	3.15206000
C	-0.87796600	1.20084900	2.56895400
C	-0.55744100	-1.24295500	2.48559900
C	0.01387400	-2.46422700	2.99621600
C	-0.50316000	-3.47763100	2.24831700
C	-1.37627200	-2.86773400	1.27455800
C	-2.88299900	-3.00398300	-0.67727300
C	-3.56163800	-3.74798700	-1.71069300
C	-4.21089300	-2.83756000	-2.48500900
C	-3.94021000	-1.53943800	-1.91562300
C	-4.27987800	0.89977000	-1.82533800
C	-4.87491500	2.12053300	-2.31166800
C	-4.46249100	3.11817800	-1.48491400
C	-3.60453600	2.51310400	-0.49575000
N	-1.77053700	1.31108800	1.53218100
N	-1.39739500	-1.50865700	1.43661500
N	-3.13070800	-1.66016100	-0.81589600
N	-3.51876300	1.15347200	-0.70718200
Fe	-2.32890200	-0.15938500	0.27262600
H	-3.09540400	4.27896900	0.55137700

H	0.42041200	0.08316400	3.81528400
H	-1.97198200	-4.65077200	0.29311300
H	-5.08160700	-0.39997700	-3.29045500
H	0.04060800	2.67299800	3.98609600
H	-1.46041100	4.46035100	2.60023700
H	0.71568100	-2.51727400	3.81713100
H	-0.31880900	-4.54043400	2.33167900
H	-3.53217900	-4.82440700	-1.81504600
H	-4.82832900	-3.01054200	-3.35629200
H	-5.52426200	2.18551500	-3.17448300
H	-4.70106700	4.17249100	-1.52976500
O	-1.05928100	0.06228300	-0.72637700
H	-4.57462100	0.83103200	2.08033500
H	0.80261700	-1.24687500	-0.45733900
P	3.18583900	0.45692600	0.58250200
O	2.69990300	-0.44694800	-0.66117000
O	2.91217400	1.95119500	0.03777700
O	2.62153300	0.09491300	1.89943600
O	4.78506100	0.42098700	0.53412700
C	5.49413300	0.53455500	-0.71474400
C	6.96346000	0.74169000	-0.42173700
H	5.12590700	1.40704300	-1.26828100
H	5.32580300	-0.36700700	-1.31004200
H	7.49532000	0.95668000	-1.35042700
H	7.10636500	1.56334000	0.28114800
C	1.84242000	-1.59329100	-0.47549000
C	2.01274800	-2.51578000	-1.65952000
H	2.07549100	-2.09564600	0.46602900
H	1.28028300	-3.32293400	-1.60396000
H	1.88891600	-1.97413100	-2.59786700
C	1.56544000	2.36590800	-0.25818400
C	1.62046800	3.61301800	-1.11147400
H	1.05364800	2.61389100	0.67958400
H	1.01088300	1.56691500	-0.75895800
H	0.61150000	4.00225100	-1.25750100
H	2.24787700	4.37696300	-0.65007400
Cl	2.30940600	3.30611100	-2.76136400
Cl	7.75047000	-0.71720300	0.30731200
Cl	3.65296500	-3.29329800	-1.71660600

^{4}RC

S	-4.51814100	-0.41827700	1.57662100
C	-2.95148000	3.20843300	0.49721800
C	-0.29607500	0.02623000	2.99401100

C	-2.08253400	-3.56762000	0.29965600
C	-4.49052400	-0.35194400	-2.38933500
C	-2.08209900	2.64855100	1.43101100
C	-1.37205300	3.39974500	2.43710200
C	-0.61717000	2.50354500	3.13266200
C	-0.87560600	1.20845200	2.55425000
C	-0.55388700	-1.23600300	2.47440000
C	0.02595000	-2.45525900	2.98117600
C	-0.49207700	-3.47048200	2.23649600
C	-1.37469300	-2.86323400	1.26919200
C	-2.89530600	-3.00329100	-0.67202300
C	-3.58213300	-3.75061600	-1.69760100
C	-4.23928000	-2.84316900	-2.46874200
C	-3.96507000	-1.54339800	-1.90514700
C	-4.31002800	0.89454000	-1.81248700
C	-4.91708100	2.11241600	-2.29043800
C	-4.49844600	3.11192900	-1.46913400
C	-3.62475600	2.51141300	-0.49135200
N	-1.77347900	1.31782000	1.52164400
N	-1.39979000	-1.50505400	1.43175100
N	-3.14509000	-1.65956400	-0.81306000
N	-3.53368000	1.15182600	-0.70509200
Fe	-2.34317600	-0.15546100	0.26804800
H	-3.10933300	4.28131000	0.54545600
H	0.42784000	0.09380700	3.79742600
H	-1.97537900	-4.64778200	0.29298000
H	-5.12297500	-0.40725300	-3.26941900
H	0.05290500	2.68291000	3.96257400
H	-1.45329800	4.46912100	2.58073100
H	0.73401000	-2.50549700	3.79687800
H	-0.30272400	-4.53256000	2.31792700
H	-3.55259300	-4.82731200	-1.79906100
H	-4.86440000	-3.01926300	-3.33389000
H	-5.57815900	2.17427100	-3.14451800
H	-4.74270700	4.16507300	-1.51058500
O	-1.05676800	0.04909700	-0.71553000
H	-4.56226500	0.82648900	2.09597400
H	0.81701000	-1.25565100	-0.44631100
P	3.19522100	0.45953200	0.58182400
O	2.71039400	-0.44862300	-0.65910700
O	2.92278100	1.95157700	0.03045500
O	2.62891600	0.10287000	1.89937400
O	4.79449000	0.42288800	0.53632600
C	5.50582000	0.53206300	-0.71170500

C	6.97489000	0.73818200	-0.41672800
H	5.13958000	1.40344200	-1.26829700
H	5.33737400	-0.37095600	-1.30476000
H	7.50865300	0.94974800	-1.34511300
H	7.11764800	1.56170900	0.28398500
C	1.85795200	-1.59809800	-0.46934900
C	2.02651900	-2.52006100	-1.65406500
H	2.09702000	-2.09962500	0.47107500
H	1.29754900	-3.33012200	-1.59500400
H	1.89620600	-1.97907900	-2.59192600
C	1.57677500	2.36532700	-0.26995300
C	1.63396800	3.61010500	-1.12654100
H	1.06234300	2.61554700	0.66577800
H	1.02406100	1.56474100	-0.77018300
H	0.62550500	3.99941200	-1.27582300
H	2.26065600	4.37504700	-0.66582300
Cl	2.32632600	3.29829500	-2.77404200
Cl	7.75868300	-0.71959700	0.31798000
Cl	3.66947900	-3.29106800	-1.71846200

^{2}TS$_{1\text{-}H}$

S	-4.41033600	-0.46312300	1.20783500
C	-2.84137500	3.19621000	0.17243900
C	-0.58037400	0.27711900	3.30091900
C	-1.76113100	-3.56037200	0.61109400
C	-3.70686800	-0.59978700	-2.68492200
C	-2.15296300	2.72690000	1.29026600
C	-1.65705600	3.56653700	2.35396500
C	-1.01046300	2.74574000	3.22937700
C	-1.12066600	1.40859900	2.69918200
C	-0.68144700	-1.02341100	2.82392400
C	-0.17138000	-2.18227300	3.51663200
C	-0.52103300	-3.26632900	2.77306500
C	-1.23275300	-2.76354200	1.62187000
C	-2.41621800	-3.09168300	-0.52110400
C	-2.89746700	-3.92191400	-1.59818400
C	-3.41832700	-3.08244400	-2.53458100
C	-3.26919200	-1.74111500	-2.02377100
C	-3.65448000	0.69708100	-2.19137200
C	-4.18026700	1.85785000	-2.87402100
C	-3.95475900	2.92542900	-2.06308600
C	-3.28096800	2.41937400	-0.88913000
N	-1.82540400	1.41838000	1.52497800
N	-1.30862600	-1.39511800	1.66296100

N	-2.65711100	-1.76714500	-0.79436700
N	-3.11744100	1.05841600	-0.98287900
Fe	-2.14880400	-0.16719500	0.32103100
H	-3.03460100	4.26346500	0.12180000
H	-0.03259100	0.42141400	4.22589800
H	-1.63313100	-4.63419500	0.70481500
H	-4.16266100	-0.74012600	-3.66006200
H	-0.50491700	3.00460800	4.15011000
H	-1.79362400	4.63854500	2.40988600
H	0.37767600	-2.15000900	4.44772400
H	-0.32277700	-4.31173700	2.96813300
H	-2.83194200	-5.00152800	-1.62183000
H	-3.87189200	-3.33231500	-3.48447300
H	-4.66014000	1.83739000	-3.84346700
H	-4.20862000	3.96388900	-2.22967500
O	-0.68702900	0.16346900	-0.57306800
H	-4.70477900	0.83647700	1.41861300
H	0.20678700	-0.61586300	-0.47185200
P	2.99154300	0.44235500	0.63321700
O	2.37587800	-0.37469400	-0.63652300
O	2.87889200	1.97012900	0.14402600
O	2.41461900	0.08793100	1.94349800
O	4.56634100	0.20820400	0.51404400
C	5.25901400	0.34897500	-0.74320000
C	6.74818500	0.34697800	-0.47892800
H	4.98810400	1.30596600	-1.20479200
H	4.96843600	-0.46861300	-1.40835600
H	7.28267100	0.58240700	-1.40117200
H	7.00582000	1.07630400	0.29005000
C	1.34152400	-1.31410000	-0.48068300
C	1.21914600	-2.18428800	-1.68541300
H	1.37061200	-1.81969800	0.48533400
H	0.32027100	-2.79619900	-1.62134000
H	1.20905000	-1.59370400	-2.60119000
C	1.58511500	2.58611100	-0.03319000
C	1.77663300	3.89189300	-0.76976000
H	1.15774600	2.80550500	0.95275400
H	0.90683000	1.91374300	-0.56689200
H	0.82582700	4.42495200	-0.82120800
H	2.52290300	4.51678400	-0.27711600
Cl	2.34241600	3.65939100	-2.47754200
Cl	7.36081200	-1.25849600	0.08897200
Cl	2.62782800	-3.35554300	-1.85090600

$^4TS_{1\text{-}H}$

S	-4.36059600	-0.64632900	1.28355000
C	-3.23620200	3.03636400	-0.27019100
C	-0.71754800	0.82798900	3.21634700
C	-1.44056100	-3.44003800	1.06971400
C	-3.55018600	-1.16582500	-2.63929900
C	-2.49764300	2.80881600	0.89134000
C	-2.11498800	3.83148200	1.83584800
C	-1.39159600	3.20893200	2.80853300
C	-1.34434200	1.80923100	2.45587000
C	-0.69096200	-0.53356600	2.93287500
C	-0.09173700	-1.52777100	3.79023000
C	-0.31783400	-2.73411600	3.20304400
C	-1.04048800	-2.46575500	1.98227300
C	-2.08003000	-3.19617500	-0.13800200
C	-2.42640400	-4.20904300	-1.10615500
C	-2.98846900	-3.56244500	-2.16359200
C	-3.00164400	-2.15701900	-1.83373900
C	-3.68204100	0.17458100	-2.30091100
C	-4.34184700	1.16424300	-3.12019600
C	-4.27853200	2.34159700	-2.44287100
C	-3.56668000	2.07718200	-1.21506400
N	-2.02280800	1.59116900	1.28637500
N	-1.25424800	-1.12294000	1.83463600
N	-2.43925000	-1.95085700	-0.59971300
N	-3.22306100	0.74559000	-1.13976900
Fe	-2.15698900	-0.16534400	0.30118100
H	-3.56030900	4.05474700	-0.46168200
H	-0.21241100	1.15085000	4.12069900
H	-1.21012800	-4.47291100	1.31156300
H	-3.95582700	-1.47582000	-3.59727200
H	-0.93738600	3.63889300	3.69141200
H	-2.37601900	4.87841400	1.75462700
H	0.43277600	-1.31219100	4.71081600
H	-0.02186700	-3.71733400	3.54332200
H	-2.25242000	-5.26919000	-0.97884900
H	-3.37551400	-3.98357100	-3.08190700
H	-4.79283400	0.96445700	-4.08307800
H	-4.66304200	3.30940700	-2.73610400
O	-0.65189000	0.18341300	-0.51965900
H	-4.95746200	0.53547600	1.02436300
H	0.22584500	-0.61501500	-0.46106800
P	2.98522200	0.50480700	0.62586200
O	2.38942000	-0.34007900	-0.63257000

O	2.87063600	2.02196600	0.10296500
O	2.39812400	0.17808000	1.93888500
O	4.56365500	0.27854000	0.53112300
C	5.26926900	0.38451300	-0.72168100
C	6.75579000	0.37484000	-0.44262600
H	5.01237800	1.33369200	-1.20713100
H	4.97792100	-0.44455600	-1.37215600
H	7.30169800	0.58441500	-1.36441300
H	7.01282700	1.11865000	0.31255700
C	1.37639600	-1.30469600	-0.47179300
C	1.28510900	-2.18442200	-1.67137300
H	1.41668000	-1.80282400	0.49788200
H	0.40602700	-2.82447400	-1.61080100
H	1.26332200	-1.59944600	-2.59065000
C	1.57514300	2.63090000	-0.08257400
C	1.76073400	3.92370600	-0.84300800
H	1.14916000	2.86717200	0.90013200
H	0.89677700	1.94559500	-0.59949800
H	0.80998300	4.45671300	-0.89608700
H	2.51096800	4.55663400	-0.36690000
Cl	2.31340500	3.66201200	-2.55104700
Cl	7.34786800	-1.22310000	0.16731000
Cl	2.72999700	-3.31578300	-1.82743100

^{2}IM$_{1\text{-}H}$

S	-4.01070500	-0.25482500	2.14687000
C	-2.63517100	3.38677300	0.70833800
C	-0.07369500	-0.10799300	2.84816500
C	-2.35325700	-3.45393600	0.20408200
C	-4.63057600	0.07441200	-2.19198000
C	-1.77268700	2.72404800	1.57195000
C	-0.92476900	3.36495000	2.55042100
C	-0.18910500	2.38010300	3.13359900
C	-0.59910200	1.13622200	2.52324400
C	-0.47938100	-1.32993700	2.32037600
C	0.02801000	-2.61097800	2.75363300
C	-0.63427500	-3.55890800	2.03366100
C	-1.53045600	-2.85420300	1.14936800
C	-3.16359800	-2.77877700	-0.70578400
C	-3.97448900	-3.42018200	-1.71233400
C	-4.59953200	-2.42437600	-2.39855500
C	-4.17346200	-1.18104800	-1.80094500
C	-4.29406700	1.28951400	-1.60455800
C	-4.81293900	2.57033500	-2.02576200

C	-4.26090600	3.50265400	-1.20232700
C	-3.40407000	2.78602100	-0.28662800
N	-1.57125700	1.36551200	1.58141000
N	-1.42546700	-1.49898400	1.34267900
N	-3.29349600	-1.41803400	-0.77950000
N	-3.44212500	1.44380200	-0.54443000
Fe	-2.39666000	-0.02103300	0.35511700
H	-2.69877400	4.46680900	0.80009500
H	0.72380700	-0.12971900	3.58188700
H	-2.34407800	-4.53809600	0.15112900
H	-5.32788800	0.10627100	-3.02366300
H	0.56474600	2.46629200	3.90449000
H	-0.90108400	4.42912200	2.74457700
H	0.77998900	-2.74631200	3.51955200
H	-0.53565200	-4.63527000	2.08330600
H	-4.04216000	-4.48932800	-1.86269600
H	-5.29022400	-2.50691300	-3.22718900
H	-5.50653400	2.71793600	-2.84289600
H	-4.40539600	4.57487600	-1.20369600
O	-1.01261000	0.19972800	-0.80168200
H	-4.33462700	1.04682100	2.29236000
H	-0.73625000	-0.67656700	-1.11444200
P	3.54198600	0.29292900	0.64975500
O	3.26475600	-0.86517100	-0.50034100
O	3.05994100	1.60636500	-0.10938800
O	2.98176200	-0.05122400	1.96909500
O	5.12687300	0.47381100	0.63647400
C	5.84107100	0.86558200	-0.55489700
C	7.32401900	0.75676400	-0.27987500
H	5.59216600	1.90548700	-0.79257200
H	5.54875300	0.22744100	-1.39366600
H	7.88446200	1.19084300	-1.10972900
H	7.58556900	1.26869100	0.64698400
C	2.73807100	-2.08341600	-0.17057900
C	2.51639000	-3.00244400	-1.25401100
H	2.30324200	-2.18371000	0.81710100
H	2.34926800	-4.02022000	-0.91095600
H	3.26941200	-2.94904500	-2.03752800
C	1.66980100	1.79094500	-0.50831000
C	1.52268400	1.71283300	-2.00901200
H	1.37452800	2.76782500	-0.12228500
H	1.01750000	1.02722800	-0.07579500
H	0.46597800	1.79989700	-2.26134800
H	1.90903000	0.76988100	-2.39577700

Cl	2.41917200	3.04296300	-2.86756300
Cl	7.87441700	-0.95948200	-0.11016600
Cl	0.88655600	-2.61430600	-2.24571600

^{4}IM$_{1\text{-}H}$

S	-4.40293600	-0.51668500	1.58817700
C	-3.01741400	3.21904000	0.52523000
C	-0.30689700	0.09151400	3.02134900
C	-1.97104600	-3.56382800	0.32654500
C	-4.35214700	-0.37462000	-2.41785600
C	-2.13540500	2.68334800	1.46638800
C	-1.44069900	3.45091200	2.47430600
C	-0.66571100	2.56850000	3.16510700
C	-0.90069900	1.26883200	2.58001700
C	-0.52849200	-1.18476500	2.50801000
C	0.06872500	-2.38585500	3.04009100
C	-0.41441700	-3.42220400	2.29745000
C	-1.29291300	-2.84269800	1.31022200
C	-2.76441700	-3.01417700	-0.67155200
C	-3.41761000	-3.76768200	-1.71510900
C	-4.06043400	-2.86448500	-2.50362500
C	-3.81283200	-1.56161700	-1.93128500
C	-4.23168900	0.87786600	-1.83049200
C	-4.86929600	2.07711300	-2.32381800
C	-4.51478600	3.08538200	-1.48364600
C	-3.64942900	2.50722700	-0.48230100
N	-1.79649800	1.36456700	1.54873700
N	-1.34741600	-1.48469800	1.45460100
N	-3.01527300	-1.66965600	-0.82204900
N	-3.50212100	1.15562200	-0.70135700
Fe	-2.36697100	-0.14702500	0.33412800
H	-3.20965000	4.28657800	0.57553500
H	0.40156800	0.17347600	3.83793900
H	-1.85155000	-4.64301600	0.32988400
H	-4.96032200	-0.44171500	-3.31465400
H	0.00238100	2.76016200	3.99396900
H	-1.54346900	4.51795700	2.62168400
H	0.76018300	-2.41482000	3.87128100
H	-0.20315800	-4.47872900	2.39610500
H	-3.37781500	-4.84402600	-1.81703700
H	-4.66096100	-3.04677000	-3.38486000
H	-5.50348500	2.12160800	-3.19919200
H	-4.79548500	4.12934600	-1.52790000
O	-0.91355400	0.18292500	-0.70467100

H	-4.63324600	0.75610700	1.97195600
H	-0.57288200	-0.66799500	-1.01762100
P	3.15768400	0.60407800	0.58923300
O	2.67529500	-0.46575400	-0.56654900
O	2.80973700	1.99308300	-0.12011100
O	2.62512700	0.32585200	1.93569800
O	4.74934700	0.58837100	0.49501400
C	5.43637800	0.62761000	-0.77585200
C	6.88012800	1.00776800	-0.53448500
H	4.97763900	1.38809000	-1.41834900
H	5.35270800	-0.35106500	-1.25578900
H	7.38259800	1.15245800	-1.49277200
H	6.94645200	1.92287700	0.05527800
C	2.09214800	-1.66258200	-0.25209500
C	2.04572900	-2.64512700	-1.30905300
H	1.94863800	-1.88642600	0.79827300
H	1.33777200	-3.44376000	-1.09972100
H	1.89113600	-2.21138100	-2.29563800
C	1.43671300	2.36742100	-0.40342400
C	1.44275100	3.36921200	-1.53451300
H	1.02410700	2.85231200	0.48905000
H	0.82140900	1.49353600	-0.64116400
H	0.42625200	3.72685700	-1.70607800
H	2.10028300	4.21273500	-1.31842700
Cl	2.02177100	2.65578100	-3.09920600
Cl	7.80368400	-0.26564700	0.35860000
Cl	3.72077800	-3.57044300	-1.51449100

^{4}TS$_{1\text{-OH}}$

S	4.35334400	0.26931600	1.10240900
C	3.46735000	-1.83211400	-2.32453900
C	1.79464600	-2.89077400	2.09004600
C	0.78408400	1.78980600	2.82095000
C	2.70525000	2.88391500	-1.48898200
C	3.03883800	-2.53305600	-1.20760500
C	3.13008800	-3.96645200	-1.05957100
C	2.67238900	-4.26287600	0.18714200
C	2.29436500	-3.01190800	0.80150500
C	1.42006100	-1.69510200	2.68774000
C	0.82568200	-1.59457000	3.99789700
C	0.52685300	-0.28230900	4.19647400
C	0.94523500	0.42577000	3.01275400
C	1.17455900	2.48670500	1.68645900
C	1.08567100	3.91820900	1.54130600

C	1.64265900	4.22992100	0.33603900
C	2.06667500	2.98914500	-0.26224300
C	3.11267300	1.69180200	-2.06987500
C	3.70963100	1.58763100	-3.37633400
C	3.90864000	0.26155600	-3.61663200
C	3.44198600	-0.45056100	-2.45494900
N	2.51240500	-1.97825800	-0.07058200
N	1.50693600	-0.45002000	2.11692800
N	1.75146300	1.94046200	0.56649600
N	2.96978100	0.43952300	-1.52114200
Fe	2.17282000	-0.01327200	0.26295300
H	3.87166400	-2.40462500	-3.15251300
H	1.67119200	-3.80003200	2.66871900
H	0.32656100	2.35647500	3.62456200
H	2.88589300	3.79899600	-2.04252400
H	2.59386900	-5.23042300	0.66506300
H	3.50839600	-4.63951800	-1.81756500
H	0.64556300	-2.43441700	4.65564700
H	0.04843300	0.17921600	5.04959800
H	0.67265200	4.58556800	2.28642200
H	1.77688400	5.20516500	-0.11332400
H	3.93035200	2.42988600	-4.01857900
H	4.32925500	-0.20838900	-4.49575000
O	0.47965900	-0.26496600	-0.43416000
H	4.06529400	0.12110800	2.41140000
H	0.13073700	0.59924100	-0.71329600
P	-3.00333400	0.25972500	1.03152900
O	-2.64242800	-0.55480900	-0.34949500
O	-4.59375800	0.41173800	0.87993800
O	-2.46544200	-0.36230500	2.25319500
O	-2.52047500	1.75681400	0.76218400
C	-3.16468600	2.65221800	-0.17001300
C	-2.11470000	3.43143100	-0.93112900
H	-3.76714200	3.35445700	0.41722100
H	-3.82715200	2.10825400	-0.84632100
H	-2.58710000	4.25478700	-1.47018000
H	-1.34438900	3.81850700	-0.26246900
C	-1.62791700	-1.47573600	-0.39087300
C	-1.45083300	-2.15985300	-1.64777100
H	-1.26862200	-1.84859600	0.55780200
H	-0.51201600	-2.70385500	-1.68549800
H	-1.59289100	-1.52200000	-2.51700500
C	-5.46133600	-0.73869900	0.98364100
C	-5.88207200	-1.25357500	-0.37920800

H	-6.32615700	-0.40782600	1.56227500
H	-4.95942500	-1.54248800	1.53500100
H	-6.52821700	-2.12494800	-0.25531900
H	-5.02380100	-1.52472200	-0.99356000
Cl	-6.83556600	-0.01787800	-1.30213300
Cl	-1.26562600	2.42001600	-2.17379500
Cl	-2.80092200	-3.52193500	-1.89505200

^{2}IM$_{1\text{-OH}}$

S	4.26197800	0.12134200	0.88216200
C	1.54472300	-2.79876700	2.22161700
C	1.16569000	1.98528900	2.82813300
C	2.47415100	2.73345700	-1.77237900
C	2.96705500	-2.04778800	-2.34672100
C	1.32339900	-1.55030300	2.78628000
C	0.91596000	-1.33294700	4.15348200
C	0.81877800	0.01186300	4.32216800
C	1.16464300	0.61798500	3.05816700
C	1.47528000	2.60056600	1.62228000
C	1.45168600	4.02835500	1.41272800
C	1.81686300	4.24255400	0.11714600
C	2.06416700	2.94503700	-0.46288300
C	2.71722000	1.48717900	-2.33193400
C	3.16900200	1.27293200	-3.68444300
C	3.31181900	-0.07155100	-3.84137000
C	2.94605700	-0.67958800	-2.58483200
C	2.62174500	-2.66395100	-1.15113400
C	2.64060000	-4.09116700	-0.93944300
C	2.22917600	-4.30597500	0.34044100
C	1.96120000	-3.01082700	0.91391400
N	1.46959100	-0.35018500	2.13278800
N	1.83876400	1.95720900	0.46562800
N	2.57311400	0.28440500	-1.67924100
N	2.19444500	-2.01964500	-0.01214800
Fe	2.12067700	-0.02685600	0.26340700
H	1.39457500	-3.67032500	2.85033900
H	0.89363300	2.62830800	3.65886900
H	2.62312700	3.60522300	-2.40107600
H	3.28432800	-2.68948100	-3.16260300
H	0.52798300	0.56130100	5.20753100
H	0.72392500	-2.11964500	4.87087500
H	1.19825700	4.75732200	2.17152200
H	1.92579000	5.18294800	-0.40734300
H	3.35165500	2.05955800	-4.40469100

H	3.63632700	-0.61728000	-4.71768800
H	2.93154000	-4.81967800	-1.68486600
H	2.11344900	-5.24661900	0.86252200
O	-0.11224400	-0.01549200	-0.38260000
H	4.28657700	-0.93753300	1.71929700
H	-0.30403500	0.64679800	-1.07043300
P	-2.85320400	0.29351900	1.10379100
O	-2.39641300	-0.44846800	-0.27595400
O	-4.44703500	0.34758700	0.89615500
O	-2.33169300	-0.33078700	2.33277500
O	-2.46906200	1.83860600	0.92914500
C	-3.12899600	2.74748300	0.02987500
C	-2.11015300	3.49264900	-0.80785400
H	-3.65791400	3.48079800	0.64993800
H	-3.86219200	2.23129200	-0.59349600
H	-2.57578000	4.36380200	-1.27162600
H	-1.24996700	3.80155500	-0.21234000
C	-1.08945600	-1.02416600	-0.37257400
C	-1.04154500	-1.82461500	-1.66753500
H	-0.88006000	-1.63969900	0.50381300
H	-0.03036500	-2.19789700	-1.82080800
H	-1.35093700	-1.20387700	-2.51055500
C	-5.24456300	-0.84403200	1.04806200
C	-5.53902100	-1.50515700	-0.28452500
H	-6.16634400	-0.52674600	1.54032200
H	-4.72775300	-1.55874100	1.69944500
H	-6.12288000	-2.41335600	-0.12121700
H	-4.62270800	-1.75011900	-0.82095400
Cl	-6.52353200	-0.43645400	-1.36966600
Cl	-1.45463600	2.49992600	-2.18824900
Cl	-2.14560400	-3.25729900	-1.62091900

^{4}IM$_{1\text{-}OH}$

S	-4.47398900	-0.11942400	0.90712100
C	-2.98145400	2.13869000	-2.30991600
C	-1.63380600	2.73961100	2.29537300
C	-1.09957200	-2.08033200	2.75601000
C	-2.54712900	-2.69220300	-1.81696200
C	-2.58658900	2.76196400	-1.12370500
C	-2.59776400	4.18659100	-0.86692700
C	-2.21960200	4.35596100	0.43317200
C	-1.97544800	3.03576600	0.97337200
C	-1.44317400	1.47002600	2.84401100
C	-1.02435400	1.21462900	4.20461200

C	-0.86856300	-0.13144500	4.32927300
C	-1.19307100	-0.71957600	3.04839300
C	-1.37205600	-2.69641200	1.53205900
C	-1.31054200	-4.11733800	1.26198700
C	-1.72844400	-4.29223600	-0.02755900
C	-2.04209400	-2.97787900	-0.54628000
C	-2.85258200	-1.42978900	-2.32899500
C	-3.33432800	-1.17979800	-3.66894200
C	-3.44951100	0.17069900	-3.80859000
C	-3.04244100	0.76458500	-2.55480200
N	-2.17852100	2.09616600	0.00028900
N	-1.55351100	0.27605500	2.17078100
N	-1.79499900	-2.03697800	0.41336200
N	-2.68709200	-0.23038600	-1.67430000
Fe	-2.24417700	0.01109500	0.29367500
H	-3.29201800	2.78694400	-3.12396900
H	-1.49956500	3.58121500	2.96820600
H	-0.77907800	-2.72478100	3.56890200
H	-2.71469700	-3.53537100	-2.48024800
H	-2.12463400	5.28198700	0.98517200
H	-2.87285300	4.94691100	-1.58637300
H	-0.84767900	1.98073300	4.94793500
H	-0.53879100	-0.68942600	5.19549300
H	-1.01168200	-4.87474900	1.97536900
H	-1.83374500	-5.21990100	-0.57536700
H	-3.54193300	-1.94823200	-4.40210800
H	-3.77106400	0.72826700	-4.67857700
O	0.19641200	0.02337100	-0.40013900
H	-4.30321700	0.05192800	2.23482800
H	0.39716800	-0.64468300	-1.07871200
P	2.89956900	-0.29739900	1.08900900
O	2.48168600	0.47346700	-0.28458500
O	4.50142800	-0.32528600	0.93711300
O	2.32815800	0.28150000	2.31825500
O	2.54809000	-1.84435400	0.85890300
C	3.24214200	-2.69753700	-0.06811400
C	2.24802800	-3.48973300	-0.89190500
H	3.83243900	-3.40604400	0.52494200
H	3.92554500	-2.12882000	-0.70267700
H	2.75112700	-4.32408600	-1.38324300
H	1.42376100	-3.85746300	-0.27938400
C	1.16339400	1.03240900	-0.40347100
C	1.12132900	1.81287900	-1.71092100
H	0.94820000	1.66520500	0.45940000

H	0.10872500	2.17983500	-1.87199800
H	1.43509900	1.18000300	-2.54327100
C	5.27228900	0.87828700	1.12397200
C	5.59639800	1.55783400	-0.19253400
H	6.18409900	0.57317400	1.64190600
H	4.72362000	1.57845600	1.76518300
H	6.15615700	2.47594100	-0.00250200
H	4.69324500	1.78870300	-0.75678200
Cl	6.63807700	0.51951000	-1.25421100
Cl	1.49532800	-2.51561500	-2.23584300
Cl	2.21935900	3.25140200	-1.68581000

IM$_{1\text{-}OH}$

O	2.43207500	-1.72443400	0.41057500
P	-0.16030600	-0.12711600	-0.11243100
O	-0.18505500	0.14244600	1.35728200
O	-1.04311800	0.90270800	-0.98160500
O	-0.75788100	-1.53502900	-0.57277800
O	1.26610700	-0.05721400	-0.81290300
C	2.50948100	-0.46129500	-0.08138300
H	2.17303600	-1.68910400	1.36891400
C	-0.81306700	2.32632600	-0.85509200
H	0.24923000	2.52964500	-0.69229900
C	3.56534100	-0.35463700	-1.17209700
H	3.45299600	-1.18639000	-1.86766600
H	3.48695500	0.59512400	-1.69887600
C	-1.65740100	2.88388700	0.28297600
H	-2.71847000	2.70092400	0.11007900
Cl	5.21878200	-0.44684600	-0.45542700
H	-1.35834800	2.45658600	1.23943100
Cl	-1.42423300	4.67657700	0.37614800
O	1.51588600	-1.34505400	2.88879000
H	0.79167500	-0.76785200	2.56350700
H	2.05587100	-0.79331800	3.46794900
C	-1.93912300	-2.08559800	0.05852300
C	-3.14560100	-1.79352000	-0.82311300
H	-2.06491800	-1.66793700	1.06057100
H	-3.01552000	-2.21916600	-1.81827400
Cl	-4.62037200	-2.54656500	-0.09368500
H	-3.32884100	-0.72234500	-0.90754200
H	2.65166300	0.29579400	0.69813500
H	-1.10738000	2.76249100	-1.81118500
H	-1.76626100	-3.15989900	0.14034200

TS_{BCEP}

O	-2.85957500	1.27484500	1.08371400
P	0.16382500	0.19051900	-0.11361500
O	0.27303600	-0.17965200	1.36747300
O	1.23282200	-0.71083200	-0.94299200
O	0.73663000	1.67015900	-0.38995100
O	-1.20638500	0.09366100	-0.76821700
C	-2.96730300	0.36949100	0.21992400
H	-2.24632400	0.90112700	2.09516500
C	1.06794100	-2.14168700	-0.96951200
H	0.00802900	-2.40854700	-0.91635000
C	-3.68164200	0.66969100	-1.06989900
H	-3.52986600	1.70991500	-1.35185200
H	-3.37274600	-0.00681700	-1.86114700
C	1.84755400	-2.76577900	0.18102600
H	2.91079500	-2.53607000	0.10555900
Cl	-5.45643100	0.41267600	-0.77799700
H	1.46452200	-2.42578100	1.14209500
Cl	1.67977700	-4.57021100	0.12114400
O	-1.48035100	0.53211600	2.90082300
H	-0.64720100	0.19727500	2.28120600
H	-1.81625200	-0.20641200	3.42542100
C	1.96957300	2.11856700	0.20806100
C	3.08839800	1.98931200	-0.81702100
H	2.19194800	1.54552200	1.11222300
H	2.87081500	2.57415900	-1.71095600
Cl	4.63332900	2.62628800	-0.11436200
H	3.25401500	0.94831200	-1.09139700
H	-2.90315800	-0.68349500	0.51038300
H	1.45900100	-2.48046900	-1.93127100
H	1.81904900	3.16475100	0.48354000

P_{BCEP}

O	3.34853300	-1.37034600	1.44533200
P	-0.22621800	-0.17094400	-0.01667300
O	-0.48267400	0.20519300	1.50501300
O	-1.36578100	0.67915600	-0.78719600
O	-0.74701700	-1.67199700	-0.24591600
O	1.15861300	0.00992600	-0.52147300
C	3.55870500	-0.59542300	0.52855100
H	2.20659600	-0.77223900	2.65746400
C	-1.21866000	2.11056200	-0.91624000
H	-0.16156300	2.38907800	-0.88873200

C	4.04266500	-1.06185100	-0.82059600
H	4.37482200	-2.09830400	-0.77579200
H	3.22389200	-0.93686400	-1.53232600
C	-2.00239800	2.79777600	0.19415800
H	-3.05860800	2.53026500	0.15204500
Cl	5.42015800	-0.02456200	-1.36818600
H	-1.60072200	2.54800800	1.17524800
Cl	-1.88964900	4.59430600	-0.00938100
O	1.45126700	-0.35857800	3.13616600
H	0.27903700	-0.02088700	2.13656000
H	1.81234200	0.41443400	3.58748400
C	-2.03010200	-2.12575900	0.23716200
C	-3.01412500	-2.12843900	-0.92440500
H	-2.38301600	-1.49400000	1.05616900
H	-2.65983700	-2.76482200	-1.73558200
Cl	-4.60600200	-2.79136200	-0.36854300
H	-3.18751700	-1.11945000	-1.29605500
H	3.41005100	0.49146800	0.62956700
H	-1.62202600	2.37525400	-1.89565600
H	-1.87681900	-3.13831300	0.61638900

Cartesian coordinates of reaction species for TEP C_1-hydroxylation and O-dealkylation

^{2}RC

S	-3.73011300	0.02446400	1.74043800
C	-1.86259800	3.39462600	0.34973400
C	0.71095700	0.00246900	2.64494000
C	-1.82994500	-3.43417800	0.38507500
C	-4.10299100	-0.04429600	-2.20402800
C	-0.95926900	2.76428100	1.20205900
C	-0.04939000	3.45855000	2.08040100
C	0.68806900	2.50338000	2.71196200
C	0.21640600	1.23012600	2.22702500
C	0.23427700	-1.23489800	2.23088600
C	0.71346000	-2.49796700	2.73503000
C	-0.01954900	-3.46563600	2.11843600
C	-0.93076500	-2.78818600	1.22798100
C	-2.67127900	-2.81020300	-0.52382500
C	-3.55028700	-3.50258100	-1.43476500
C	-4.16818800	-2.54878600	-2.18223200
C	-3.67848600	-1.27380800	-1.71617300
C	-3.71540600	1.19092300	-1.70944300
C	-4.22003500	2.45726900	-2.18126400

C	-3.60655500	3.42489300	-1.44853600
C	-2.71440200	2.75414000	-0.53482500
N	-0.79043300	1.40999200	1.31279400
N	-0.76451700	-1.43261000	1.31498000
N	-2.76862300	-1.45281800	-0.70628800
N	-2.80764400	1.38879200	-0.69428100
Fe	-1.66986500	-0.02338600	0.20442100
H	-1.88778200	4.47969100	0.36791200
H	1.54205400	0.00910400	3.33971700
H	-1.85732100	-4.51857900	0.42542200
H	-4.82548200	-0.05232600	-3.01380200
H	1.47858100	2.62848100	3.43937000
H	0.00782200	4.53406700	2.18355600
H	1.50427900	-2.60700000	3.46454200
H	0.04083300	-4.53914100	2.23892000
H	-3.66346000	-4.57741400	-1.48392300
H	-4.89796100	-2.67686300	-2.97054200
H	-4.95268900	2.57123000	-2.96910900
H	-3.72811500	4.49807200	-1.51244500
O	-0.51854700	0.00429900	-0.94787000
H	-3.60780500	1.30199100	2.15790300
H	1.42620600	-1.44615100	-0.83575900
P	4.02709900	0.05784500	-0.03422900
O	3.41381700	-0.94134500	-1.14185600
O	3.83070700	1.50839000	-0.70378900
O	3.52655200	-0.13670200	1.34527500
O	5.61534100	-0.11711800	-0.16029200
C	6.28668700	-0.03073400	-1.43918700
C	7.76649000	-0.26760900	-1.20315800
H	6.10356000	0.96012300	-1.86763000
H	5.86307600	-0.78427300	-2.11184200
H	8.30900900	-0.20851100	-2.15239500
H	8.17486600	0.48461900	-0.52251000
H	7.93652400	-1.25602700	-0.76706400
C	2.40509700	-1.93359600	-0.80036700
C	2.49701000	-3.05818900	-1.81424400
H	2.58120000	-2.28987200	0.21770900
H	1.72677900	-3.80707800	-1.60172700
H	2.33645100	-2.68141200	-2.82859900
H	3.47571900	-3.54633200	-1.77542900
C	2.48941800	2.04211500	-0.86971100
C	2.55758700	3.15999000	-1.89267100
H	2.14569200	2.41033800	0.10161500
H	1.81006700	1.24896300	-1.19797700

H	1.56501700	3.60403200	-2.01999800
H	3.24993500	3.94311600	-1.56908500
H	2.89241500	2.77983800	-2.86213100

^{4}RC

S	-3.71365200	0.01686100	1.75169000
C	-1.86615500	3.39622800	0.35188700
C	0.71308900	-0.00141400	2.63163800
C	-1.84028600	-3.43277000	0.37949800
C	-4.12382000	-0.03828900	-2.19459600
C	-0.95703400	2.76497100	1.19749900
C	-0.04113800	3.45676500	2.07163000
C	0.69658200	2.49925100	2.69928400
C	0.21935800	1.22729100	2.21590500
C	0.23493900	-1.23838100	2.21755300
C	0.71892900	-2.50257100	2.71521200
C	-0.01694800	-3.46920400	2.10050100
C	-0.93474500	-2.78952900	1.21805200
C	-2.68554400	-2.80652300	-0.52399200
C	-3.57198900	-3.49747600	-1.42872700
C	-4.19269900	-2.54312800	-2.17317100
C	-3.69745600	-1.26898300	-1.71116400
C	-3.73348800	1.19528500	-1.69835600
C	-4.24553300	2.46237800	-2.15931000
C	-3.62726100	3.42818500	-1.42826200
C	-2.72447300	2.75613900	-0.52636200
N	-0.79093700	1.41063700	1.30585700
N	-0.76917900	-1.43481800	1.30788300
N	-2.78075000	-1.44859900	-0.70784900
N	-2.81464300	1.39066700	-0.69213900
Fe	-1.68133100	-0.02048500	0.19965300
H	-1.89260200	4.48122200	0.37195000
H	1.54716000	0.00398100	3.32284300
H	-1.86956300	-4.51718000	0.41855900
H	-4.85266200	-0.04397900	-2.99866700
H	1.49084200	2.62177800	3.42303800
H	0.01930300	4.53206800	2.17513200
H	1.51478500	-2.61272700	3.43906200
H	0.04549300	-4.54302500	2.21708200
H	-3.68792400	-4.57209900	-1.47603500
H	-4.92802800	-2.67035700	-2.95642700
H	-4.98635500	2.57799400	-2.93921900
H	-3.75223400	4.50131900	-1.48594900
O	-0.51003500	-0.01070700	-0.93503200

H	-3.59525400	1.29518100	2.16769500
H	1.44802600	-1.46153900	-0.82010300
P	4.03739700	0.06083600	-0.03199300
O	3.43017800	-0.94292200	-1.13897200
O	3.83682400	1.50874800	-0.70576900
O	3.53440300	-0.13274300	1.34676700
O	5.62662400	-0.10822400	-0.15363000
C	6.30077300	-0.02261200	-1.43113900
C	7.78045100	-0.25646800	-1.19139300
H	6.11681200	0.96727300	-1.86147200
H	5.88006200	-0.77791000	-2.10364100
H	8.32502400	-0.19757200	-2.13946400
H	8.18588700	0.49738200	-0.51078600
H	7.95130800	-1.24401500	-0.75365300
C	2.43008400	-1.94308700	-0.79560800
C	2.52064800	-3.06056200	-1.81746400
H	2.61630700	-2.30492400	0.21870400
H	1.75722200	-3.81592300	-1.60333600
H	2.34899300	-2.67828300	-2.82793600
H	3.50282800	-3.54245500	-1.79017300
C	2.49378400	2.03619500	-0.87785400
C	2.55922100	3.14619900	-1.90952500
H	2.14738700	2.41072200	0.09008400
H	1.81787900	1.23791000	-1.20065000
H	1.56525400	3.58573800	-2.04153800
H	3.24835700	3.93432800	-1.59127500
H	2.89663400	2.75963000	-2.87554500

$^{2}TS_{1\text{-}H}$

S	3.67230800	0.06717300	-1.40738100
C	1.77558000	3.40274200	0.05023900
C	-0.43616200	0.40929500	-3.04224300
C	1.52509000	-3.39375700	-0.79754000
C	3.40952500	-0.41411900	2.51680900
C	1.04087900	2.91588900	-1.02920600
C	0.33843400	3.74675200	-1.97770500
C	-0.29507900	2.90374900	-2.84043900
C	0.02953800	1.56213300	-2.41958100
C	-0.12239700	-0.89315900	-2.67681500
C	-0.55092600	-2.06465400	-3.40356400
C	0.02123300	-3.13506500	-2.78982500
C	0.78556600	-2.61426600	-1.68055500
C	2.23277200	-2.91402700	0.29813700
C	2.93535700	-3.73790500	1.25146700

C	3.43885000	-2.89733200	2.19651400
C	3.05777700	-1.55948500	1.81235300
C	3.13476700	0.89242400	2.13549000
C	3.57541500	2.06841900	2.85212500
C	3.13306700	3.14469700	2.14883700
C	2.41209500	2.62671800	1.00813100
N	0.85080000	1.59186800	-1.32428800
N	0.67447700	-1.24895400	-1.61971300
N	2.32417300	-1.59112600	0.65202300
N	2.43272400	1.25401500	1.01532300
Fe	1.49825600	-0.00863100	-0.28055000
H	1.83816200	4.48106800	0.16054300
H	-1.09656900	0.53766000	-3.89314800
H	1.53328900	-4.46617500	-0.96611400
H	3.98325700	-0.55350800	3.42783400
H	-0.92515500	3.14984100	-3.68476100
H	0.33581600	4.82876900	-1.96777200
H	-1.19948900	-2.04824500	-4.26884200
H	-0.05522300	-4.18274800	-3.04890100
H	3.01801800	-4.81516800	1.19374800
H	4.02312200	-3.14291500	3.07343800
H	4.15314900	2.05231900	3.76681600
H	3.26902100	4.19560600	2.36769900
O	0.11913000	0.07013600	0.78720500
H	3.73411500	1.39972100	-1.60914200
H	-0.69485500	-0.81843900	0.72714300
P	-3.69524800	-0.07151800	0.04587300
O	-2.83004200	-0.89801000	1.16145900
O	-3.68343600	1.42566900	0.61606900
O	-3.26937100	-0.29763000	-1.35015500
O	-5.19994700	-0.51551000	0.35277400
C	-5.78487800	-0.38837100	1.67295500
C	-7.18885400	-0.95895100	1.61198600
H	-5.79221400	0.66982800	1.95203500
H	-5.16177600	-0.93447000	2.38907000
H	-7.66638000	-0.87617700	2.59374500
H	-7.79624600	-0.41467000	0.88359700
H	-7.16691300	-2.01373400	1.32425700
C	-1.69802900	-1.65993100	0.80650600
C	-1.35978000	-2.63995000	1.89652300
H	-1.77893000	-2.06317600	-0.20532100
H	-0.43877000	-3.17264000	1.64628000
H	-1.21640400	-2.12258200	2.84949500
H	-2.16505700	-3.37594700	2.02282400

C	-2.47087100	2.23252900	0.51744400
C	-2.62794000	3.41172800	1.45691600
H	-2.36780800	2.55796700	-0.52194400
H	-1.60166900	1.62155900	0.78102100
H	-1.74484100	4.05482800	1.38514900
H	-3.51078900	4.00542200	1.20072900
H	-2.72377800	3.07395700	2.49287000

$^{4}TS_{1\text{-}H}$

S	-3.63843800	-0.06220000	1.51643900
C	-2.02854200	3.35755100	-0.18480100
C	0.47705600	0.65756600	2.94642800
C	-1.35186100	-3.35596200	0.97366200
C	-3.42210300	-0.66678400	-2.47012300
C	-1.20947300	2.97463500	0.87775700
C	-0.52557600	3.89171900	1.75881400
C	0.19452900	3.12897600	2.62819100
C	-0.06241900	1.75151800	2.27888700
C	0.21040700	-0.67984500	2.67126900
C	0.71907700	-1.78427400	3.44901700
C	0.17935200	-2.91479400	2.91694700
C	-0.64088400	-2.49300800	1.80563300
C	-2.08708100	-2.97592600	-0.14080100
C	-2.75209200	-3.88842800	-1.03980800
C	-3.30124200	-3.13185900	-2.02860400
C	-2.98925000	-1.75578800	-1.72286500
C	-3.24472400	0.66979400	-2.13488100
C	-3.80006300	1.78045200	-2.87382400
C	-3.43462800	2.91244100	-2.21490100
C	-2.64164600	2.49575200	-1.08185600
N	-0.92107300	1.68323100	1.21447800
N	-0.60328600	-1.13294100	1.66946800
N	-2.24737500	-1.67981400	-0.57126800
N	-2.54717800	1.12321000	-1.04428500
Fe	-1.50246600	-0.00047700	0.26136100
H	-2.17790700	4.42215800	-0.33742600
H	1.15885600	0.86423500	3.76447500
H	-1.30358400	-4.41677000	1.19984300
H	-3.99652000	-0.87957000	-3.36647400
H	0.83742500	3.45125100	3.43648200
H	-0.59494100	4.97017500	1.70490400
H	1.39678900	-1.68767500	4.28609600
H	0.31734200	-3.94122100	3.22984800
H	-2.78080600	-4.96317200	-0.91825400

H	-3.87785100	-3.45734100	-2.88429600
H	-4.39602500	1.68621600	-3.77194400
H	-3.66440000	3.94041800	-2.46226100
O	-0.08115100	0.07080400	-0.76324400
H	-4.00546800	1.22377900	1.33820300
H	0.71114700	-0.82635200	-0.71187200
P	3.69867500	-0.01511700	-0.05411200
O	2.85785100	-0.88758900	-1.14781100
O	3.68132400	1.46155000	-0.67759600
O	3.26372000	-0.19168200	1.34684400
O	5.21434700	-0.45045200	-0.32302400
C	5.81285700	-0.37470000	-1.64018200
C	7.22314400	-0.92396200	-1.53859400
H	5.81128100	0.67000700	-1.96669700
H	5.20509600	-0.95975200	-2.33858300
H	7.71107000	-0.87872700	-2.51769500
H	7.81526400	-0.34013900	-0.82843800
H	7.21093400	-1.96510300	-1.20430200
C	1.73848500	-1.67093800	-0.78214300
C	1.42987300	-2.67225600	-1.86165100
H	1.83113700	-2.06162200	0.23366800
H	0.52794900	-3.23320100	-1.60329300
H	1.26381700	-2.16918000	-2.81890700
H	2.25788500	-3.38284900	-1.98792600
C	2.46035500	2.25865600	-0.62946200
C	2.61379400	3.39151700	-1.62528600
H	2.34517700	2.63507800	0.39152100
H	1.59921700	1.62597900	-0.86714100
H	1.72582100	4.03110600	-1.59119700
H	3.49059000	4.00396400	-1.39354000
H	2.71928800	3.00352900	-2.64257600

$^{2}IM_{1-H}$

S	-3.53063700	-0.04521100	1.63134000
C	-1.62917500	3.43194800	0.33843200
C	0.48982400	-0.07422500	2.92889200
C	-1.83547300	-3.41375200	0.30205700
C	-3.75897900	0.09073300	-2.43624900
C	-0.90912100	2.75933200	1.31684800
C	-0.09748700	3.40837100	2.31597800
C	0.51039500	2.42267000	3.03401300
C	0.06509700	1.16820500	2.48249800
C	0.07303600	-1.28826800	2.40561100
C	0.45627000	-2.57409500	2.93152000

C	-0.20997600	-3.51337900	2.20370300
C	-0.99543700	-2.80522300	1.22405400
C	-2.59456500	-2.74110100	-0.64474900
C	-3.41162900	-3.38820000	-1.64067100
C	-3.94047200	-2.40367800	-2.41827700
C	-3.45429000	-1.15060800	-1.89689500
C	-3.30581500	1.30382600	-1.94120900
C	-3.68475800	2.59121600	-2.47436200
C	-3.09628700	3.53141800	-1.68730100
C	-2.35128700	2.82062800	-0.67480500
N	-0.81463500	1.39651900	1.45148400
N	-0.79204100	-1.45278900	1.35113900
N	-2.64725700	-1.37958700	-0.81143100
N	-2.48217500	1.46962800	-0.85956200
Fe	-1.65671000	0.00850800	0.25686300
H	-1.61534400	4.51648500	0.36284300
H	1.20358800	-0.09779200	3.74406000
H	-1.89923900	-4.49660600	0.31901900
H	-4.41459700	0.11401100	-3.30011900
H	1.20568500	2.51652200	3.85743600
H	-0.00540600	4.48051700	2.42972700
H	1.13981300	-2.71817000	3.75738500
H	-0.18877600	-4.58985700	2.31125600
H	-3.54331600	-4.45883500	-1.72494900
H	-4.59772900	-2.49822300	-3.27252100
H	-4.32712900	2.73469800	-3.33309800
H	-3.15273800	4.60918000	-1.76487600
O	-0.26446800	0.01247100	-0.86347000
H	-3.07869700	0.78620800	2.59235600
H	0.36958700	-0.70522700	-0.64703900
P	3.93511800	-0.03342800	-0.03432100
O	3.12989900	-0.99601900	-1.10081100
O	3.73212900	1.41982400	-0.66797000
O	3.56014500	-0.25690600	1.37532000
O	5.46771000	-0.32702600	-0.36803500
C	6.01158700	-0.17263500	-1.70494600
C	7.47876700	-0.55161500	-1.65084300
H	5.87376200	0.86607700	-2.01961300
H	5.45415400	-0.82271100	-2.38747000
H	7.92547700	-0.44653900	-2.64481000
H	8.02048800	0.09648600	-0.95654600
H	7.60045000	-1.58782800	-1.32341400
C	2.17485000	-1.90432100	-0.68268000
C	1.77402600	-2.89035000	-1.71983000

H	2.17472100	-2.12849900	0.38012600
H	0.91650300	-3.47105800	-1.36995600
H	1.49851100	-2.38922700	-2.65427100
H	2.58978800	-3.59459200	-1.95368600
C	2.44358500	2.10005000	-0.54069300
C	2.48591500	3.33048800	-1.42386800
H	2.30451500	2.36322000	0.51147800
H	1.63956100	1.42173200	-0.84287600
H	1.53774200	3.86973700	-1.33427000
H	3.29834400	4.00088600	-1.12767900
H	2.62596300	3.05322900	-2.47259000

^{4}IM$_{1\text{-}H}$

S	-3.50850100	-0.08716000	1.68012900
C	-1.68919100	3.43414700	0.32186000
C	0.51379600	-0.03478300	2.89259800
C	-1.81967500	-3.41206600	0.32629200
C	-3.81307900	0.05299200	-2.41222100
C	-0.94705700	2.77546600	1.29368200
C	-0.12637700	3.43751800	2.27648300
C	0.50556900	2.46127400	2.98661600
C	0.06556400	1.19949900	2.44756100
C	0.10217600	-1.25839100	2.38602000
C	0.50995700	-2.53722100	2.91210700
C	-0.15962600	-3.48749400	2.20197000
C	-0.97091900	-2.79213300	1.23333700
C	-2.59980200	-2.75288100	-0.61231300
C	-3.42146400	-3.41648600	-1.59341200
C	-3.97052900	-2.44432900	-2.37261900
C	-3.49213800	-1.18209700	-1.86676200
C	-3.36373700	1.27185000	-1.93010200
C	-3.76119000	2.55202200	-2.46506500
C	-3.17601300	3.50297800	-1.68836800
C	-2.41281600	2.80755400	-0.67987700
N	-0.83549900	1.41513700	1.43066400
N	-0.78058900	-1.43844700	1.35123600
N	-2.67156100	-1.39225500	-0.78896600
N	-2.52884700	1.45221400	-0.85688000
Fe	-1.68434800	0.01647500	0.25561500
H	-1.68931600	4.51884800	0.33976900
H	1.24483500	-0.04383100	3.69254600
H	-1.87131800	-4.49552900	0.34756800
H	-4.47815400	0.06546900	-3.26900400
H	1.21444400	2.56603400	3.79703900

H	-0.04438100	4.51102500	2.38470800
H	1.21156800	-2.66906200	3.72471500
H	-0.12418000	-4.56313400	2.31399100
H	-3.54136000	-4.48920200	-1.66821000
H	-4.63599300	-2.55316500	-3.21874800
H	-4.41387200	2.68407200	-3.31783200
H	-3.24601200	4.57956200	-1.77090700
O	-0.28902200	-0.00157700	-0.86570700
H	-3.02605400	0.70589700	2.65866900
H	0.33331700	-0.72151600	-0.64117800
P	3.95021900	-0.02141900	-0.03759400
O	3.18438200	-0.99139700	-1.12076400
O	3.74266800	1.42969400	-0.67717100
O	3.54700300	-0.24556600	1.36450500
O	5.49667900	-0.28952600	-0.33153700
C	6.06632900	-0.13867400	-1.65754300
C	7.53924200	-0.48789100	-1.56818700
H	5.91543700	0.89374200	-1.98694000
H	5.53657400	-0.80661300	-2.34488000
H	8.00511300	-0.38480700	-2.55355800
H	8.05319700	0.17828000	-0.86986600
H	7.67415100	-1.51786600	-1.22652800
C	2.25069800	-1.93631500	-0.72283500
C	1.89272600	-2.91998400	-1.77788500
H	2.25395900	-2.18007900	0.33561100
H	1.05485600	-3.53699400	-1.44183300
H	1.60257400	-2.41556200	-2.70622800
H	2.73314500	-3.59209200	-2.01983100
C	2.43977900	2.08590400	-0.58605400
C	2.47479400	3.30311000	-1.48793900
H	2.27468900	2.36329400	0.45876500
H	1.65394700	1.38813900	-0.89169400
H	1.51568700	3.82650200	-1.42382400
H	3.26952900	3.99280600	-1.18804600
H	2.63909100	3.01228700	-2.52945700

^{4}TS$_{1\text{-OH}}$

S	3.82836900	0.33739500	0.92900700
C	2.62622900	0.74190100	-2.96901500
C	2.18467200	-3.30876600	-0.35836900
C	0.58656600	-0.70810900	3.40512100
C	1.16154800	3.35816100	0.84152700
C	2.59054200	-0.59431100	-2.59866000
C	2.97764800	-1.68828100	-3.45727200

C	2.86026000	-2.82663000	-2.72107000
C	2.39821600	-2.43080200	-1.41150800
C	1.75564100	-2.94196900	0.91025500
C	1.47631600	-3.87413100	1.97466800
C	1.01990100	-3.14398200	3.02969800
C	1.02292100	-1.76299800	2.61487600
C	0.55556200	0.62144700	3.01274800
C	0.17339800	1.71488600	3.87188200
C	0.34355600	2.85916400	3.15526900
C	0.82482100	2.47001400	1.85271900
C	1.65154500	2.99467900	-0.40603700
C	1.94703100	3.92592700	-1.46563200
C	2.34898400	3.18973300	-2.53877900
C	2.30573100	1.80604500	-2.13846400
N	2.22813200	-1.07191500	-1.36742100
N	1.49492600	-1.66225900	1.33016600
N	0.92393300	1.10275800	1.77987900
N	1.88874100	1.71169100	-0.83354400
Fe	1.61046500	0.01947700	0.21261600
H	2.94257200	0.97430200	-3.98034800
H	2.36020100	-4.36372400	-0.54138100
H	0.24827700	-0.94132000	4.40891900
H	1.03430000	4.41697200	1.04067000
H	3.06950900	-3.84635600	-3.01646400
H	3.30453500	-1.57840900	-4.48284500
H	1.60532400	-4.94600400	1.90187200
H	0.69632000	-3.49178300	4.00175900
H	-0.16973900	1.60202500	4.89158600
H	0.17016300	3.88115100	3.46516600
H	1.84724300	5.00022900	-1.38385500
H	2.64887600	3.53531400	-3.51929400
O	-0.10074500	-0.21540100	-0.36950100
H	3.92243800	-0.74134700	1.73278500
H	-0.69817500	0.27926800	0.21682300
P	-3.85162700	-0.06254000	0.13765100
O	-3.37312100	-1.06543700	-1.06893100
O	-5.25630000	0.44301000	-0.44350900
O	-3.83742200	-0.66148600	1.48642700
O	-2.92142900	1.22902800	-0.01291600
C	-2.67787800	1.84961100	-1.31255900
C	-2.38545600	3.31847200	-1.08004600
H	-3.55906800	1.71654600	-1.94625800
H	-1.82562800	1.33147300	-1.76004500
H	-2.16382400	3.80353000	-2.03646200

H	-3.24499600	3.81907400	-0.62549200
C	-2.23064900	-1.82742700	-0.88450600
C	-1.71484500	-2.50182600	-2.09402000
H	-2.04014600	-2.16295300	0.12750400
H	-0.73534600	-2.93483800	-1.87875800
H	-1.60413400	-1.79233700	-2.92166400
C	-6.48209400	-0.27733700	-0.14078400
C	-6.79117700	-1.31330400	-1.20904000
H	-7.25606700	0.49327200	-0.09941900
H	-6.38903100	-0.72938300	0.85096700
H	-7.75130800	-1.79319300	-0.99094500
H	-6.01884800	-2.08604200	-1.24476800
H	-1.51923300	3.44092500	-0.42465000
H	-6.85530800	-0.84483200	-2.19526400
H	-2.38242600	-3.30880500	-2.44118100

$^{2}IM_{1\text{-}OH}$

S	3.63467800	1.14864000	-0.74954800
C	0.94405100	2.88678800	1.83849600
C	0.03145800	1.82296700	-2.79635200
C	2.24354700	-2.43896600	-2.19230500
C	3.13435800	-1.38547800	2.44536400
C	0.50922000	2.96468300	0.52205500
C	-0.18814100	4.09958900	-0.03353800
C	-0.44839700	3.80201600	-1.33727500
C	0.08883800	2.48466900	-1.57647200
C	0.55389700	0.56002800	-3.03374100
C	0.51358700	-0.11674400	-4.30593700
C	1.14060700	-1.31194700	-4.13373000
C	1.56426500	-1.36781000	-2.75565500
C	2.66732900	-2.52162300	-0.87309100
C	3.37635200	-3.65226700	-0.32116000
C	3.62690300	-3.36017100	0.98366100
C	3.07043100	-2.05133900	1.22908300
C	2.60823100	-0.12279400	2.68243800
C	2.68409600	0.56823200	3.94635300
C	2.06481100	1.76766700	3.77386600
C	1.61312700	1.81047400	2.40340800
N	0.66557500	1.98837000	-0.43160900
N	1.19140200	-0.22046300	-2.09561900
N	2.48117100	-1.56026700	0.08916300
N	1.94155700	0.64342400	1.75689200
Fe	1.65995700	0.25450100	-0.19563000
H	0.75130300	3.74300800	2.47718900

H	-0.45130200	2.33184700	-3.62426900
H	2.46554500	-3.28143500	-2.83948700
H	3.63963100	-1.88322300	3.26682500
H	-0.94870000	4.40780600	-2.08158900
H	-0.43069200	5.00119700	0.51390800
H	0.05566000	0.28248200	-5.20117100
H	1.30605500	-2.09687800	-4.85991700
H	3.64067700	-4.54300900	-0.87591100
H	4.14053200	-3.95990400	1.72357400
H	3.15486600	0.17338000	4.83709800
H	1.92256600	2.56298700	4.49384400
O	-0.48846900	-0.71728100	0.20291900
H	3.22066000	1.86427000	-1.81681500
H	-0.88497400	-0.75920300	-0.69107800
P	-3.51481100	-0.72233100	-0.17156300
O	-2.61711700	-0.86239400	1.17124100
O	-4.25277200	0.68164900	0.04600900
O	-2.72284600	-0.91498900	-1.41366800
O	-4.72171500	-1.75751200	-0.03180900
C	-5.59386600	-1.76238800	1.12915000
C	-6.54817700	-2.93172900	0.98414300
H	-6.12817000	-0.80815700	1.16650000
H	-4.97898500	-1.85487400	2.03076900
H	-7.22770300	-2.96325500	1.84184000
H	-7.14399400	-2.83373500	0.07265800
H	-6.00072700	-3.87723000	0.93972700
C	-1.29784400	-1.48246600	1.04809600
C	-0.71541800	-1.54729500	2.43820600
H	-1.44979000	-2.48154200	0.62093900
H	0.26737700	-2.02081000	2.40550700
H	-0.60860700	-0.53880300	2.84304800
H	-1.37584600	-2.12816400	3.08659500
C	-3.46662400	1.90782200	0.01491800
C	-4.41730600	3.07315900	0.20068000
H	-2.94458200	1.96327200	-0.94439900
H	-2.72020300	1.86327200	0.81396100
H	-3.85215700	4.00993300	0.17561100
H	-5.16487700	3.09651700	-0.59719100
H	-4.93571300	3.00625900	1.16151800

^{4}IM$_{1\text{-}OH}$

S	3.96392600	-0.24888700	1.67629000
C	3.62695700	0.45114700	-2.52698200
C	1.63399400	-3.38796300	-0.35854500

C	-0.17681800	-0.49787600	3.08570600
C	1.74998700	3.34517600	0.87053600
C	3.22413800	-0.84262900	-2.23719400
C	3.55839800	-1.99228800	-3.03780300
C	2.99494200	-3.07259300	-2.43031600
C	2.31744500	-2.58356600	-1.25683200
C	0.98279300	-2.92798600	0.77690400
C	0.25906800	-3.76894300	1.69115700
C	-0.24656000	-2.95546500	2.66186100
C	0.16548700	-1.61648900	2.34158400
C	0.17456300	0.80132600	2.76181400
C	-0.20167000	1.95789300	3.53231500
C	0.33625600	3.03931700	2.90635700
C	1.04315400	2.54457700	1.75284100
C	2.44553200	2.87978600	-0.23526500
C	3.15390900	3.72642900	-1.15844400
C	3.68595400	2.91177700	-2.11163500
C	3.30093700	1.56735500	-1.77235900
N	2.45723600	-1.21744600	-1.15742200
N	0.92018600	-1.61187600	1.18664400
N	0.92566200	1.17528100	1.66927300
N	2.54765800	1.56237800	-0.62003700
Fe	1.91220100	-0.04772400	0.39782700
H	4.23279300	0.60314300	-3.41399900
H	1.60474000	-4.45414800	-0.55763000
H	-0.78448600	-0.64912000	3.97090500
H	1.75774400	4.41395600	1.05617900
H	3.02839900	-4.11084500	-2.73321400
H	4.15007900	-1.95959200	-3.94308000
H	0.15741800	-4.84154800	1.59007600
H	-0.84945300	-3.22326800	3.51942800
H	-0.80727000	1.92751200	4.42796500
H	0.26934300	4.08268400	3.18449200
H	3.22127600	4.80318800	-1.07694000
H	4.28003800	3.18179700	-2.97462600
O	-0.93865300	0.57651300	-0.99905400
H	3.67047100	-1.46160100	2.19020500
H	-1.09006200	0.70726300	-0.04504900
P	-3.76744400	0.29699800	-0.06384800
O	-3.08762400	-0.34927000	-1.38113100
O	-5.13704500	-0.52898700	0.10133100
O	-2.85496900	0.33580800	1.10670800
O	-4.31919800	1.73129100	-0.49304900
C	-5.11624300	1.91892200	-1.69013100

C	-5.35552700	3.40706600	-1.85633900
H	-6.05704500	1.37139000	-1.57203700
H	-4.57157000	1.50179500	-2.54320400
H	-5.96032200	3.58759100	-2.75088800
H	-5.88641600	3.81303600	-0.99086800
C	-1.62648200	-0.56572900	-1.37947400
C	-1.25064100	-0.93241700	-2.79650800
H	-1.44684900	-1.38813100	-0.67789700
H	-0.18601600	-1.17342800	-2.83572100
H	-1.44809000	-0.08450300	-3.45756500
C	-5.09989500	-1.91075700	0.54254300
C	-5.35414200	-2.85064400	-0.62392300
H	-5.88307500	-1.99615400	1.30095000
H	-4.14075600	-2.11503000	1.02972200
H	-5.37179900	-3.88757000	-0.27145500
H	-4.57083400	-2.75030600	-1.37931600
H	-4.40755200	3.94118900	-1.96351400
H	-6.31750900	-2.63058400	-1.09295900
H	-1.83009000	-1.79443500	-3.13523900

$IM_{1\text{-}OH}$

O	2.70365100	-0.80539600	-0.27738000
P	-0.31666600	0.05315000	-0.09410300
O	-0.18753600	-0.11689300	1.38901100
O	-1.52964700	1.00094400	-0.55057700
O	-0.60889800	-1.28415900	-0.91185700
O	0.94338300	0.69853800	-0.82083400
C	2.33415800	0.51182600	-0.30192200
H	2.49781400	-1.18604000	0.61238600
C	-1.61242600	2.36317900	-0.05467400
H	-0.60212400	2.77364500	0.04801800
C	3.20030900	1.29289000	-1.26654800
H	3.15307700	0.82360100	-2.25218400
H	2.86415800	2.32917900	-1.34538000
C	-2.38238100	2.43320800	1.25335100
H	-3.39047000	2.02761100	1.12784400
H	-1.87024200	1.86275600	2.03146100
O	1.92559700	-1.59346800	2.19664700
H	1.06913100	-1.11850900	2.08834300
H	2.38740600	-1.12833100	2.90498500
C	-1.47524500	-2.31952000	-0.37373000
C	-2.90539500	-2.13817800	-0.85295800
H	-1.40747400	-2.31016900	0.71817500
H	-2.94788300	-2.13280400	-1.94550700

H	-3.32563800	-1.19755600	-0.48789800
H	2.32262200	0.95466600	0.70235100
H	-2.11920900	2.91812000	-0.84823000
H	-1.04821600	-3.25677600	-0.73810500
H	4.23578400	1.27329000	-0.91746100
H	-2.46965300	3.47593900	1.57727000
H	-3.52560500	-2.96282400	-0.48571300

TS_{DEP}

O	2.99989400	-0.76766900	0.28488200
P	-0.34350200	0.00716200	-0.15947100
O	-0.22570700	0.06124100	1.37188900
O	-1.64673300	0.86051900	-0.60381900
O	-0.74930900	-1.46982900	-0.64560000
O	0.86806200	0.45587300	-0.96423500
C	2.81441700	0.30662400	-0.35161000
H	2.51147100	-0.72806000	1.39794000
C	-1.64103700	2.29757000	-0.45702600
H	-0.62427100	2.67409600	-0.61547700
C	3.36961800	0.44322200	-1.73162000
H	3.21372400	-0.48441500	-2.28412600
H	2.90566600	1.27624400	-2.25786600
C	-2.19270200	2.72451100	0.89474100
H	-3.21336200	2.35367200	1.02691400
H	-1.57527900	2.32674200	1.70324300
O	1.84851100	-0.66466800	2.38832600
H	0.86082000	-0.34274200	1.97899300
H	2.17025300	0.01072500	2.99891200
C	-1.74352700	-2.25006700	0.05955200
C	-3.09971600	-2.14816600	-0.61982200
H	-1.78836800	-1.92347400	1.10323100
H	-3.03235000	-2.47052100	-1.66278200
H	-3.46491700	-1.11876100	-0.59967200
H	2.61100500	1.22662200	0.20629900
H	-2.26457800	2.67365000	-1.27345200
H	-1.37058600	-3.27841700	0.04037400
H	4.44904000	0.62318000	-1.64593200
H	-2.21204600	3.81785000	0.96406300
H	-3.82375100	-2.78971900	-0.10542900

P_{DEP}

O	3.54351400	-0.26912300	0.58626200
P	-0.45248900	0.02759900	-0.18215300
O	-0.47541900	0.56635100	1.31751500

O	-1.92575800	0.40046000	-0.72002000
O	-0.51984300	-1.57162300	-0.12077900
O	0.67736600	0.49790300	-1.02675900
C	3.38539900	0.26361900	-0.50357200
H	2.57255500	0.43698800	1.85244100
C	-2.23379400	1.77122800	-1.07548100
H	-1.31361700	2.27935500	-1.38178900
C	3.70109400	-0.39873100	-1.80620700
H	4.15129600	-1.37963500	-1.64648600
H	2.75617900	-0.49456100	-2.35285600
C	-2.92525800	2.49485100	0.06865700
H	-3.84214300	1.97209200	0.35585700
H	-2.27251200	2.55080600	0.94280300
O	1.88081500	0.85968200	2.41777900
H	0.43759700	0.66172000	1.73748000
H	2.11798700	1.79433100	2.45914400
C	-1.44625500	-2.27880400	0.74166400
C	-2.65538100	-2.75891000	-0.04309900
H	-1.73483300	-1.63384800	1.57706100
H	-2.34512800	-3.39591300	-0.87619100
H	-3.21600700	-1.91101200	-0.44288900
H	2.98192600	1.28782800	-0.57456600
H	-2.88589400	1.70248600	-1.95077900
H	-0.87711100	-3.12120000	1.14520600
H	4.36264900	0.23723200	-2.40581600
H	-3.19108500	3.51181800	-0.23997200
H	-3.31473700	-3.34118600	0.60990000

Cartesian coordinates of reaction species for EHDPHP C_1-hydroxylation and O-dealkylation

^{2}RC

S	-4.66860200	-0.63854200	1.96548100
C	-2.56661900	2.93088200	1.54450800
C	-0.22527200	-1.11014700	2.79530900
C	-2.99816500	-3.64163300	-0.25244300
C	-5.00339400	0.46645000	-1.82606000
C	-1.70563600	2.04000400	2.17660200
C	-0.74932000	2.41245500	3.19129200
C	-0.06691000	1.28050700	3.51829200
C	-0.62610000	0.21858800	2.71861600
C	-0.79577400	-2.15824000	2.08694300
C	-0.40217200	-3.53908400	2.22059500
C	-1.19634600	-4.25445200	1.37904100

C	-2.06280000	-3.30601100	0.72202300
C	-3.80291700	-2.74530900	-0.93706300
C	-4.72465900	-3.10413400	-1.98653400
C	-5.27120700	-1.94558200	-2.44415400
C	-4.69633900	-0.87524200	-1.66716700
C	-4.51227700	1.50142400	-1.03961200
C	-4.92550700	2.87882400	-1.15385400
C	-4.26404300	3.56813600	-0.18475800
C	-3.43383500	2.61391100	0.50885200
N	-1.61729500	0.70123800	1.90611400
N	-1.81328300	-2.03749900	1.17412800
N	-3.81247200	-1.38181100	-0.74103500
N	-3.60277000	1.35961600	-0.02345900
Fe	-2.59815200	-0.31875400	0.47932500
H	-2.53340200	3.96696900	1.86605400
H	0.60777300	-1.33927800	3.44937600
H	-3.08726800	-4.69117700	-0.51430600
H	-5.71650900	0.72603600	-2.60187300
H	0.74248900	1.15276400	4.22351300
H	-0.62476400	3.41345600	3.58252900
H	0.38414100	-3.89164300	2.87370700
H	-1.20037400	-5.32078500	1.19683400
H	-4.90962900	-4.11577700	-2.32258400
H	-6.00122300	-1.80954700	-3.23096500
H	-5.63940500	3.24921000	-1.87743500
H	-4.31849100	4.62267100	0.05061300
O	-1.43984100	-0.12362200	-0.65203200
H	-4.60413500	-1.98301300	2.06285700
P	3.13830500	-0.79648800	0.83937700
O	2.81708600	0.31487300	-0.26356500
O	4.63618300	-1.23776600	0.40821400
O	2.91817600	-0.42478600	2.25451600
O	2.32180500	-2.13033800	0.44812500
C	2.20691800	-2.63292300	-0.85623800
C	1.06979800	-2.32162800	-1.59912500
C	3.19141100	-3.48965400	-1.34492400
C	0.92807400	-2.88283800	-2.87025700
H	0.31110500	-1.66039200	-1.19069100
C	3.03334900	-4.04270900	-2.61682400
H	4.05837600	-3.71013800	-0.73261100
C	1.90438500	-3.74078200	-3.38162600
H	0.04708500	-2.64377500	-3.45878800
H	3.79488600	-4.71166800	-3.00732800
H	1.78601700	-4.17313700	-4.37087600

C	5.73494800	-0.37672300	0.48353800
C	6.02933700	0.32963300	1.64988900
C	6.55975800	-0.30854400	-0.63735900
C	7.17408400	1.12858700	1.67761200
H	5.37005500	0.25020100	2.50685600
C	7.70423700	0.48762400	-0.58993700
H	6.29470400	-0.87427900	-1.52408400
C	8.01369900	1.20958800	0.56504900
H	7.41096300	1.68498700	2.57991700
H	8.35178000	0.54526700	-1.46000800
H	8.90416300	1.83008600	0.59783000
C	1.71564100	1.25841100	-0.07196800
C	1.77924600	2.35464900	-1.13585400
H	1.80978800	1.67991700	0.93267700
H	0.76864000	0.71261000	-0.14265000
C	0.70548000	3.40985300	-0.77608000
H	2.76877000	2.83049200	-1.06388800
C	0.89632700	4.77416600	-1.45240200
H	-0.28526000	3.00702500	-1.02666000
H	0.70036300	3.56568300	0.31158100
H	1.88061200	5.18003200	-1.17834200
H	0.90929100	4.65931000	-2.54410400
C	-0.19121600	5.78869400	-1.07424100
C	-0.00457600	7.15372300	-1.74460600
H	-0.20204300	5.91641300	0.01732000
H	-1.17476000	5.37858700	-1.34134000
H	-0.79356100	7.85560300	-1.45432700
H	0.95679500	7.60211500	-1.46891700
H	-0.02475700	7.06455900	-2.83680000
C	1.58177400	1.81794300	-2.57326800
C	2.81706700	1.18215000	-3.22410200
H	1.25059100	2.64760800	-3.20949500
H	0.75177000	1.09901900	-2.56193700
H	2.60073500	0.89678300	-4.25952800
H	3.65631500	1.88757300	-3.24375200
H	3.14414100	0.29015400	-2.68770900

^{4}RC

S	-4.64425200	-0.63608500	1.99353000
C	-2.55863700	2.92480400	1.55478500
C	-0.21855000	-1.12770300	2.76721600
C	-3.02294300	-3.63922300	-0.26755800
C	-5.04198300	0.48028700	-1.79631500
C	-1.69203400	2.02995400	2.17403700

C	-0.72170700	2.39643800	3.17807200
C	-0.04060300	1.26074300	3.49440500
C	-0.61442700	0.20313300	2.69872100
C	-0.79607600	-2.17107300	2.05754600
C	-0.40212300	-3.55301500	2.18032400
C	-1.20313200	-4.26340600	1.34104300
C	-2.07476700	-3.31072100	0.69705300
C	-3.83569300	-2.73752200	-0.93514900
C	-4.77724800	-3.09047900	-1.96860700
C	-5.32822400	-1.92919100	-2.41371100
C	-4.73615500	-0.86249900	-1.64508800
C	-4.53850300	1.51129000	-1.01246900
C	-4.95135200	2.88968200	-1.11370000
C	-4.27642100	3.57250000	-0.14933200
C	-3.43853700	2.61353200	0.52830900
N	-1.61260700	0.69226200	1.89952700
N	-1.82042800	-2.04505900	1.15284400
N	-3.83578900	-1.37361600	-0.73704200
N	-3.61554700	1.36224600	-0.00980500
Fe	-2.61393300	-0.32135800	0.47468700
H	-2.51994600	3.95967500	1.87965600
H	0.61898200	-1.36209100	3.41369400
H	-3.11906700	-4.68722100	-0.53310100
H	-5.76628600	0.74350000	-2.56044700
H	0.77765400	1.12758200	4.18825500
H	-0.58771300	3.39613800	3.56953500
H	0.38921300	-3.90947600	2.82520300
H	-1.20922400	-5.32872500	1.15305000
H	-4.97171100	-4.10078100	-2.30331600
H	-6.07202000	-1.78895700	-3.18674500
H	-5.67425300	3.26507800	-1.82564700
H	-4.32655000	4.62570500	0.09286300
O	-1.44167800	-0.11317100	-0.64219700
H	-4.59351000	-1.98209300	2.07601300
P	3.14875500	-0.79560900	0.83704800
O	2.83294200	0.31967000	-0.26348300
O	4.64657700	-1.23919900	0.40845800
O	2.92584900	-0.42784300	2.25283000
O	2.32962100	-2.12613500	0.43988200
C	2.21344100	-2.62315200	-0.86647300
C	1.07413100	-2.31078600	-1.60558700
C	3.19848900	-3.47584500	-1.36105300
C	0.93076600	-2.86649900	-2.87893300
H	0.31503600	-1.65303900	-1.19236000

C	3.03878600	-4.02343500	-2.63511800
H	4.06710000	-3.69758600	-0.75154500
C	1.90767000	-3.72022500	-3.39620300
H	0.04805000	-2.62661500	-3.46453500
H	3.80074200	-4.68921300	-3.03020200
H	1.78802700	-4.14836800	-4.38712300
C	5.74791400	-0.38207100	0.49177900
C	6.04143500	0.31633200	1.66311300
C	6.57601900	-0.31007500	-0.62642800
C	7.18887500	1.11110200	1.69874900
H	5.37949900	0.23413800	2.51776100
C	7.72312700	0.48180300	-0.57110900
H	6.31151800	-0.86964300	-1.51722300
C	8.03187100	1.19578500	0.58901600
H	7.42517000	1.66128100	2.60501000
H	8.37329200	0.54233800	-1.43902100
H	8.92439700	1.81294700	0.62797800
C	1.72987000	1.26169000	-0.07389600
C	1.79146600	2.35621500	-1.13959400
H	1.82303600	1.68518400	0.92996400
H	0.78366300	0.71442900	-0.14348500
C	0.71333800	3.40804000	-0.78291500
H	2.77914300	2.83575600	-1.06677600
C	0.89972300	4.77180300	-1.46155700
H	-0.27565500	3.00092300	-1.03355000
H	0.70658900	3.56582200	0.30445500
H	1.88271800	5.18130500	-1.18827300
H	0.91296800	4.65516200	-2.55307100
C	-0.19104100	5.78345700	-1.08496200
C	-0.00918500	7.14775600	-1.75810600
H	-0.20181000	5.91328700	0.00634800
H	-1.17335800	5.36959300	-1.35075300
H	-0.80037400	7.84757600	-1.46886900
H	0.95080800	7.59990600	-1.48373600
H	-0.02955300	7.05638000	-2.85011600
C	1.59851200	1.81656700	-2.57650900
C	2.83785800	1.18601800	-3.22475200
H	1.26411400	2.64369300	-3.21433800
H	0.77207400	1.09353800	-2.56556200
H	2.62436000	0.89793400	-4.26000500
H	3.67350100	1.89570600	-3.24440200
H	3.16874600	0.29654000	-2.68652000

^{2}TS$_{1\text{-}H}$

S	-4.33784700	-0.78257100	1.63234600
C	-3.48446900	2.84063300	0.04128500
C	-0.80165300	0.59726200	3.39163500
C	-1.50371800	-3.58464100	1.06658500
C	-3.81913000	-1.26688200	-2.48866800
C	-2.71215200	2.58341000	1.16450600
C	-2.38376800	3.57086500	2.16261100
C	-1.60185500	2.95138500	3.08908200
C	-1.46480400	1.57945600	2.66698700
C	-0.77020300	-0.74793500	3.05414100
C	-0.15751900	-1.78046600	3.85428500
C	-0.37846400	-2.95591400	3.20915100
C	-1.11521600	-2.64217200	2.00757600
C	-2.18743300	-3.32012900	-0.11375100
C	-2.52595400	-4.31492300	-1.10543400
C	-3.15737200	-3.66007700	-2.11742600
C	-3.21272500	-2.26647200	-1.73994300
C	-3.92729900	0.06505500	-2.10860100
C	-4.62792300	1.07556000	-2.86566700
C	-4.55520800	2.22671200	-2.14325300
C	-3.79992200	1.91994400	-0.95140700
N	-2.13010200	1.37645800	1.48177000
N	-1.34428400	-1.29063000	1.93339700
N	-2.61620500	-2.08532800	-0.51845900
N	-3.42888300	0.60384100	-0.95063900
Fe	-2.31918900	-0.33105300	0.46600000
H	-3.86499700	3.85024900	-0.07729600
H	-0.30307200	0.89701800	4.30698400
H	-1.23523500	-4.61775100	1.26256200
H	-4.26630900	-1.55326100	-3.43534200
H	-1.16697500	3.36327200	3.99012700
H	-2.71949600	4.59925200	2.14279300
H	0.37395900	-1.60533500	4.77950300
H	-0.06776700	-3.95171800	3.49564300
H	-2.29777700	-5.36920300	-1.02206000
H	-3.56191200	-4.06606900	-3.03533300
H	-5.11335200	0.90592800	-3.81774900
H	-4.96487900	3.19972500	-2.38031800
O	-0.91363000	-0.19173600	-0.55923600
H	-5.04744800	-1.29004900	0.60443100
P	2.77760500	-0.73011600	0.94668600
O	2.17720600	0.15012900	-0.28861300
O	4.29654200	-0.93970600	0.44266700
O	2.55621100	-0.15407200	2.28544300

O	2.19811800	-2.20889900	0.77232100
C	2.23540000	-2.94504700	-0.42737300
C	1.22200800	-2.77040600	-1.36623500
C	3.25111200	-3.88187200	-0.59619800
C	1.24001500	-3.56733600	-2.51380800
H	0.44228500	-2.03370900	-1.19526800
C	3.25175200	-4.66985200	-1.74829500
H	4.01738600	-3.98382900	0.16427400
C	2.24813900	-4.51442000	-2.70742600
H	0.45307900	-3.44765200	-3.25233000
H	4.03767400	-5.40533800	-1.89304500
H	2.25146400	-5.13084900	-3.60153600
C	5.20511300	0.12124700	0.32488500
C	5.50271800	0.93808500	1.41497300
C	5.84095800	0.27865100	-0.90442100
C	6.45850700	1.94342000	1.25460600
H	4.99140200	0.78473200	2.35848600
C	6.79894400	1.28340400	-1.04509700
H	5.58208400	-0.38253900	-1.72432500
C	7.10824500	2.11835100	0.03117900
H	6.69788200	2.58757300	2.09557600
H	7.30168800	1.41289800	-1.99894600
H	7.85344200	2.89975400	-0.08268100
C	1.17069600	1.10335200	-0.11275400
C	1.23801800	2.21303900	-1.14483700
H	1.07504300	1.40824000	0.93387100
H	-0.00652900	0.47197200	-0.28465600
C	0.31783700	3.37649500	-0.71356800
H	2.28427100	2.56766200	-1.10884800
C	0.69349500	4.73226600	-1.32829600
H	-0.71821300	3.12690300	-0.97085700
H	0.34248100	3.47841300	0.37909900
H	1.72615900	4.98449800	-1.04682400
H	0.68460900	4.66981800	-2.42432500
C	-0.24151200	5.86722000	-0.88954900
C	0.13595000	7.22567400	-1.48856300
H	-0.23892400	5.93476100	0.20680000
H	-1.27166100	5.61368600	-1.17465800
H	-0.54892700	8.01372300	-1.15850800
H	1.14928600	7.52152000	-1.19363600
H	0.10701600	7.19774000	-2.58390400
C	0.93741500	1.75425900	-2.59324300
C	2.00206900	0.87365700	-3.25830700
H	0.80572800	2.65625500	-3.20159000

H	-0.02839400	1.23615800	-2.58796500
H	1.75021000	0.70822900	-4.31146500
H	2.98938100	1.34927200	-3.22420400
H	2.08592500	-0.10295600	-2.77765300

$^{4}TS_{1\text{-}H}$

S	-4.25357000	-0.43294400	1.92310300
C	-2.51986500	3.13197600	1.16023600
C	-0.54103400	-0.75987900	3.23126800
C	-2.59275700	-3.58191600	-0.12393000
C	-4.22848500	0.34432500	-2.41386700
C	-1.79866100	2.32172400	2.02641000
C	-1.09040500	2.78589000	3.19638800
C	-0.51077800	1.69211500	3.76263400
C	-0.88008500	0.55820700	2.94888000
C	-0.97142600	-1.86977800	2.52073100
C	-0.66662600	-3.23201700	2.88111900
C	-1.26577600	-4.02907000	1.95572000
C	-1.92327600	-3.15412800	1.01631900
C	-3.16956900	-2.75070300	-1.07461100
C	-3.84192800	-3.21442200	-2.26719800
C	-4.28568900	-2.10811800	-2.92056000
C	-3.89376500	-0.96890600	-2.12212900
C	-3.93338800	1.44689000	-1.61761500
C	-4.32615500	2.79994100	-1.92204000
C	-3.86244700	3.58038000	-0.90596400
C	-3.17759300	2.70362000	0.01127900
N	-1.64974900	0.96322600	1.89058300
N	-1.74549700	-1.84519200	1.38264000
N	-3.19972100	-1.38220700	-1.01284000
N	-3.24277800	1.40667900	-0.43571200
Fe	-2.38971700	-0.22292300	0.39321800
H	-2.55649400	4.19322200	1.38519900
H	0.10052300	-0.93582000	4.08703600
H	-2.64957800	-4.65196900	-0.29530700
H	-4.78802100	0.52572900	-3.32608300
H	0.10440700	1.63613100	4.65042900
H	-1.05220000	3.81592700	3.52562200
H	-0.06451000	-3.51994400	3.73214000
H	-1.25780400	-5.10875600	1.88837300
H	-3.95406400	-4.25396800	-2.54553500
H	-4.84170500	-2.04827000	-3.84683800
H	-4.88481500	3.09731400	-2.79960600
H	-3.95935100	4.65061900	-0.77921600

O	-0.93591600	-0.06894100	-0.55840800
H	-4.67747400	0.84705900	1.87576300
P	2.72324400	-0.93133900	0.95206200
O	2.19548900	0.01222900	-0.24728700
O	4.21626200	-1.27952900	0.43446900
O	2.57383600	-0.38672800	2.31619200
O	2.01984800	-2.36045100	0.76701800
C	1.96477700	-3.06648500	-0.44672600
C	0.95648400	-2.77711100	-1.36342200
C	2.88638900	-4.08852800	-0.65895100
C	0.88283700	-3.54166500	-2.53051200
H	0.25257800	-1.97430700	-1.16266900
C	2.79535400	-4.84429800	-1.82886400
H	3.65348900	-4.27834600	0.08365900
C	1.79562000	-4.57253300	-2.76583300
H	0.10219400	-3.32725600	-3.25462400
H	3.50757900	-5.64501700	-2.00587600
H	1.72844200	-5.16245600	-3.67528300
C	5.21270000	-0.30711900	0.29789000
C	5.57431600	0.51172500	1.36748500
C	5.86928600	-0.23865200	-0.92917100
C	6.61448700	1.42597700	1.18846700
H	5.04626800	0.42824500	2.31069800
C	6.91142400	0.67515600	-1.08877100
H	5.55874500	-0.89675100	-1.73349200
C	7.28511000	1.51034400	-0.03328000
H	6.90335500	2.07009100	2.01389900
H	7.42928900	0.73426000	-2.04159500
H	8.09596000	2.22094400	-0.16196200
C	1.26960500	1.07190300	-0.04828000
C	1.47371200	2.17545600	-1.07512100
H	1.27011100	1.39425600	0.99585100
H	0.03259500	0.52873300	-0.22595500
C	0.69402200	3.43614200	-0.63766800
H	2.55142500	2.41335300	-1.04201400
C	1.21909900	4.74412700	-1.24605300
H	-0.36522500	3.30879400	-0.89338200
H	0.73290900	3.52786000	0.45572300
H	2.27169400	4.87987200	-0.95891100
H	1.20902100	4.68595300	-2.34229700
C	0.41406900	5.97521900	-0.80869100
C	0.94610200	7.28568700	-1.39757300
H	0.41799300	6.03915800	0.28824800
H	-0.63630600	5.84078200	-1.10145800

H	0.35158700	8.14458200	-1.06884300
H	1.98377900	7.46381300	-1.09298800
H	0.92365100	7.26563200	-2.49318200
C	1.11516400	1.76409100	-2.52406800
C	2.08744400	0.79555500	-3.20889200
H	1.05992100	2.67862200	-3.12646200
H	0.10618000	1.33512000	-2.51580900
H	1.81277400	0.66323600	-4.26127300
H	3.11469400	1.17791900	-3.17939200
H	2.08580300	-0.18806600	-2.73560500

^{2}IM$_{1\text{-}H}$

S	-4.58335500	-0.46174800	1.89623100
C	-2.37806300	2.95878600	1.59104600
C	-0.35248900	-1.21848400	2.93205400
C	-3.04869500	-3.59227600	-0.30447500
C	-4.70731600	0.63132500	-1.95457800
C	-1.57440100	2.02598000	2.24345000
C	-0.65435500	2.33434800	3.31245900
C	-0.06244300	1.15971300	3.66568400
C	-0.63895600	0.13847300	2.82391300
C	-0.94474100	-2.24038700	2.19583000
C	-0.63251600	-3.64145900	2.35047600
C	-1.40394800	-4.31258200	1.45175700
C	-2.17444600	-3.31554800	0.74645400
C	-3.73917000	-2.64859800	-1.05196100
C	-4.59927600	-2.94992300	-2.17407800
C	-5.04549100	-1.75819800	-2.65344500
C	-4.46795300	-0.73110700	-1.81490500
C	-4.23845300	1.63214400	-1.11212700
C	-4.59723400	3.02517400	-1.22561800
C	-3.97368600	3.67813900	-0.20688400
C	-3.21708000	2.68722600	0.51844500
N	-1.54056200	0.68975300	1.95065000
N	-1.89283300	-2.06727400	1.22488600
N	-3.67976400	-1.29209200	-0.84701200
N	-3.39729600	1.44416400	-0.04182900
Fe	-2.57917900	-0.29393800	0.53442900
H	-2.32010600	3.98893000	1.92860300
H	0.40681100	-1.50233100	3.65240000
H	-3.17709200	-4.63487800	-0.57905400
H	-5.35682400	0.93848300	-2.76856400
H	0.69297800	0.98009100	4.41798600
H	-0.49359800	3.32185800	3.72438800

H	0.09014200	-4.03834000	3.05049500
H	-1.44705300	-5.37657700	1.26065900
H	-4.81625100	-3.94630200	-2.53588400
H	-5.70854500	-1.57293200	-3.48815900
H	-5.25036300	3.43233500	-1.98609700
H	-4.00665500	4.73118500	0.03933500
O	-1.20161400	-0.22248500	-0.64229800
H	-4.78612900	-1.79022600	1.77759500
P	3.02875000	-0.82740700	0.83374200
O	2.67096300	0.25004100	-0.32814800
O	4.52177300	-1.25710500	0.39218000
O	2.82052500	-0.36458300	2.22024100
O	2.21920200	-2.16420000	0.49429400
C	2.12510800	-2.74918800	-0.78400200
C	1.02018800	-2.44896000	-1.57596200
C	3.09876600	-3.66367800	-1.17809800
C	0.90378500	-3.09027000	-2.81219600
H	0.26986600	-1.73799900	-1.23308300
C	2.96298600	-4.29440000	-2.41601600
H	3.93824000	-3.86938700	-0.52362300
C	1.86778800	-4.00863900	-3.23449500
H	0.04908600	-2.86572400	-3.44366900
H	3.71410800	-5.01036400	-2.73702000
H	1.76604100	-4.50190700	-4.19683300
C	5.61294300	-0.37939400	0.42475700
C	5.92229700	0.35406300	1.56977200
C	6.41015600	-0.32544100	-0.71622100
C	7.05657200	1.16835200	1.55499500
H	5.28397500	0.28416500	2.44319400
C	7.54440800	0.48667400	-0.71079800
H	6.13376200	-0.91455800	-1.58396900
C	7.86933800	1.23664400	0.42193100
H	7.30622400	1.74637000	2.43997000
H	8.17205900	0.53408500	-1.59581300
H	8.75197200	1.86898500	0.42174800
C	1.77132100	1.29311100	-0.11336600
C	1.71151100	2.33491000	-1.18730600
H	1.59843700	1.52832700	0.93366300
H	-0.58214900	0.46181100	-0.33941300
C	0.79064800	3.48248400	-0.70615200
H	2.73156400	2.74695200	-1.31182000
C	0.99682300	4.81478000	-1.43819300
H	-0.25573700	3.16480400	-0.80694900
H	0.95412000	3.65139700	0.36673400

H	2.03686700	5.14474400	-1.30319000
H	0.85866200	4.68093000	-2.51897400
C	0.04807500	5.91830000	-0.95128500
C	0.25506800	7.25414700	-1.67219200
H	0.18389100	6.06107600	0.12980300
H	-0.98998800	5.58463800	-1.08584400
H	-0.43515700	8.02037700	-1.30395800
H	1.27492700	7.62858000	-1.52745300
H	0.09227300	7.15115200	-2.75115200
C	1.26037400	1.78672600	-2.56957700
C	2.31252900	0.97560800	-3.33454900
H	0.96974900	2.64404400	-3.18837300
H	0.35341900	1.18672700	-2.42586800
H	1.94033600	0.71306400	-4.33070300
H	3.23532700	1.55304000	-3.46734300
H	2.56846400	0.04818400	-2.81903100

^{4}IM$_{1\text{-}H}$

S	-4.34689500	-0.65984600	2.03957700
C	-2.58988500	2.86173500	1.60479000
C	-0.26020900	-1.17614900	2.90767200
C	-2.79354800	-3.68911600	-0.36962600
C	-4.85595100	0.40973100	-1.90851700
C	-1.71230400	1.98462600	2.22520700
C	-0.80110200	2.34724900	3.28251500
C	-0.12611800	1.21604800	3.62987400
C	-0.63575500	0.15478300	2.79801900
C	-0.80609300	-2.21734500	2.17251900
C	-0.40703300	-3.59580100	2.30310900
C	-1.13355700	-4.30651900	1.39751900
C	-1.97045800	-3.36332900	0.69925400
C	-3.52447500	-2.77528300	-1.11193100
C	-4.36375300	-3.12371300	-2.23251900
C	-4.92931300	-1.96851700	-2.67620000
C	-4.44512400	-0.91075900	-1.82279600
C	-4.45757600	1.42048200	-1.04504400
C	-4.90751700	2.78651000	-1.12849000
C	-4.28598800	3.47036400	-0.12755700
C	-3.44565300	2.52696300	0.56550900
N	-1.57755100	0.65069600	1.93113900
N	-1.77695500	-2.10263300	1.20520600
N	-3.57616200	-1.42187000	-0.89223300
N	-3.57868900	1.28161100	-0.00092500
Fe	-2.61069000	-0.39844500	0.53223500

H	-2.59222400	3.89245700	1.94265900
H	0.52547100	-1.41618100	3.61404200
H	-2.84813800	-4.73255800	-0.66091400
H	-5.55770100	0.66853800	-2.69406700
H	0.64607900	1.08795300	4.37577800
H	-0.70524700	3.34320500	3.69447100
H	0.34611600	-3.95043100	2.99359000
H	-1.10102300	-5.36771700	1.18956600
H	-4.49349000	-4.12857400	-2.61219300
H	-5.62277700	-1.82694900	-3.49458500
H	-5.60654500	3.15786400	-1.86613000
H	-4.36850400	4.51900400	0.12577700
O	-1.25629000	-0.12145500	-0.62033900
H	-3.86846500	-1.74142800	2.68752700
P	3.13222000	-0.67169900	0.85768100
O	2.71298100	0.37766500	-0.30891400
O	4.63300900	-1.04405200	0.39471800
O	2.92403100	-0.20750500	2.24296400
O	2.36960000	-2.04276800	0.53629600
C	2.28685900	-2.63907500	-0.73577700
C	1.15599800	-2.40284300	-1.51300100
C	3.29870200	-3.50460200	-1.14424500
C	1.04931900	-3.05413900	-2.74424100
H	0.37720100	-1.73064200	-1.16370000
C	3.17432000	-4.14736100	-2.37713600
H	4.15878300	-3.66414800	-0.50395200
C	2.05254300	-3.92337100	-3.17853600
H	0.17300800	-2.87704800	-3.36066200
H	3.95590200	-4.82525800	-2.70776100
H	1.96030600	-4.42643100	-4.13660700
C	5.68958800	-0.12332400	0.41017300
C	5.99495100	0.61179200	1.55499100
C	6.45763800	-0.02801600	-0.74782500
C	7.09488700	1.47140900	1.52242500
H	5.37961500	0.50915200	2.44154500
C	7.55790000	0.82959100	-0.76030300
H	6.18600600	-0.62058100	-1.61474800
C	7.87791900	1.58240200	0.37190000
H	7.34116400	2.05153000	2.40692900
H	8.16272900	0.90999000	-1.65870000
H	8.73394600	2.25014600	0.35782000
C	1.72842700	1.34590800	-0.11095900
C	1.57763000	2.35529900	-1.20774300
H	1.55691200	1.60179300	0.93230000

H	-0.55236800	0.40199300	-0.19636300
C	0.51141900	3.39015100	-0.77299600
H	2.54427100	2.88608900	-1.30861100
C	0.56518500	4.72105900	-1.53389200
H	-0.48430900	2.94099000	-0.88354800
H	0.63131100	3.60164600	0.29806800
H	1.56005400	5.17203100	-1.40744400
H	0.44492600	4.54895800	-2.61137300
C	-0.50491600	5.71888900	-1.07053900
C	-0.45927400	7.04858500	-1.82980800
H	-0.38141200	5.90727900	0.00498900
H	-1.49709600	5.26204400	-1.18656800
H	-1.23103500	7.73991900	-1.47532000
H	0.51119700	7.54297300	-1.70638700
H	-0.61723600	6.89741800	-2.90382600
C	1.24108800	1.73409900	-2.59110900
C	2.41264000	1.06348100	-3.31800700
H	0.85503400	2.53626200	-3.23085700
H	0.41650500	1.02298900	-2.46005300
H	2.10187700	0.72786900	-4.31326200
H	3.24589900	1.76407300	-3.45023100
H	2.78862000	0.19530000	-2.77394600

^{4}TS$_{1\text{-}OH}$

S	-4.46884300	-1.03717300	1.64787400
C	-1.47872600	-3.67549300	0.94969900
C	-3.97459200	-1.24164200	-2.42140600
C	-3.62613500	2.78783900	0.24362300
C	-0.89215100	0.41358900	3.46602200
C	-2.24360800	-3.36923400	-0.16710000
C	-2.60969700	-4.31987000	-1.18532900
C	-3.29717200	-3.63340700	-2.14050600
C	-3.36068400	-2.26075200	-1.70677100
C	-4.05372100	0.08126100	-2.01050800
C	-4.78051900	1.11074400	-2.70954800
C	-4.69327000	2.24027600	-1.95317200
C	-3.90737200	1.90789800	-0.79172200
C	-2.89054000	2.47453300	1.37959600
C	-2.52268800	3.42653100	2.39803000
C	-1.73901300	2.76337500	3.29430100
C	-1.63426300	1.40184300	2.83233600
C	-0.78128300	-0.89836700	3.03241900
C	-0.06875000	-1.93210400	3.74543100
C	-0.25083600	-3.08593800	3.05060100

C	-1.06354600	-2.75891600	1.90300200
N	-2.72154900	-2.12471500	-0.49823800
N	-3.50456100	0.59488600	-0.86044700
N	-2.35898400	1.24472500	1.67741900
N	-1.36216700	-1.42183800	1.90698900
Fe	-2.46857900	-0.43277400	0.55104900
H	-1.16832500	-4.70705800	1.07579000
H	-4.45211500	-1.50521700	-3.35916300
H	-3.99884500	3.80269900	0.15340800
H	-0.36409300	0.68690300	4.37290000
H	-3.72755100	-4.00636600	-3.06060500
H	-2.35421400	-5.37089100	-1.16274000
H	-5.29999000	0.96638900	-3.64767900
H	-5.12446100	3.21416200	-2.14419500
H	-2.82221000	4.46631600	2.40385600
H	-1.26447300	3.14593900	4.18829800
H	0.49454000	-1.77127300	4.65430500
H	0.12893900	-4.07447900	3.27133700
O	-0.88189500	0.01525900	-0.30186200
H	-4.48914000	-0.03376700	2.54823900
P	2.94029900	-0.67121100	0.86392200
O	2.53487300	0.34756700	-0.34366000
O	4.47783400	-0.97243200	0.46845200
O	2.65121800	-0.19008200	2.22674600
O	2.26887700	-2.08129400	0.52218800
C	2.21916800	-2.65197100	-0.76190400
C	1.11566100	-2.38670700	-1.56849700
C	3.23377100	-3.52187500	-1.15212900
C	1.03741600	-3.02009800	-2.81098800
H	0.34823500	-1.70127000	-1.21755500
C	3.13859500	-4.14483000	-2.39803500
H	4.07196000	-3.70086600	-0.48800200
C	2.04287200	-3.89611200	-3.22769200
H	0.18011100	-2.83006700	-3.45006800
H	3.92166400	-4.82680900	-2.71650300
H	1.97216300	-4.38570300	-4.19458700
C	5.48436200	-0.00010100	0.53108800
C	5.70758600	0.74081100	1.69127000
C	6.29139900	0.14414900	-0.59517600
C	6.76241400	1.65559200	1.70683600
H	5.06302300	0.60155500	2.55160500
C	7.34582600	1.05697100	-0.55979300
H	6.08505100	-0.45535000	-1.47528300
C	7.58287900	1.81595800	0.58858500

H	6.94393400	2.24046700	2.60381000
H	7.98031400	1.17490000	-1.43328300
H	8.40352200	2.52650700	0.61222300
C	1.50426900	1.25789200	-0.21912700
C	1.45392600	2.34172700	-1.24446600
H	1.08302900	1.36042500	0.77232400
H	-1.13251800	0.54294500	-1.07664000
C	0.32618900	3.33509300	-0.87522800
H	2.41584300	2.88835500	-1.18912800
C	0.49531900	4.73456300	-1.48069200
H	-0.64023100	2.91457000	-1.18189700
H	0.27343200	3.43438700	0.21630500
H	1.44273500	5.16761500	-1.12921500
H	0.57923400	4.67052600	-2.57385500
C	-0.65656200	5.68441500	-1.12608900
C	-0.47959200	7.09033600	-1.70834800
H	-0.74863900	5.74751100	-0.03321900
H	-1.60127700	5.25317600	-1.48424000
H	-1.31636400	7.74396800	-1.44067400
H	0.43949300	7.55919700	-1.33863000
H	-0.41737500	7.06152400	-2.80225500
C	1.31305100	1.82972300	-2.70507200
C	2.57059500	1.20023200	-3.31500900
H	1.01212100	2.68040200	-3.32783900
H	0.48448100	1.11101000	-2.74132100
H	2.40095600	0.95268400	-4.36838700
H	3.41803500	1.89418900	-3.26987600
H	2.85916300	0.28469100	-2.79583700

^{2}IM$_{1\text{-OH}}$

S	-4.37868100	-1.58190400	0.98696000
C	-1.78636500	-3.48494100	-1.58585000
C	-3.88587400	0.81617900	-2.32059200
C	-2.88669600	2.13228500	2.22998000
C	-0.82920200	-2.18401700	2.97339400
C	-2.40985100	-2.40485100	-2.19685200
C	-2.88627000	-2.39843600	-3.55644900
C	-3.48085700	-1.18972900	-3.75928300
C	-3.36852900	-0.45588000	-2.52369600
C	-3.81421200	1.53561900	-1.13501900
C	-4.36144700	2.85903500	-0.95758500
C	-4.08023200	3.23318100	0.32165600
C	-3.36037600	2.13893900	0.92444900
C	-2.20741300	1.07521000	2.81789200

C	-1.73563200	1.06476600	4.18100400
C	-1.15989700	-0.14997200	4.38643300
C	-1.28181900	-0.88496500	3.15106700
C	-0.93371300	-2.91817300	1.80105300
C	-0.45393300	-4.27147800	1.64754400
C	-0.72417600	-4.64128600	0.36717600
C	-1.36526500	-3.51170100	-0.26353000
N	-2.69139800	-1.20314700	-1.58530700
N	-3.20625600	1.11245300	0.02346400
N	-1.91018800	-0.11809500	2.19932900
N	-1.48306000	-2.47048500	0.62571800
Fe	-2.42036000	-0.71098300	0.35617100
H	-1.63544900	-4.38031200	-2.18037900
H	-4.39870100	1.28319100	-3.15550700
H	-3.06999700	3.01303500	2.83700000
H	-0.34779500	-2.66215300	3.81977800
H	-3.96298600	-0.81806900	-4.65402200
H	-2.78126000	-3.22260300	-4.24986200
H	-4.89013600	3.41391800	-1.72165400
H	-4.32879300	4.15883000	0.82386900
H	-1.83747400	1.88967200	4.87382500
H	-0.68472700	-0.52940600	5.28102200
H	0.03004500	-4.84373600	2.42773700
H	-0.50912700	-5.58112400	-0.12377500
O	-0.33535300	0.08195100	-0.49136600
H	-4.80190200	-0.55158700	1.74984100
P	2.66907700	-0.42080300	1.14154300
O	1.97264800	0.14920300	-0.19519700
O	4.23722800	-0.12492000	0.84468900
O	2.11175300	0.07171700	2.41588300
O	2.66714000	-2.02332900	1.02414000
C	3.13728500	-2.72488700	-0.09441500
C	2.26531500	-2.99308500	-1.14680500
C	4.44750400	-3.19717800	-0.09084800
C	2.72684600	-3.74966300	-2.22565500
H	1.24795900	-2.62279700	-1.10489400
C	4.89399600	-3.95461700	-1.17487300
H	5.09395900	-2.96902500	0.74907600
C	4.03786000	-4.23070200	-2.24342400
H	2.05478200	-3.96640000	-3.05089900
H	5.91334500	-4.32932300	-1.18143600
H	4.38993600	-4.82107900	-3.08401900
C	4.72716100	1.16676800	0.64113800
C	4.44310500	2.20448700	1.52991900

C	5.56062100	1.35947900	-0.45980800
C	5.00019200	3.46299500	1.29121400
H	3.80445800	2.02020100	2.38678100
C	6.11685900	2.62027300	-0.67780700
H	5.76231000	0.52433900	-1.12205200
C	5.83574200	3.67577800	0.19318900
H	4.78351300	4.27733300	1.97649100
H	6.76893500	2.77624500	-1.53223500
H	6.26841900	4.65622600	0.01902700
C	0.73584100	0.91758700	-0.15232300
C	0.90512400	2.14640200	-1.06462200
H	0.56170400	1.21147100	0.88355400
H	-0.36923600	-0.08577600	-1.44422200
C	-0.17528200	3.19427900	-0.71069400
H	1.88506400	2.56435700	-0.79836100
C	0.09793900	4.60527900	-1.25148700
H	-1.15173900	2.84501200	-1.06654400
H	-0.26080300	3.25617100	0.38208300
H	1.10477400	4.92603700	-0.94673000
H	0.09935200	4.60155500	-2.34929600
C	-0.92975400	5.63723800	-0.76666600
C	-0.68585200	7.04063800	-1.33024600
H	-0.91255000	5.67409200	0.33114500
H	-1.93725200	5.29779500	-1.04049400
H	-1.43032700	7.75486600	-0.96328700
H	0.30359700	7.41682900	-1.04562500
H	-0.73588700	7.04253900	-2.42526900
C	0.91247000	1.82194900	-2.57565000
C	2.07779100	0.96953600	-3.09745600
H	0.92275800	2.77731900	-3.11007500
H	-0.04854000	1.36257600	-2.85465900
H	2.04010300	0.90890400	-4.19036900
H	3.03989300	1.41172100	-2.81846800
H	2.06684500	-0.04886200	-2.70247900

^{4}IM$_{1\text{-OH}}$

S	-4.82067000	-0.76589400	1.94085500
C	-0.87767100	0.56299300	3.38340400
C	-1.79403300	-3.58836700	1.07491400
C	-4.05752500	-1.12726900	-2.42487800
C	-3.58628700	2.94387100	0.15824600
C	-0.92020900	-0.77893700	3.04113500
C	-0.26354900	-1.82223000	3.78516500
C	-0.55141600	-2.99400200	3.15687800

C	-1.36616100	-2.66476500	2.01579700
C	-2.46524600	-3.26794800	-0.09584000
C	-2.82486800	-4.22423000	-1.11094900
C	-3.43585200	-3.52709400	-2.10985300
C	-3.46232900	-2.14725900	-1.69860100
C	-4.14177100	0.19273000	-2.00672900
C	-4.79979700	1.23550800	-2.74727100
C	-4.69051500	2.37514100	-2.00749000
C	-3.95315200	2.03287800	-0.82101200
C	-2.78929400	2.64527100	1.25198900
C	-2.33071500	3.61943800	2.20953200
C	-1.54053000	2.95514300	3.09766300
C	-1.53248700	1.57202000	2.69391600
N	-1.59207100	-1.30795900	1.96346900
N	-2.85234900	-2.00155200	-0.47272900
N	-3.62765900	0.69329600	-0.82965600
N	-2.29003700	1.40059800	1.55877100
Fe	-2.79008900	-0.34347200	0.67705100
H	-0.29984700	0.84026300	4.25803600
H	-1.54694100	-4.63091200	1.24488600
H	-4.50922600	-1.38335100	-3.37754500
H	-3.91443500	3.97087000	0.03853300
H	-0.22772200	-3.99392300	3.41281600
H	0.34772400	-1.65772000	4.66170100
H	-2.62915700	-5.28676200	-1.05143300
H	-3.84797800	-3.90015500	-3.03830600
H	-5.28130400	1.09864900	-3.70659800
H	-5.06092800	3.36559400	-2.23677400
H	-2.58527700	4.67084700	2.18733000
H	-1.01367900	3.34692300	3.95764400
O	0.28250800	-0.04134900	-0.58668200
H	-5.64119200	-0.68573600	0.87237600
P	3.05861700	-0.78444000	0.85371200
O	2.61029200	0.11166400	-0.40751400
O	4.65647600	-0.89252500	0.59132900
O	2.62172600	-0.31895500	2.18426100
O	2.61962800	-2.30277200	0.54465700
C	2.54498000	-2.87775000	-0.72700800
C	1.27989500	-3.20440600	-1.21346900
C	3.70163000	-3.18023200	-1.44369500
C	1.17349400	-3.83946600	-2.45217100
H	0.40615900	-2.97079800	-0.61592300
C	3.57858000	-3.81134600	-2.68374700
H	4.67075600	-2.92738600	-1.02974300

C	2.31994100	-4.14086800	-3.19138700
H	0.18939100	-4.09926200	-2.83165600
H	4.47382000	-4.05101400	-3.25017800
H	2.23377300	-4.63495900	-4.15445200
C	5.51357700	0.20332400	0.74700900
C	5.58517200	0.89818500	1.95433300
C	6.33875900	0.52442400	-0.32888100
C	6.50460900	1.94225200	2.07217800
H	4.92719200	0.62357600	2.77055600
C	7.25748400	1.56564100	-0.19220800
H	6.25288100	-0.04092800	-1.25063500
C	7.34206200	2.27761500	1.00634800
H	6.56763300	2.49092600	3.00739300
H	7.90578600	1.81992900	-1.02564700
H	8.05761900	3.08774500	1.10941700
C	1.34426100	0.83988400	-0.39058800
C	1.44816400	1.97572000	-1.42365700
H	1.20759000	1.24071800	0.61596200
H	0.34199100	-0.41846600	-1.47631300
C	0.29230000	2.97361300	-1.17607100
H	2.39555300	2.48672700	-1.20361200
C	0.50736300	4.36246400	-1.79387600
H	-0.64582600	2.54114000	-1.54616500
H	0.15426000	3.09906900	-0.09434900
H	1.44912900	4.78534800	-1.41528000
H	0.62625000	4.28545000	-2.88276800
C	-0.63968000	5.33613800	-1.49176600
C	-0.42551900	6.72624200	-2.09910200
H	-0.76011500	5.42400900	-0.40354400
H	-1.58050100	4.91045100	-1.86592000
H	-1.25797300	7.39855100	-1.86652500
H	0.49152700	7.18880000	-1.71647800
H	-0.33662300	6.67313500	-3.19031400
C	1.48178500	1.49684700	-2.89281800
C	2.76929900	0.80717300	-3.36407900
H	1.31129800	2.37315600	-3.52707600
H	0.61404300	0.84691100	-3.08258200
H	2.72552400	0.61901800	-4.44224500
H	3.64177000	1.44142500	-3.17220700
H	2.94213300	-0.14598200	-2.86065100

$IM_{1\text{-}OH}$

O	-0.91449400	-2.38226700	1.11450600
P	1.19343700	0.17998800	1.14547300

O	0.12387000	-0.46827700	0.16596100
O	1.65102700	1.53492500	0.39753300
O	0.76345000	0.32481600	2.56284000
O	2.55266400	-0.67470100	1.06340200
C	3.42673200	-0.80499800	-0.02092200
C	4.78312400	-0.84634700	0.29389800
C	2.98053500	-0.94278500	-1.33391300
C	5.71321700	-1.02323300	-0.72961000
H	5.08897000	-0.73838000	1.32874600
C	3.92543800	-1.11255300	-2.34769600
H	1.92079600	-0.92725500	-1.55628200
C	5.28867400	-1.15422900	-2.05318300
H	6.77158800	-1.05536100	-0.48916000
H	3.58632700	-1.22019500	-3.37369400
H	6.01487200	-1.28986100	-2.84848400
C	0.74900800	2.56787700	0.11206400
C	0.04647600	3.20618800	1.13303600
C	0.63603300	2.96927900	-1.21636700
C	-0.79809900	4.26700600	0.80120100
H	0.16500300	2.87287900	2.15780000
C	-0.20615500	4.03612800	-1.52991100
H	1.20655800	2.44870000	-1.97764200
C	-0.92616500	4.68493000	-0.52446400
H	-1.35191700	4.77128700	1.58736800
H	-0.29942900	4.35814300	-2.56267400
H	-1.58136500	5.51405200	-0.77289800
C	-1.15333400	-1.10898300	0.69674600
C	-2.17267700	-1.06138900	-0.44873700
H	-1.45161000	-0.44736900	1.51664600
H	-0.60341700	-2.37036500	2.05622900
C	-3.58610200	-1.05635200	0.18224300
H	-2.02299700	-0.09998500	-0.95791300
C	-4.74017000	-0.80500100	-0.79724000
H	-3.74343400	-2.00886700	0.70553300
H	-3.62020000	-0.27582300	0.95517700
H	-4.53375700	0.10617900	-1.37740300
H	-4.80732900	-1.62225200	-1.52598300
C	-6.09633600	-0.65904500	-0.09433000
C	-7.25580900	-0.41566900	-1.06490600
H	-6.04322200	0.16737500	0.62729800
H	-6.29602500	-1.56346000	0.49601800
H	-8.20764600	-0.31297000	-0.53378700
H	-7.09976000	0.49966900	-1.64719400
H	-7.35948200	-1.24391600	-1.77508500

C	-2.00775600	-2.20740200	-1.46666000
C	-0.66227700	-2.27792200	-2.19576000
H	-2.80330900	-2.10592400	-2.21289900
H	-2.18604100	-3.15546000	-0.94699300
H	-0.68536200	-3.06144700	-2.96077500
H	-0.42794800	-1.33152500	-2.69679100
H	0.15113600	-2.50766100	-1.50466300
O	0.01263200	-2.07360200	3.63208600
H	0.82970400	-2.57115700	3.76485700
H	0.30840300	-1.14902400	3.47897800

TS_{DPHP}

O	-1.07009500	-2.69180400	1.38494600
P	1.51818800	-0.05297700	1.14471600
O	0.66839700	-0.88010200	0.19408200
O	1.42696700	1.53546000	0.73023300
O	1.26713700	-0.21567600	2.63162400
O	3.10822100	-0.28360000	0.91479600
C	3.78873800	-0.09463100	-0.28495600
C	5.02180300	0.55204600	-0.20316100
C	3.30991200	-0.58187800	-1.50214400
C	5.78439300	0.71904500	-1.35827400
H	5.36213000	0.91391600	0.76098400
C	4.08194600	-0.39949700	-2.65087000
H	2.35180300	-1.08651500	-1.53629400
C	5.31741800	0.24651100	-2.58650800
H	6.74461100	1.22283200	-1.29630700
H	3.71353800	-0.77410100	-3.60172900
H	5.91237700	0.37945000	-3.48495100
C	0.18176500	2.13466200	0.60849800
C	-0.63680400	2.31176600	1.72772500
C	-0.20961100	2.60031100	-0.64741400
C	-1.86685300	2.95498300	1.57512900
H	-0.29674800	1.95218000	2.69264600
C	-1.43824900	3.24771000	-0.78397900
H	0.45358500	2.45109100	-1.49260400
C	-2.27207700	3.42361800	0.32342300
H	-2.50423200	3.09967000	2.44280900
H	-1.74283700	3.61691600	-1.75906100
H	-3.22548600	3.93125900	0.21304200
C	-1.30347900	-1.56202100	0.87514800
C	-2.05027300	-1.42513000	-0.41542500
H	-1.24189000	-0.67602000	1.51058600
H	-0.50783000	-2.58278400	2.37519700

C	-3.50650000	-1.01374700	-0.03855200
H	-1.58869900	-0.57495500	-0.93051100
C	-4.26104200	-0.32790000	-1.18584400
H	-4.05189500	-1.90629900	0.29525000
H	-3.48822800	-0.32355300	0.81570900
H	-3.72005100	0.58549900	-1.46736800
H	-4.26402900	-0.97253700	-2.07407000
C	-5.70746200	0.02852900	-0.82035700
C	-6.45624900	0.73114500	-1.95688900
H	-5.70800200	0.67045600	0.07087400
H	-6.24579600	-0.88584900	-0.53678700
H	-7.48442700	0.97169300	-1.66856400
H	-5.96149200	1.66765400	-2.23763900
H	-6.50095700	0.10050000	-2.85193900
C	-1.99567000	-2.68626100	-1.29399300
C	-0.62875500	-2.96397000	-1.92786800
H	-2.74599400	-2.58099000	-2.08556100
H	-2.30625500	-3.54520700	-0.68766900
H	-0.66713700	-3.87410300	-2.53526300
H	-0.32214300	-2.13819700	-2.57849600
H	0.14611500	-3.08674700	-1.16866800
O	0.18126000	-2.30636200	3.41854700
H	0.86033300	-2.97701300	3.57121300
H	0.68400300	-1.41865900	3.15845200

P_{DPHP}

O	-1.66105800	-2.08254700	2.31971800
P	1.60361100	0.27479600	0.95532600
O	0.40837400	-0.36745400	0.34757400
O	2.04680900	1.68038700	0.28322200
O	1.55340300	0.58160200	2.49852800
O	2.99107500	-0.54775500	0.81435100
C	3.52075500	-0.97885800	-0.40459800
C	4.87397000	-0.72562000	-0.62136700
C	2.75741100	-1.69000800	-1.33067100
C	5.47381100	-1.18906200	-1.79180700
H	5.43306500	-0.17184800	0.12490700
C	3.37154700	-2.14025300	-2.50076300
H	1.70801500	-1.87272000	-1.13274600
C	4.72537700	-1.89542600	-2.73561900
H	6.52794200	-0.99410600	-1.96540200
H	2.78442700	-2.69214100	-3.22904700
H	5.19476200	-2.25359300	-3.64671100
C	1.12024800	2.69723000	0.03814200

C	0.58017900	3.42676000	1.09661200
C	0.81532100	3.00334300	-1.28597700
C	-0.29191900	4.47910700	0.81359400
H	0.84850000	3.16893600	2.11495800
C	-0.05338500	4.06145500	-1.55393900
H	1.26070800	2.41615200	-2.08160800
C	-0.61019800	4.79970500	-0.50746400
H	-0.71737000	5.05349900	1.63123200
H	-0.29480200	4.30758000	-2.58376000
H	-1.28538400	5.62275800	-0.72075200
C	-1.99108900	-1.23937000	1.49652300
C	-2.73738900	-1.52820200	0.21877400
H	-1.77195000	-0.17171100	1.67401500
H	-0.44553600	-1.54755300	3.42512300
C	-4.07526800	-0.74224600	0.25993400
H	-2.11031000	-1.06968800	-0.56218300
C	-4.73355900	-0.54710300	-1.11243600
H	-4.77004400	-1.25941800	0.93579400
H	-3.89884300	0.24810000	0.70241200
H	-4.02834200	-0.02965000	-1.77763600
H	-4.93346900	-1.52144500	-1.57671900
C	-6.04306200	0.24867900	-1.04029600
C	-6.70272100	0.44455800	-2.40856500
H	-5.84675900	1.22830800	-0.58415100
H	-6.74208000	-0.26524700	-0.36667300
H	-7.63307800	1.01535300	-2.32564200
H	-6.04059700	0.98541600	-3.09406000
H	-6.94362400	-0.51799300	-2.87389100
C	-2.90469200	-3.03133400	-0.04993100
C	-1.60518800	-3.73937600	-0.44879800
H	-3.64646500	-3.16800500	-0.84468100
H	-3.31979100	-3.50137800	0.84951900
H	-1.78189400	-4.80499200	-0.62731200
H	-1.18949400	-3.31219000	-1.36831700
H	-0.84902800	-3.65008800	0.33489600
O	0.34944300	-1.20853700	3.90788500
H	0.92418500	-1.97595400	4.02352500
H	1.06780900	-0.11777500	3.05670700
